# Supplementary material for: Characterization of paramagnetic states in an organometallic nickel hydrogen evolution electrocatalyst
Source: Nat Commun. 2023 Feb 17;14:905. doi: 10.1038/s41467-023-36609-7 (PMC9938211; doi:10.1038/s41467-023-36609-7)
Supplement: Supplementary file 1 — Supplementary Information [file 41467_2023_36609_MOESM1_ESM.pdf]

# Supplementary Information

# Characterization of Paramagnetic States in an Organometallic Nickel Hydrogen Evolution Electrocatalyst

*Sagnik Chakrabarti,[1,#] Soumalya Sinha,[1,#] Giang N. Tran,[1,#] Hanah Na,[1] and Liviu M. Mirica[1,]**

[1] Department of Chemistry, University of Illinois at Urbana-Champaign, Urbana, Illinois, 61801

[#] These authors contributed equally.

*E-mail: mirica@illinois.edu

## Table of Contents

**1. General experimental details** ........ **S4**

**Reagents and Materials** ........ S4

**Physical Measurements** ........ S4

**2. Syntheses of ligands and Ni complexes** ........ **S5**

**Preparation of NCHS2 ligand** ........ S5

**Preparation of NCBrS2 ligand** ........ S7

**NMR Studies of (NCHS2)NiOTf$_2$** ........ S9

**Preparation of NCDS2** ........ S12

**H/D exchange in (NCDS2)NiOTf$_2$** ........ S14

**MALDI-TOF Mass Spectrometry studies for 1 and 2-Br** ........ S16

**3. Electrochemical studies** ........ **S17**

**Cyclic voltammograms (CVs) for bare glassy carbon (GC) electrode** ........ S17

**Cyclic voltammograms (CVs) for (NCHS2)Ni(OTf)$_2$ , 1(OTf)$_2$** ........ S18

**Peak Shift Analysis** ........ S20

**Cyclic voltammograms (CVs) for [(NCS2)Ni(MeCN)$_2$](OTf), 2(OTf)** ........ S24

**Table S1.** Cathodic peak current densities for **1** and **2** in the presence of TFA ........ S27

**Cyclic Voltammograms for [(NCS2)Ni$^{III}$(MeCN)][OTf]$_2$ ([2]$^+$)** ........ S27

**Cyclic Voltammograms for [(NCS2)Ni(μ-Br)]$_2$ (2-Br)** ........ S28

**Cyclic Voltammograms for [(N2S2)Ni(MeCN)$_2$][BF$_4$]$_2$** ........ S30

**Control experiments for checking the homogeneity of 1** ........ S31

**Surface analysis of a post-rinse glassy carbon electrode:** ........ S34

**Electrolysis experiments data** ........ S37

**4. Gas chromatography data** ........ **S39**

**5. Overpotential, Kinetics and Electrochemical Mechanistic Experiments** ........ **S40**

**Calculation of Overpotential** ........ S40

**Kinetic Analysis** ........ S40

**Determination of order with respect to catalyst** ........ S42

**Determination of order with respect to acid** ........ S43

**Kinetic Isotope Effect Experiments** ........ S45

**Table S3.** Calculated electrochemical kinetic isotope effects. ........ S48

**6. Absorption spectra** ........ **S51**

**7. EPR studies of Ni complexes** ........ **S54**

**Table S4:** Differences between simulation parameters of the EPR scale reduction of (NCHS2)$Ni^{II}OTf_2$ vs (NCDS2)$Ni^{II}OTf_2$ with $CoCp*_2$ ........ S58

**Table S5:** Comparison of the simulation parameters of **1** + $CoCp^*_2$ and **[2]**$^+$ + NaOPh + HBPin. ........ S62

**8. Infrared Spectroscopy Data** ........ **S63**

**9. ESI-MS analysis of the post-electrolysis solutions** ........ **S66**

**10. X-ray crystal structure characterization** ........ **S70**

**X-ray structure determination of NCHS2** ........ S71

**Table S6.** Crystal data and structure refinement for NCHS2. ........ S71

**Table S7.** Bond lengths [Å] and angles [°] for NCHS2 are included in Supplementary Data 1 ........ S72

**X-ray structure determination of (NCHS2)Ni(OTf)$_2$, 1** ........ S73

**Table S8.** Crystal data and structure refinement for **1** ........ S73

**Table S9.** Bond lengths [Å] and angles [°] for **1** are included in Supplementary Data 2. ........ S74

**X-ray structure determination of [(NCS2)Ni($\mu$-Br)]$_2$, 2-Br** ........ S75

**Table S10.** Crystal data and structure refinement for [(NCS2)Ni($\mu$-Br)]$_2$ ........ S75

**Table S11.** Bond lengths [Å] and angles [°] for [(NCS2)Ni($\mu$-Br)]$_2$ ........ S76

**X-ray structure determination of [(NCS2)Ni(MeCN)]$_2$[SbF$_6$][OTf], [2]$^+$** ........ S80

**Table S11.** Crystal data and structure refinement for [(NCS2)Ni(MeCN)]2[SbF6][OTf], [2]+ ........ S80

**Table S12.** Bond lengths [Å] and angles [°] for [(NCS2)Ni(MeCN)$_2$][SbF$_6$][OTf] ........ S81

**X-ray structure determination of [(NCS2)Ni(MeCN)(Br)][OTf], [2-Br]$^+$** ........ S86

**Table S13.** Crystal data and structure refinement for [(NCS2)Ni(MeCN)(Br)][OTf] ........ S86

**Table S14.** Bond lengths [Å] and angles [°] for [(NCS2)Ni(Br)(MeCN)][OTf] ........ S87

**11. DFT Calculations** ........ **S92**

(a) Frontier orbitals of [(NCHS2)$Ni^I$(MeCN)]$^+$, **3** ........ S93

(b) DFT optimized coordinates of the intermediates discussed in the main text ............................... S94

(c) Energy of other reactions in the electrocatalytic cycle .............................................................. S105

**12. Supplementary References........................................................................................................ S108**

## 1. General experimental details

### Reagents and Materials

All reagents were commercially available from Sigma-Aldrich, Fisher Scientific or Strem Chemicals and were used as received without further purification. 2-bromo-1,3-bis(bromomethyl)benzene was synthesized following a published procedure.[1,2] Solvents were purified prior to use by passing through a column of activated alumina using an MBraun solvent purification system.

### Physical Measurements

$^1$H NMR spectra were recorded on a Bruker 500 spectrometer (500 MHz) at UIUC School of Chemical Sciences NMR Lab. Chemical shifts are reported in ppm and referenced to residual solvent resonance peaks. UV-vis spectra were recorded on a Varian Cary 50 Bio spectrophotometer and are reported as $\lambda_{max}$, nm ($\varepsilon$, $M^{-1}$ $cm^{-1}$). EPR spectra were recorded on a JEOL JES-FA X-band (9.2 GHz) or a Bruker 10" EMXPlus X-band Continuous Wave EPR spectrometer at 77 K. EPR spectra simulation and analysis were performed using Bruker WINEPR SimFonia program, version 1.25. Elemental analysis was carried out by the Microanalysis Laboratory at UIUC using an Exeter Analytical Model CE440 CHN Analyzer. Solid-state infrared spectra were measured using a PerkinElmer Frontier FT-IR spectrophotometer equipped with a KRS5 thallium bromide/iodide universal attenuated total reflectance accessory. Cyclic voltammetry (CV) was performed using a BASi EC Epsilon electrochemical workstation or a CHI Electrochemical Analyzer 660D. Measurements were taken in a glove box under nitrogen. Glassy carbon disk electrode (d = 1.6 mm) was used as the working electrode for cyclic voltammetry and the auxiliary electrode was a platinum wire. Ag/$AgNO_3$ (0.1 M) was used as the reference electrode. The reference was calibrated against ferrocene after each experiment. ESI-MS experiments were performed by the Mass Spectrometry Lab at UIUC using a Waters Q-TOF Ultima ESI mass spectrometer with an electron spray ionization source.

## 2. Syntheses of ligands and Ni complexes

### Preparation of NCHS2 ligand

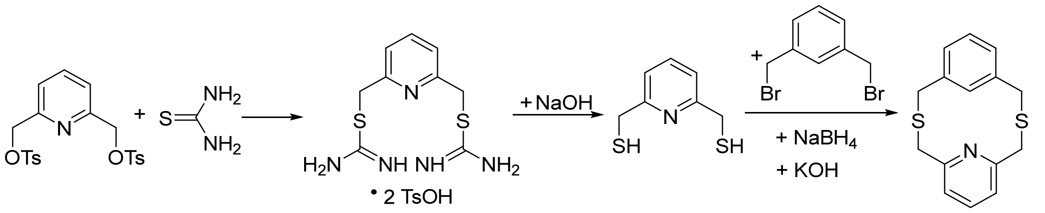


A B C NCHS2

Compounds **A**, **B**, and **C** were synthesized according to a published procedure.[3,4] The synthesis was performed under $N_2$ atmosphere unless otherwise specified. In a round bottom flask, sodium borohydride (Alfa Aesar, 1.590 g, 0.042 mol), and potassium hydroxide (Sigma-Aldrich, 4.250 g, 0.075mol) were suspended in 500 mL of ethanol. To the above mixture, 2,6-pyridinedimethanethiol (**C**) (1.440 g, 0.084 mol) and 1,3-bis(bromomethyl)benzene (Sigma-Aldrich, 2.220 g, 0.084 mol) in a mixture of 60 mL benzene and 60 mL ethanol was added dropwise while stirring under reflux for 5 hours. The reaction mixture was further stirred for 1 hour. The solvent was then removed under vacuum to give a white powder. The solid was dissolved in 30 mL of deionized water and extracted into dichloromethane (3×100 mL). The organic layer was then separated, dried with anhydrous magnesium sulfate, and then the solvent was removed under reduced pressure rotary evaporation. The white solid was a combination of dimer and oligomer. The desired dimer was purified by flash chromatography (20% EtOAc/hexanes).

Yield: 1.46 g, 64 %.

$^{1}$H NMR ($CD_2Cl_2$, 500 MHz), δ (ppm): 7.21 (t, 3H, Ar), 7.16 (dd, 1H, Ar), 6.87 (m, 5H, Ar), 3.92 (s, 4H, $CH_2$), 3.82 (s, 4H, $CH_2$).

$^{13}$C NMR ($CD_2Cl_2$, 125 MHz), δ (ppm): 158.23, 138.30, 137.12, 132.78, 128.75, 127.26, 121.72, 41.14, 39.22.

ESI-MS (*m/z*): 274.0728 (calcd for $[NCHS2H]^+$, $C_{15}H_{15}NS_2$, *m/z* 274.0672).

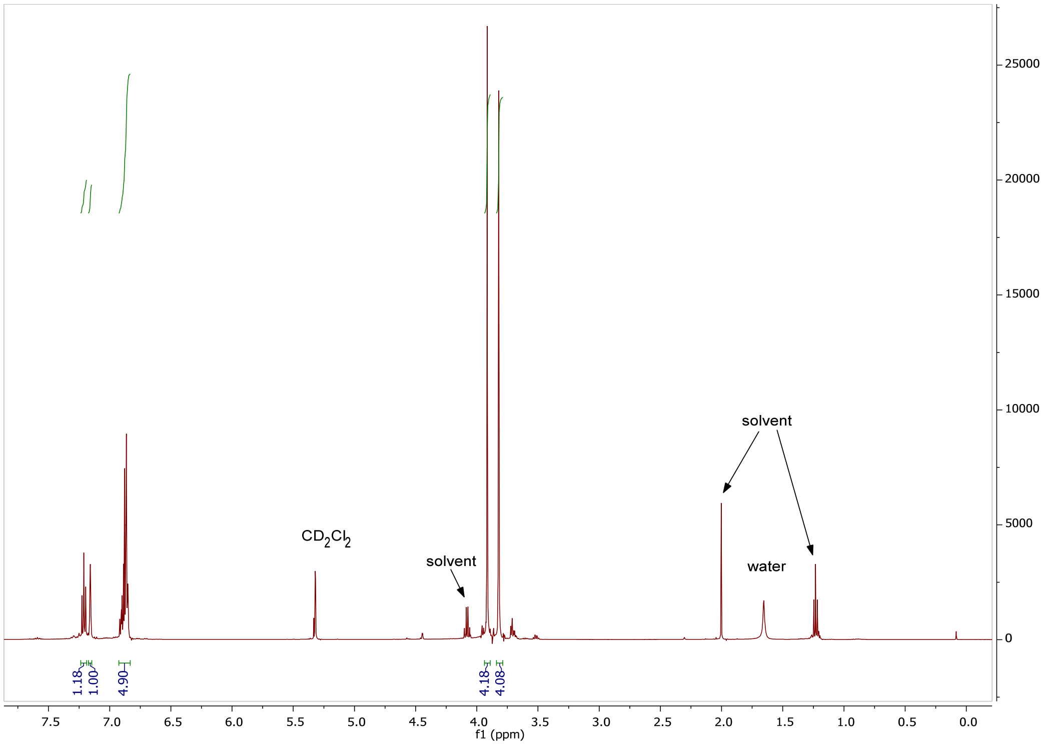


**Figure S1.** $^{1}$H NMR spectrum for NCHS2 ligand recorded in $CD_2Cl_2$.

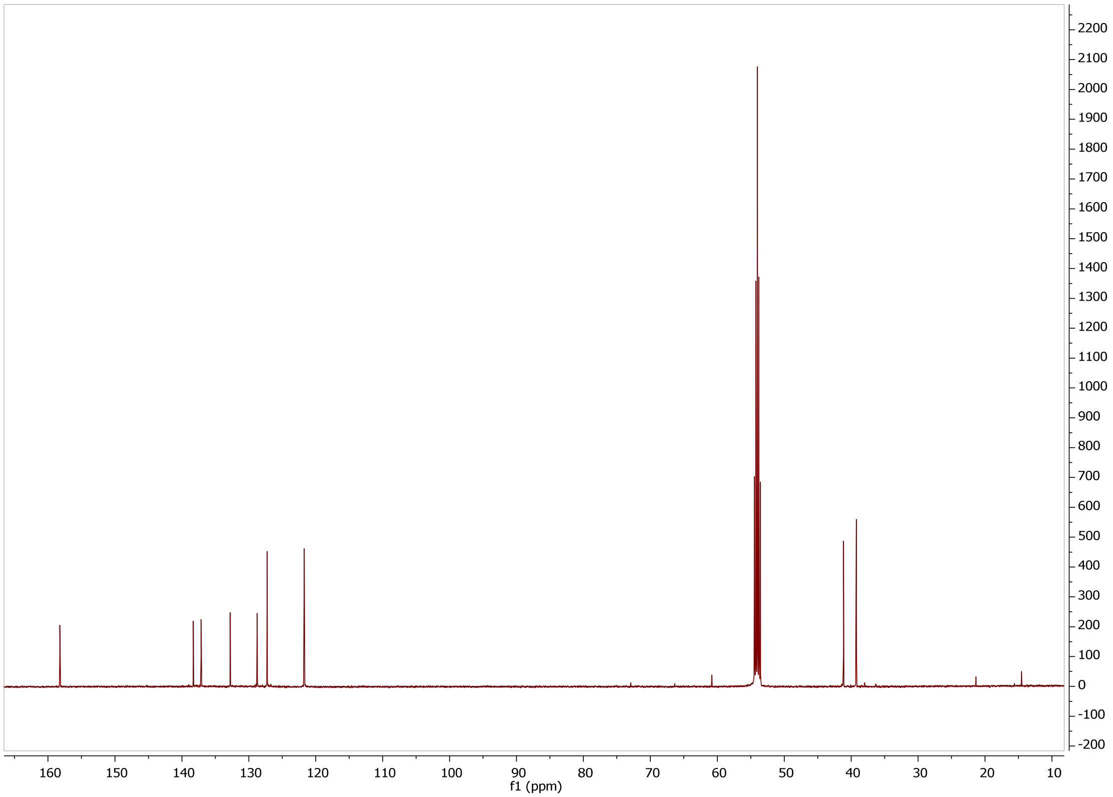


**Figure S2.** $^{13}$C NMR spectrum for NCHS2 ligand recorded in $CD_2Cl_2$.

**Preparation of NCBrS2 ligand**

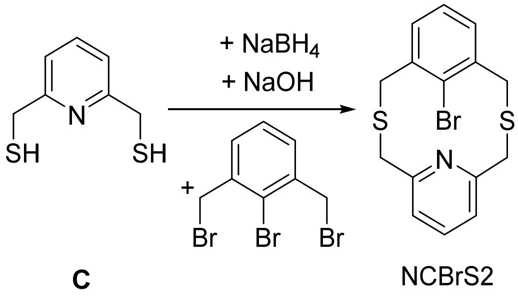


Compound **C** and 2-bromo-1,3-bis(bromomethyl)benzene were synthesized using modified published procedures.[2,4] In a 3-neck round bottom flask, sodium borohydride (Alfa Aesar, 0.56 g, 0.014 mol), and sodium hydroxide (Fisher, 1 g, 0.025 mol) were suspended in 500 mL 200 proof ethanol. To the above mixture, 2,6-pyridinedimethanethiol (**C**) (0.43 g, 0.0025 mol) and 2-bromo-1,3-bis(bromomethyl)benzene (0.86 g, 0.0025 mol) in a mixture of 60 mL acetonitrile and 60 mL ethanol was added dropwise while stirring under reflux for 5 hours. The reaction mixture was further stirred for 1 hour once the addition was completed. The solvent was then removed under vacuum to give a white powder, which was then dissolved in 30 mL deionized water and extracted with dichloromethane (3×100 mL). The organic layer was then separated, dried with anhydrous magnesium sulfate, and then the solvent was removed under reduced pressure rotary evaporation. The white solid was a combination of dimer and oligomer. The desired dimer product was purified by column chromatography on silica gel (9:1 hexane/ethyl acetate). NCBrS2 was collected as a white crystalline solid. Yield: 0.34 g, 38.0 %.

$^1$H NMR ($CDCl_3$, 500 MHz), δ (ppm): 7.27 (t, 1H, Ar), 7.97 (d, 2H, Ar), 6.92 (d, 2H, Ar), 6.76 (t, 1H, Ar), 4.70 (d, 2H, $CH_2$), 4.11 (d, 2H, $CH_2$), 3.77 (dd, 4H, $CH_2$), 3.65 (dd, 4H, $CH_2$).

$^{13}$C NMR ($CDCl_3$, 125 MHz), δ (ppm): 156.52, 136.84, 135.51, 129.09, 127.32, 125.82, 120.04, 37.58, 36.71, 28.68.

ESI-MS (*m/z*): 351.9830 (calcd for $[NCBrS2H]^+$, $C_{15}H_{15}BrNS_2$, *m/z* 351.9872).

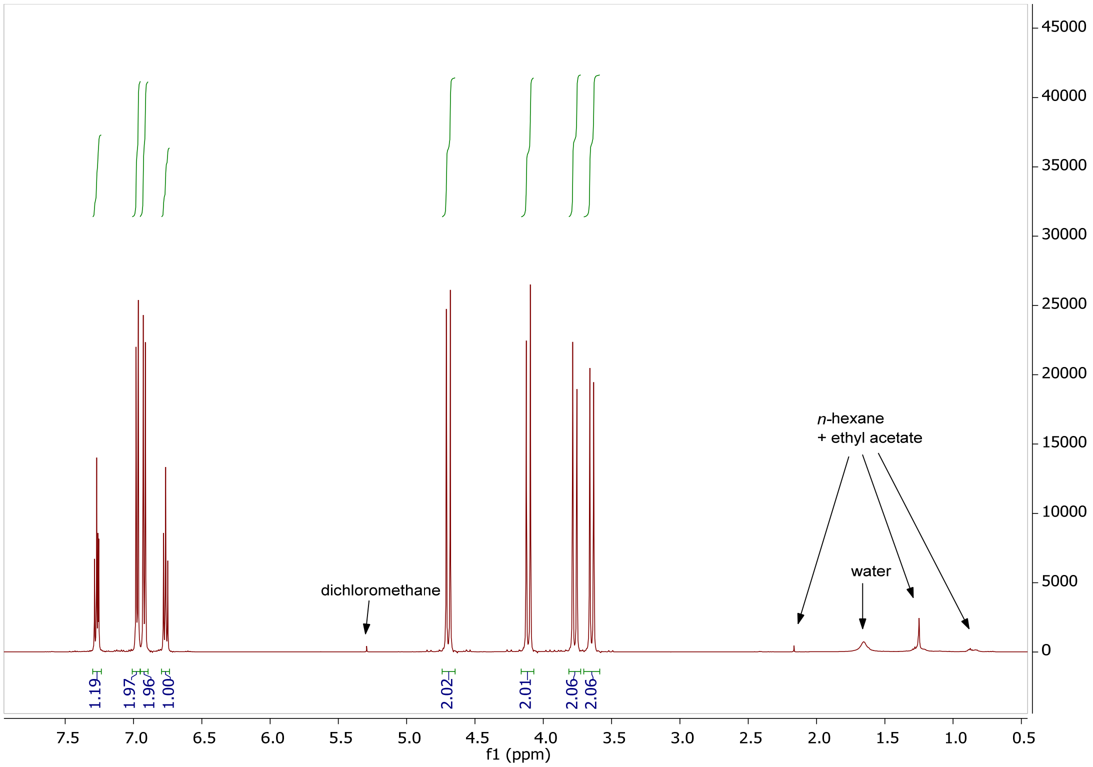


**Figure S3.** $^{1}H$ NMR spectrum for NCBrS2 recorded in $CDCl_3$.

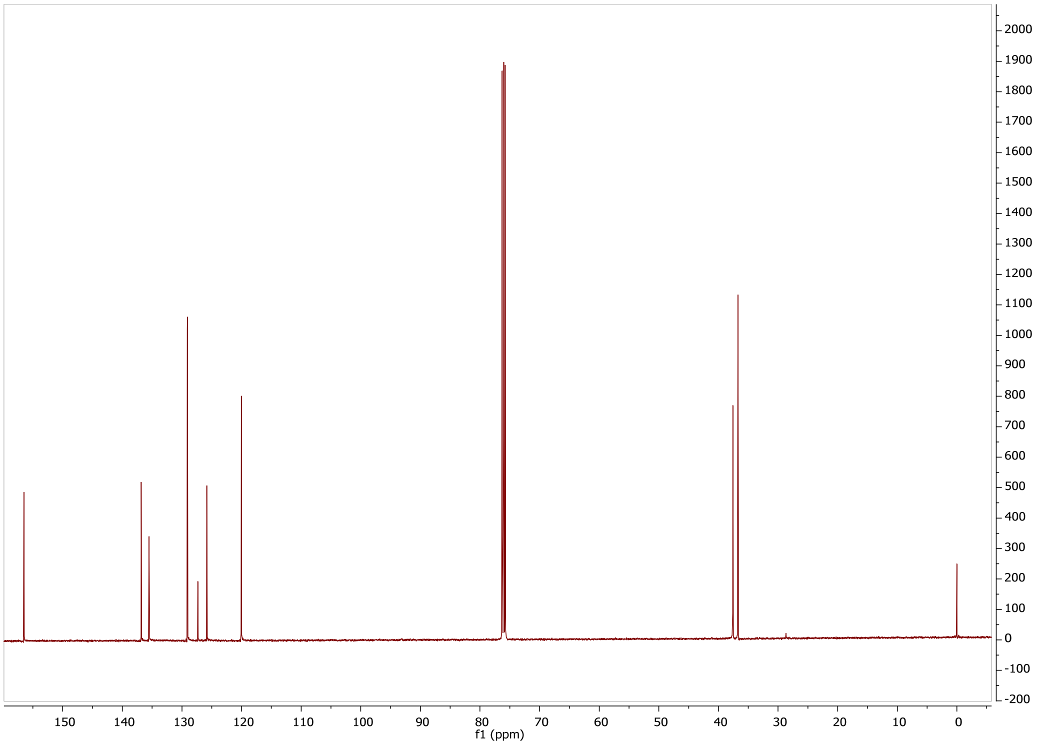


**Figure S4.** $^{13}C$ NMR spectrum for NCBrS2 recorded in $CDCl_3$.

**NMR Studies of (NCHS2)NiOTf$_2$**

(NCHS2)NiOTf$_2$ shows a distinct NMR spectrum when dissolved in $CD_3CN$. We propose that the complex has a square-planar structure with a SNS pincer and an acetonitrile making up the square plane. This would conform to a S=0 ground state.

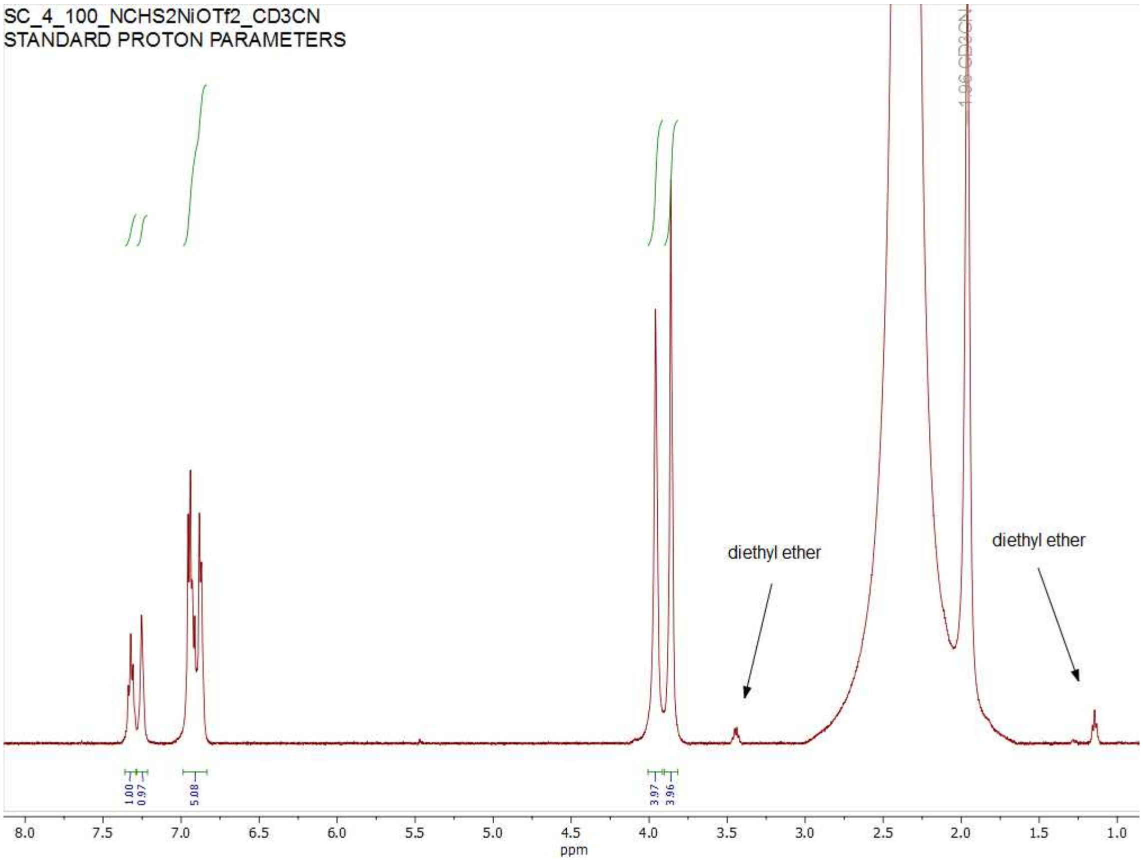


**Figure S5.** $^1$H NMR spectrum of **1** recorded in $CD_3CN$.

In order to further interrogate the dynamics of the complex in solution, we performed variable temperature NMR experiments on an $CD_3CN$ solution of **1**. We noticed a broadening of one of the peaks corresponding to the methylene protons as the temperature was lowered from room temperature to -25 °C. This suggests that at lower temperatures, the set of the methylene protons making up the square plane becomes more rigid, and the singlet broadens. The broadening is far more pronounced than the methylene protons adjacent to the phenyl, which remain dynamic even at low temperatures. The freezing point of $CD_3CN$ is -35 °C, which prevented us from conducting experiments at lower temperatures.

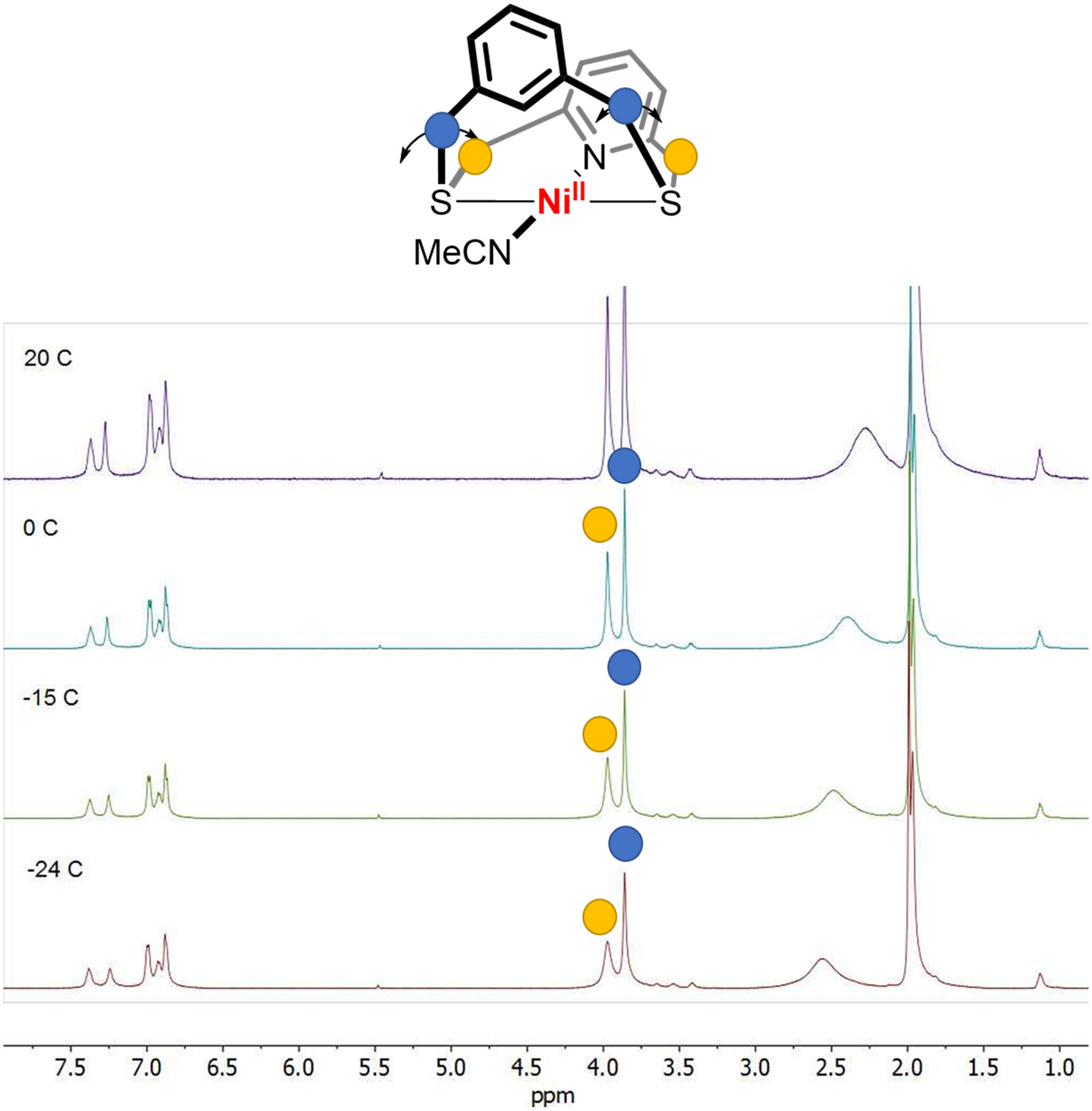


**Figure S6.** Variable temperature NMR spectra of **1** recorded in $CD_3CN$ – at temperatures of 20 ºC, 0 ºC, -15 ºC and -24 ºC.

Such molecular rearrangement, which makes the methylene protons inequivalent was also seen by VT-NMR for $[(N2S2)Pd^{II}(MeCN)_2]^{2+}$ and $[(N2S2)Pt^{II}(MeCN)_2]^{2+}$ by our group.[5]

**Discussion on the nature of 1 in solution:**

Experimental evidence suggests that **1** exists as a mixture of diamagnetic and paramagnetic species in acetonitrile solution. The complex has a well-defined NMR in the diamagnetic region, which when integrated against an internal standard of 1, 3, 5 trimethoxybenzene (47 mM, peaks

at 6.27 and 3.82 ppm), corresponds to ~60% of the species in solution (47 mM).

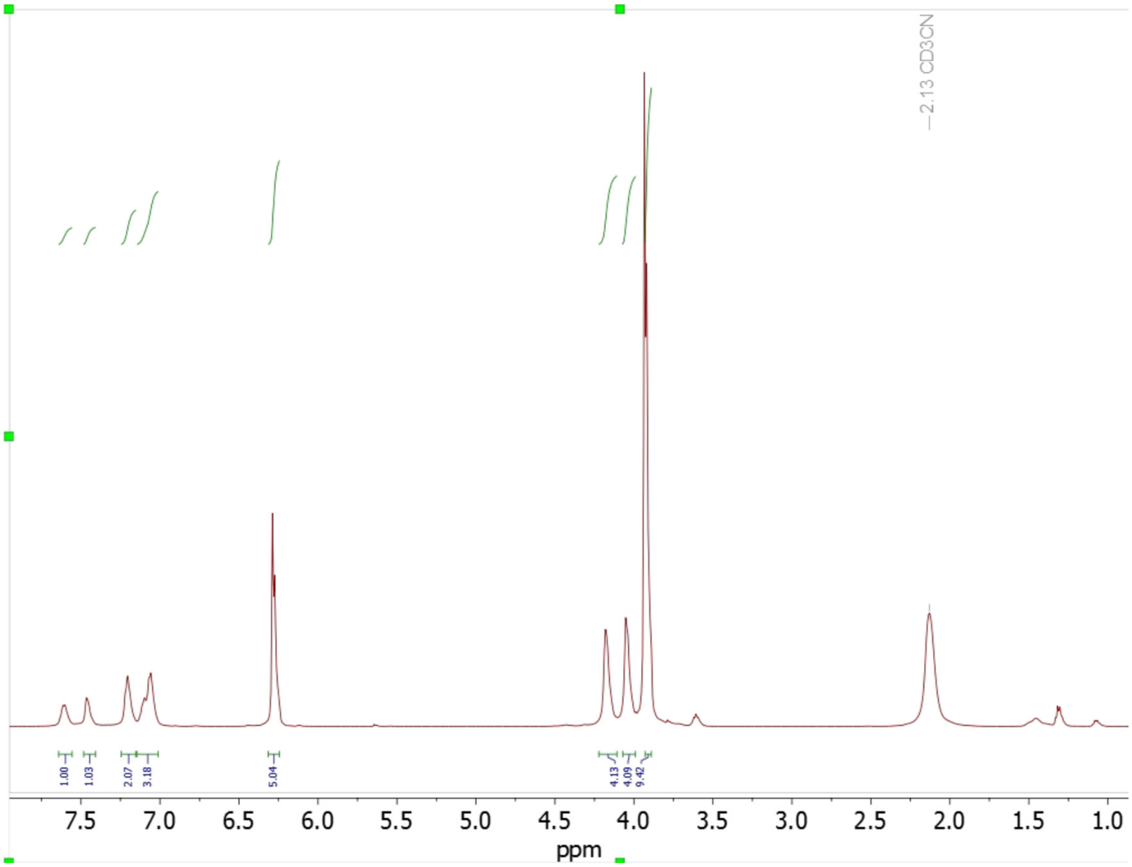


**Figure S7.** NMR of **1** with added internal standard (TMB).

Evan's Method gives a solution magnetic moment of 1.3 B.M. This corresponds to ~0.7 unpaired electrons. Considering two possible spin states, ~60% population of the S = 0 state and 40% population of the S = 1 state gives 0.8 unpaired electrons. Thus, we propose that the following species form when **1** in dissolved in acetonitrile.

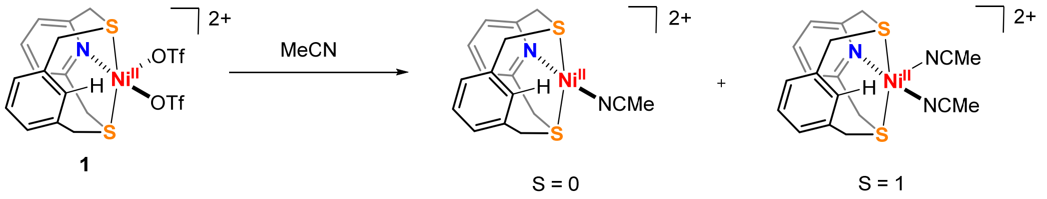

**Preparation of NCDS2**

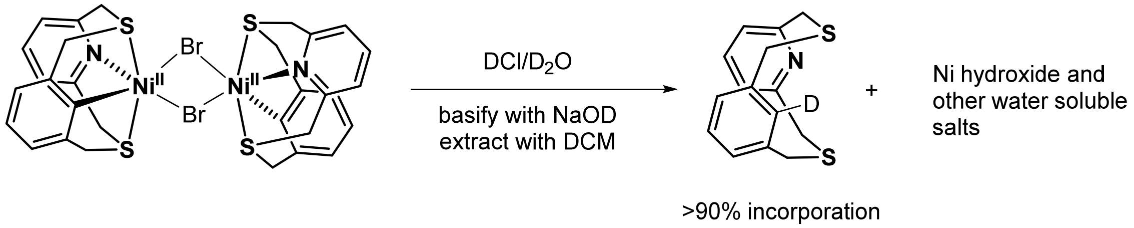


An acetonitrile solution of [(NCS2)Ni($\mu$-Br)]$_2$ was treated with 2 mL 20% DCl/$D_2O$ and stirred for 20 minutes in a scintillation vial in a $N_2$-filled glovebox. It was then sealed and brought out of the glovebox and heated at 60 ºC for 2 hours. The aqueous layer turned green indicating the formation of $NiCl_2$ salts. The solution was diluted with $D_2O$ and basified with NaOH. The organic layer was extracted in dichloromethane, dried over $MgSO_4$, and dried *in vacuo* to yield the ligand with deuterium in the *ipso* position. The deuterium incorporation was greater than 90% as indicated by using the *para* hydrogen triplet as an internal standard. ESI-MS (*m/z*): 275.0790 (calcd for $[NCDS2H]^+$, $C_{15}H_{15}DNS_2$, *m/z* 275.0787).

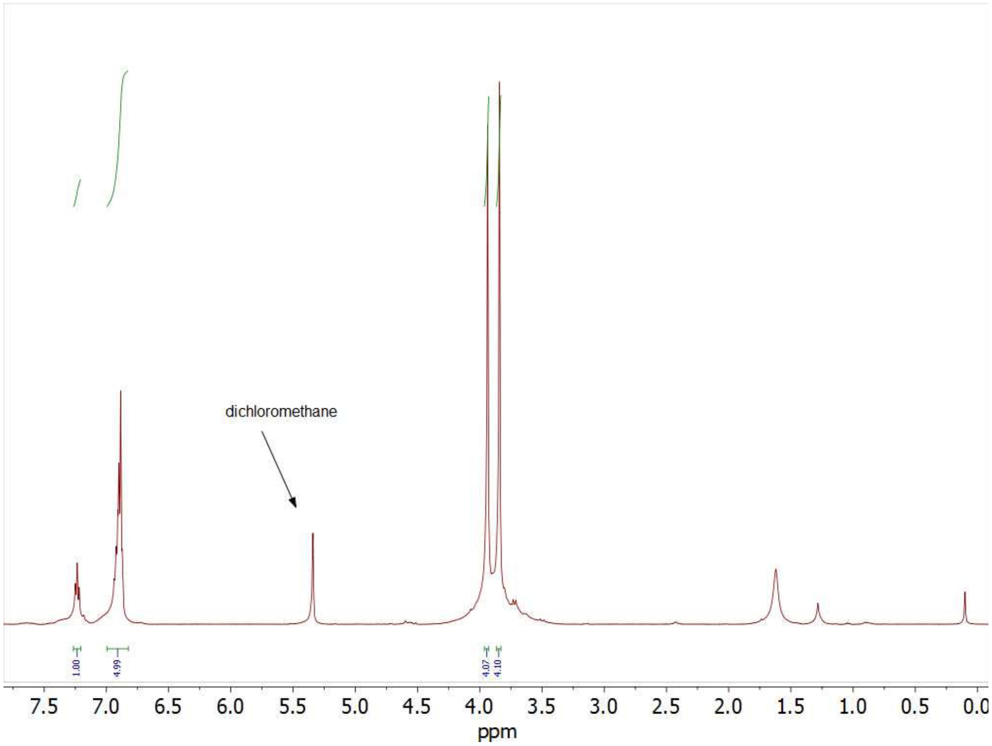


**Figure S8.** $^1$H NMR spectrum of NCDS2 recorded in $CD_2Cl_2$

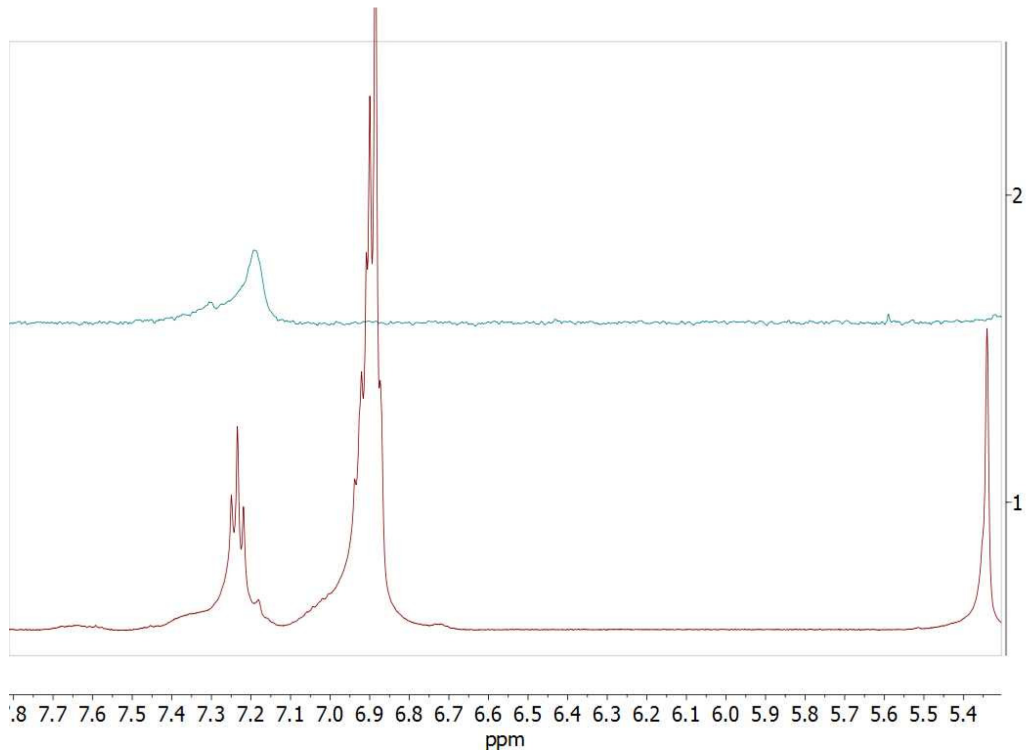


**Figure S9.** Stacked deuterium (top) and $^{1}$H NMR (bottom) spectra of NCDS2 recorded in $CD_2Cl_2$.

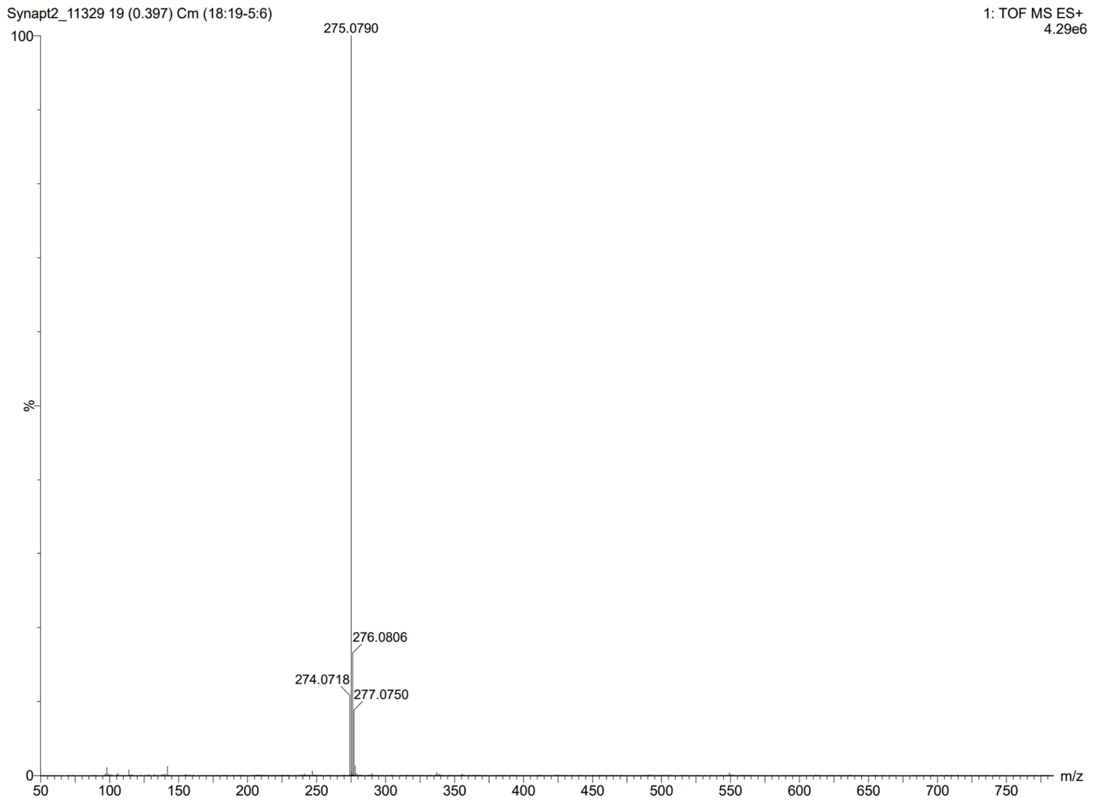


**Figure S9.** HR ESI-MS of NCDS2.

## H/D exchange in (NCDS2)$NiOTf_2$

In order to probe C-D(H) activation at $Ni^{II}$, a 1:1 $CD_3CN$:$CD_2Cl_2$ solution of NCDS2 and $NiOTf_2$ was heated at 70 °C and room temperature in a J-Young tube. The reaction was monitored after certain time periods as indicated in Fig. S10. The *ipso* C-D bond is shown to slowly exchange with adventitious protons over the course of 3 days to furnish the C-H bond. This indicates C-D(H) activation occurs with a thermal driving force in solution.

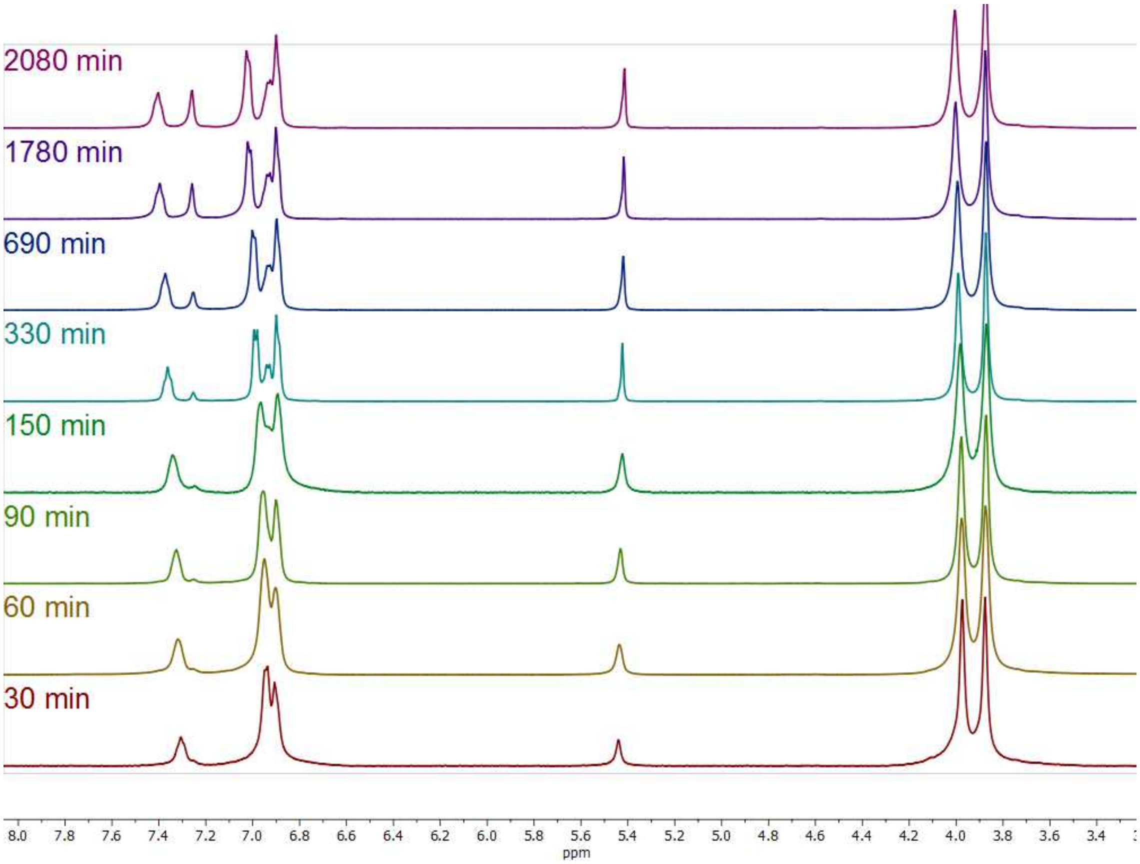


**Figure S10.** Stacked $^1$H NMR spectra of NCDS2 + $NiOTf_2$ over ~2100 minutes, showing the growth of the singlet corresponding to the *ipso* proton. The stacked NMRs also show the formation of the complex as the methylene protos at 3.9 ppm become inequivalent as the reaction proceeds. In addition, the multiplet from the aromatic protons become more complex with the progression of the reaction, which suggests the formation of the complex.

In addition, we also wanted to explore the H/D exchange without a thermal driving force. While the exchange is much slower at room temperature, there is ~15% (NCHS2)$NiOTf_2$ that is being

formed in solution over 3 days, when we start with a mixture of NCDS2 + $NiOTf_2$.

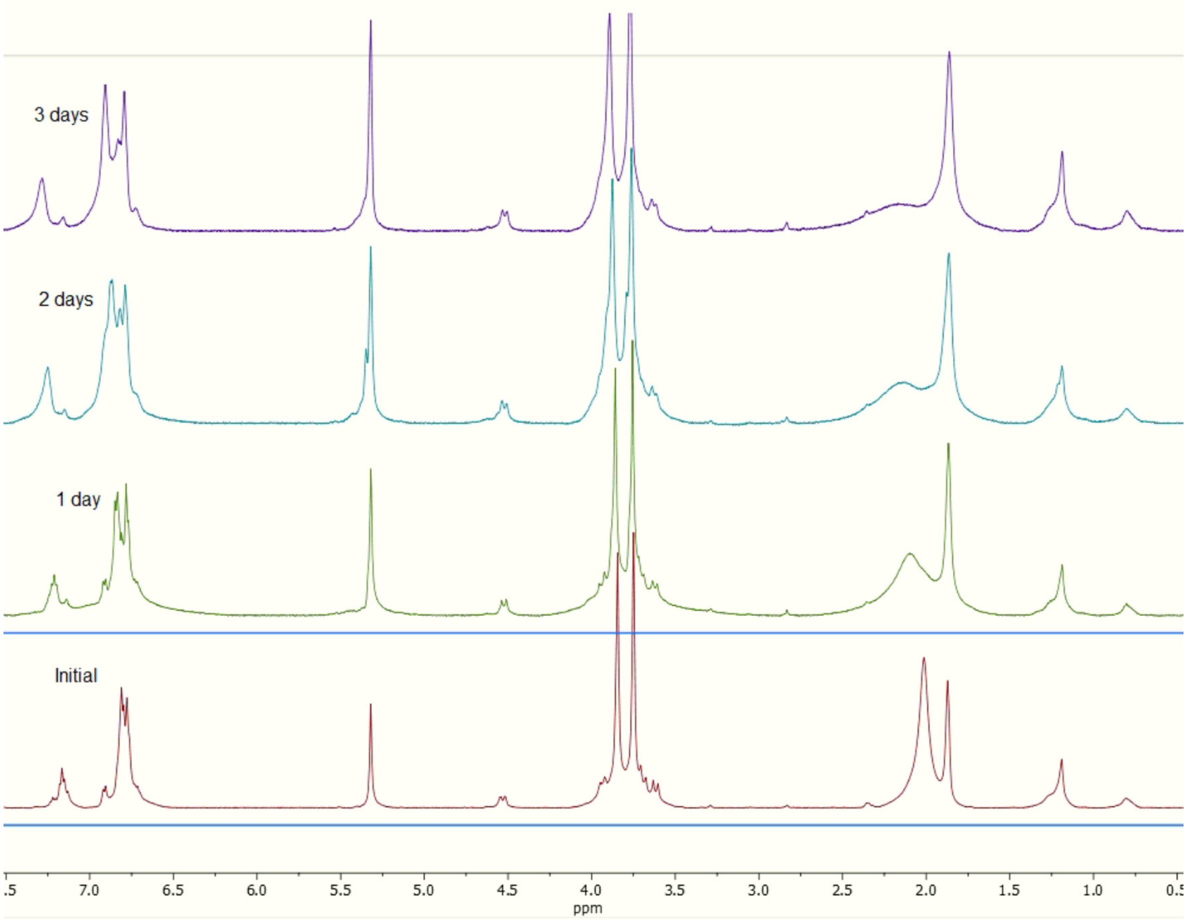


**Figure S11:** Stacked NMR spectra of NCDS2+$NiOTf_2$ in the absence of heating. The NMR suggests exchange of *ipso*-D with adventitious protons over the course of 3 days, albeit to a very low extent.

## MALDI-TOF Mass Spectrometry studies for 1 and 2-Br

### [(NCHS2)NiOTf$_2$], 1

The mass corresponding to the monocation [(NCHS2)NiOTf]$^+$ was found by MALDI. Calcd. for C16H15F3NNiO3S3: m/z 479.9520. Found m/z 479.9156.

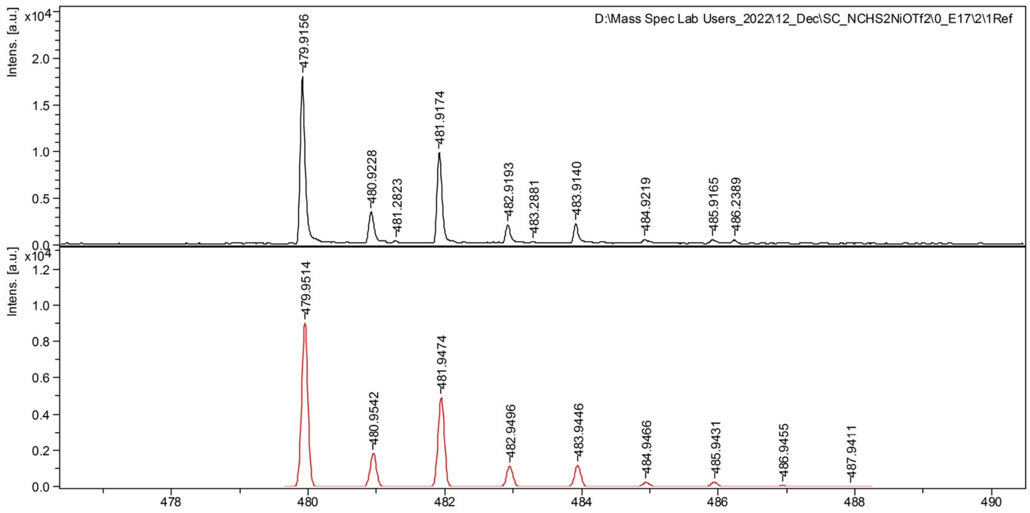


**Figure S12.** Experimental and predicted mass envelope pattern for [(NCHS2)NiOTf]$^+$.

### [(NCS2)Ni(μ-Br)]$_2$, 2-Br

The mass corresponding to the monocationic proton adduct of 2-Br was found in the MALDI MS. Calcd. for [C30H28Br2N2Ni2S4+H]: m/z 820.8267**,** found m/z = 820.8760

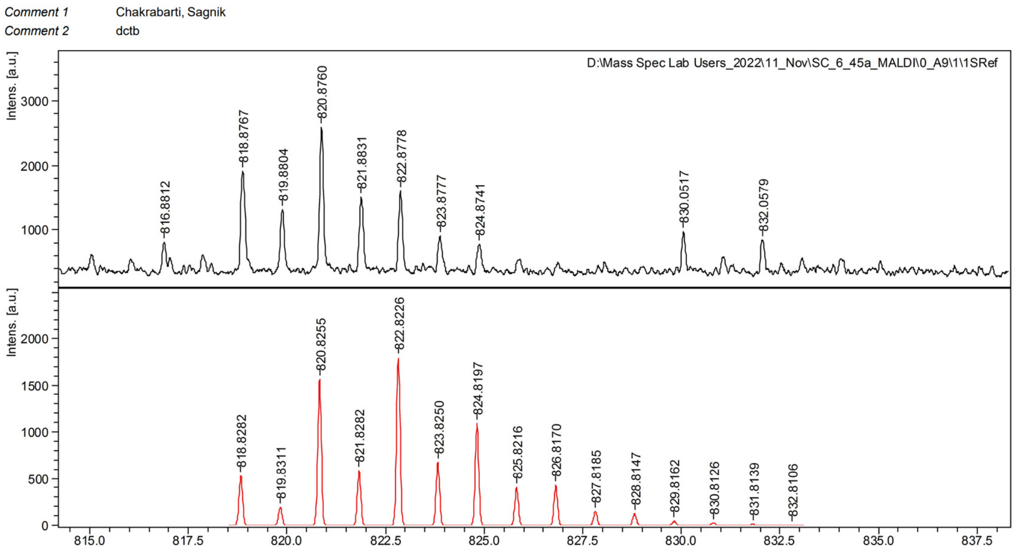


**Figure S13.** Experimental and predicted mass envelope patter for [(NCS2)NiBr]$_2$ + H$^+$.

## 3. Electrochemical studies

### Cyclic voltammograms (CVs) for bare glassy carbon (GC) electrode

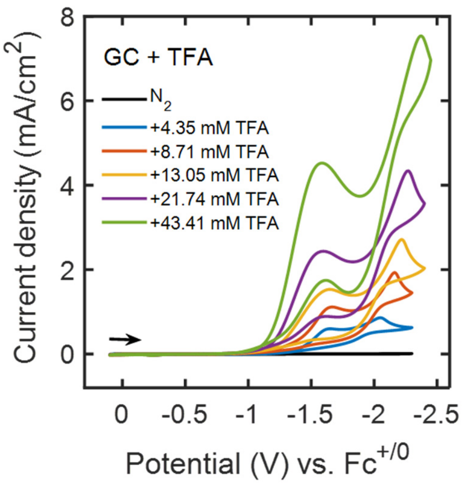


**Figure S14.** CVs recorded for bare GC electrode in $N_2$-saturated 0.1 M $TBAPF_6$ MeCN solution in the absence (black) and the presence of different concentrations of TFA (4.35 mM - 43.41 mM) as shown in the legend. All CVs were recorded at 0.1 V/s scan rate. The arrow shown in the figure indicates the direction of the CV recorded.

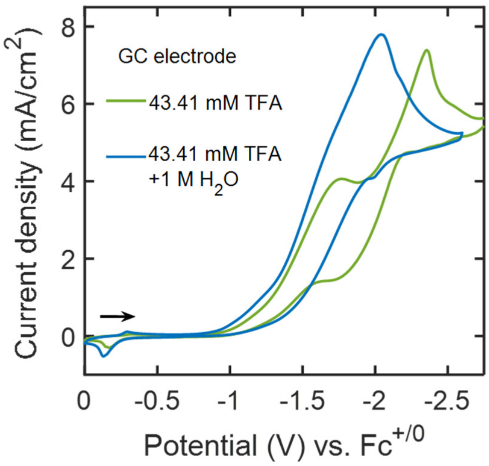


**Figure S15.** CVs recorded for bare GC electrode in $N_2$-saturated 0.1 M $TBAPF_6$ MeCN solution in the presence of 43.41 mM of TFA (green) and 43.41 mM TFA + 1 M $H_2O$ (blue). All CVs were recorded at 0.1 V/s scan rate. The arrow shown in the figure indicates the direction of the CV recorded.

**Cyclic voltammograms (CVs) for (NCHS2)Ni(OTf)$_2$ , 1(OTf)$_2$**

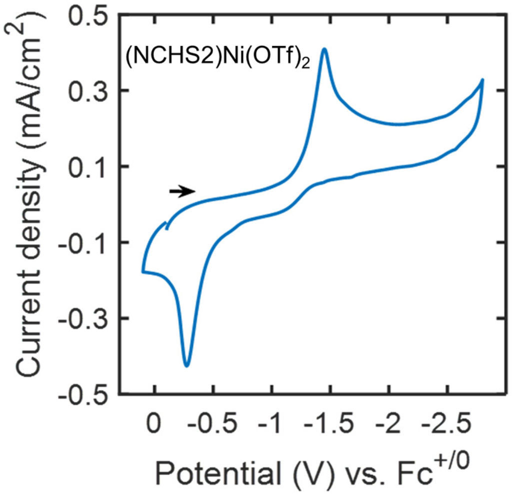


**Figure S16.** CV recorded for **1** in $N_2$-saturated 0.1 M $TBAPF_6$ MeCN solution at scan rate 0.1 V/s. The arrow shown in the figure indicates the direction of the scan.

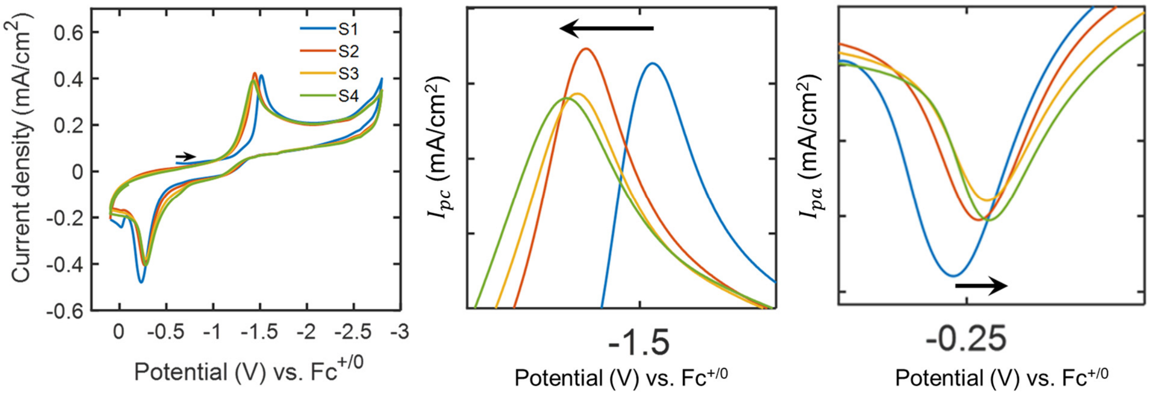


**Figure S17.** CVs recorded for **1** in $N_2$-saturated 0.1 M $TBAPF_6$ MeCN solution upon four repeating CV sweeps (S1-S4) at the scan rate of 0.1 V/s (Left). The movement of cathodic peaks (middle) and the anodic peaks (right) at $Ni^{II/I}$ redox couple are enlarged as observed for the left. Note: the cathodic- and anodic peaks moved toward higher and lower potential, respectively, during repeating CV cycles for **1**.

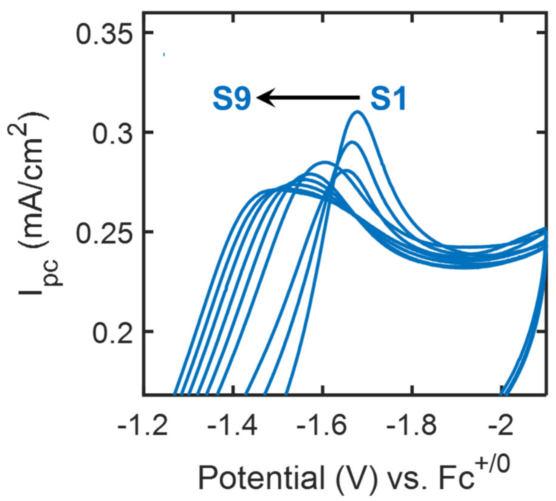


**Figure S18.** The shift of cathodic peaks at $Ni^{II/I}$ reduction wave for **1** in $N_2$-saturated 0.1 M $TBAPF_6$ MeCN upon nine repeating CV cycles (S1-S9). Scan rate = 0.1 V/s. $I_{pc}$ = peak current densities at the reductive waves. The arrow shown in the figure indicates the movement of peak positions during the repeating CV sweeps.

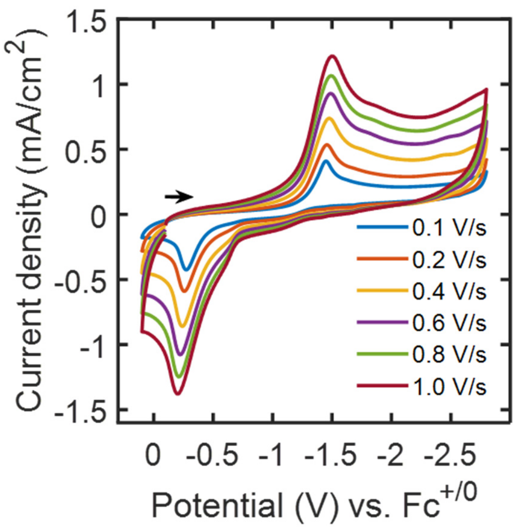


**Figure S19.** CVs recorded for **1** in $N_2$-saturated 0.1 M $TBAPF_6$ MeCN solution at different scan rates (0.1 V/s – 1.0 V/s). The arrow shown in the figure indicates the direction of the scan.

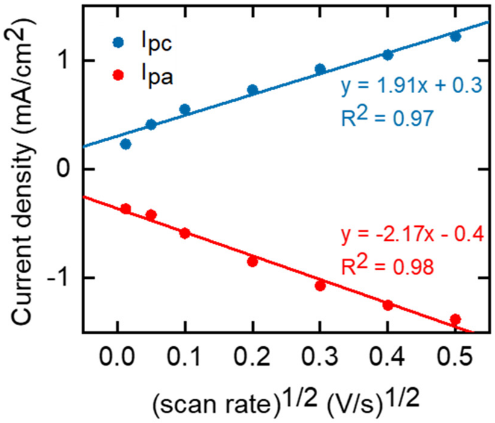


**Figure S20.** Peak current densities obtained at the $Ni^{II/I}$ reduction wave for **1** in $N_2$-saturated 0.1 M $TBAPF_6$ MeCN solution at different scan rates (0.05 V/s – 1.0 V/s) are plotted versus square root of the scan rates. $I_{pc}$ and $I_{pa}$ denote reductive and oxidative peak current densities, respectively.

**Peak Shift Analysis**

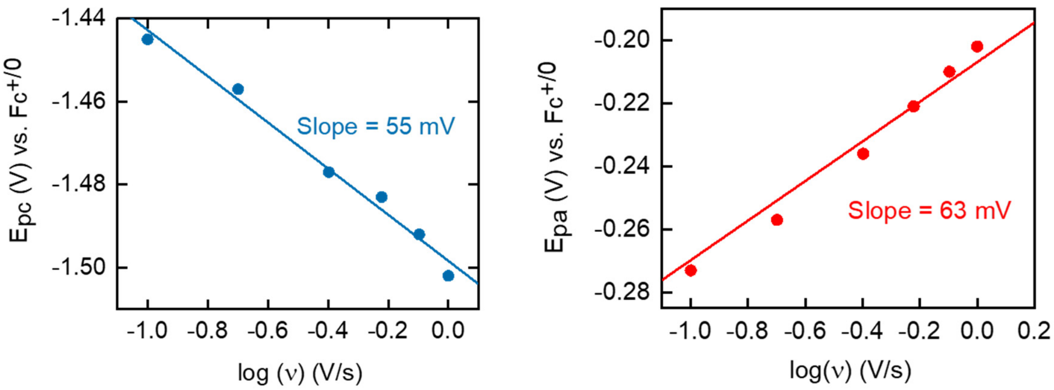


**Figure S21.** Cathodic, $E_{pc}$ (left) and anodic, $E_{pa}$ (right) peak potentials obtained for **1** in $N_2$-saturated 0.1 M $TBAPF_6$ MeCN solution at different scan rates (0.1 V/s – 1.0 V/s) are plotted versus logarithm of scan rate (V/s). $R^2$ value for both the linear fits is greater than 0.97.

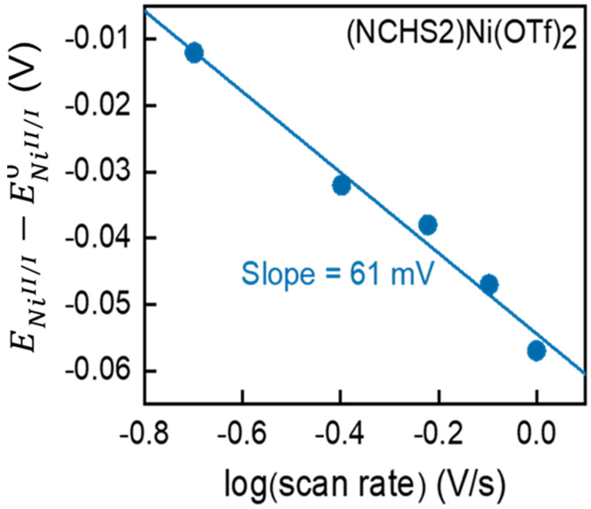


**Figure S22.** The peak potentials for the $Ni^{II/I}$ reduction ($E_{Ni^{II/I}}$) at different scan rates shifted linearly with a slope of 61 mV per decade, which is much larger than the characteristic slope of a typical Nernstian EC mechanism. This suggests a substantial structural rearrangement following a slow electron transfer.

For our initial studies, we chose to perform electrocatalytic HER using AcOH in MeCN. **1** exhibited current increase at potentials more negative than –2.0 V (Figure S24), yet the quasi-plateau currents at higher AcOH concentrations appeared at only 140 mV more positive potential than that of the direct proton reduction by the GC electrode (Figure S25), and thus a stronger acid, trifluoroacetic acid (TFA), was used in subsequent studies. We show the data for electrochemical HER using both AcOH and TFA.

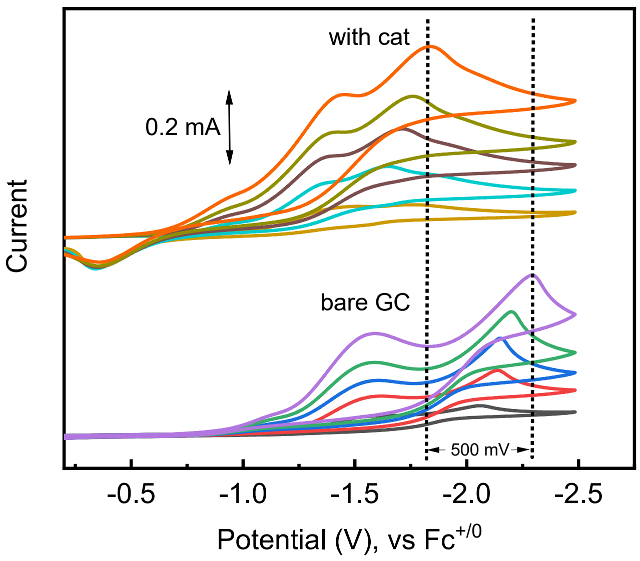

**Figure S23.** Comparative CVs collected for glassy carbon ('bare GC') and **1** ('with cat') in $N_2$-saturated 0.1 M $TBAPF_6$/MeCN and increasing TFA concentrations.

The addition of $H_2O$ is common for HER studies as $H_2O$ can improve the proton supply and thus can enhance the reaction kinetics without compromising the thermodynamic limitations. The addition of $H_2O$ at different concentrations (0.8-2 M), revealed plateau current densities for **1** at potentials lower than –1.5 V in presence of 0.043 M TFA (Figure S23). Similar quasi-plateau current densities were also observed in presence of 0.058 M AcOH upon addition of $H_2O$, but at potentials lower than –2 V. Overall, these results strongly suggest that **1** is an efficient electrocatalyst to perform HER at low acid concentration using a moderate to weak acid in wet MeCN.

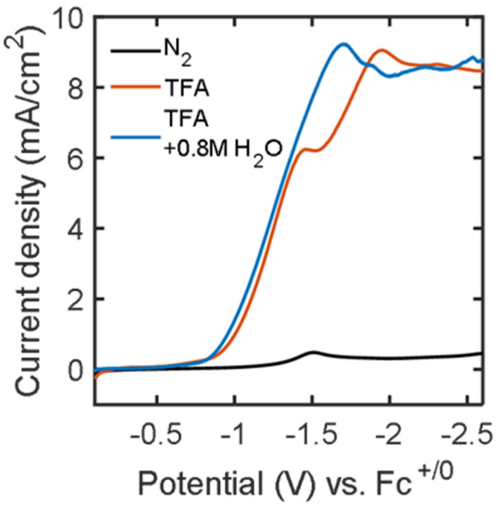


**Figure S24.** CVs recorded for **1** in $N_2$-saturated 0.1 M $TBAPF_6$ MeCN solution in absence of TFA (back), presence of 0.043 M TFA (orange), and presence of 0.043 M TFA + 0.8 M $H_2O$ (blue).

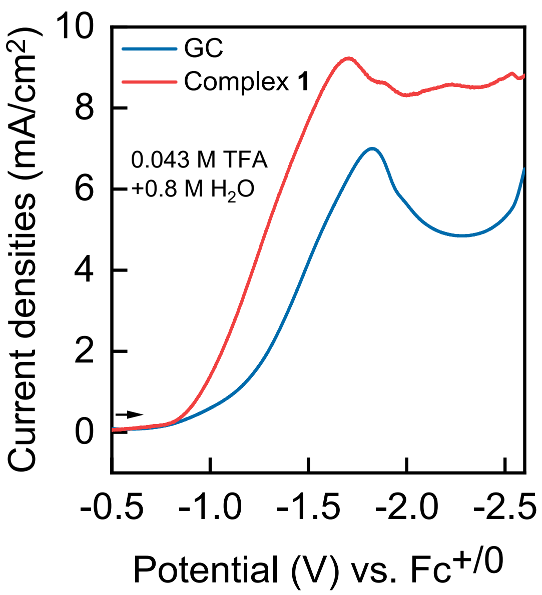


**Figure S25.** CVs recorded for **1** (red) and bare GC (blue) in $N_2$-saturated 0.1 M $TBAPF_6$ MeCN solution in presence of 0.043 M TFA + 0.8 M $H_2O$.

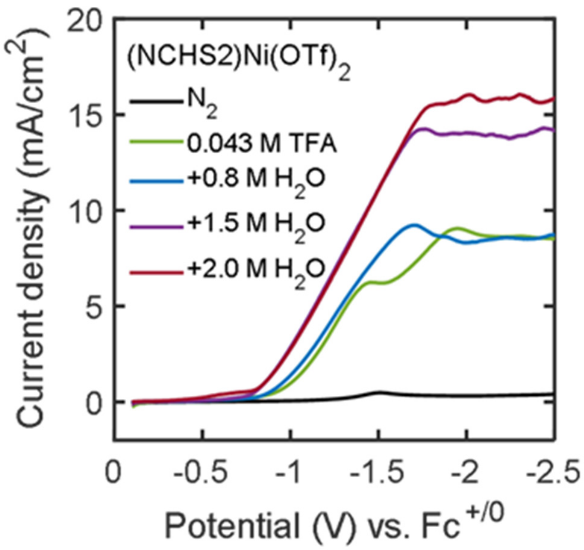


**Figure S26.** CVs recorded for **1** in $N_2$-saturated 0.1 M $TBAPF_6$ MeCN in the absence of TFA (black), and 0.043 M TFA + varying concentrations of $H_2O$. Scan rate = 0.1 V/s. Only forward scans are shown for clarity.

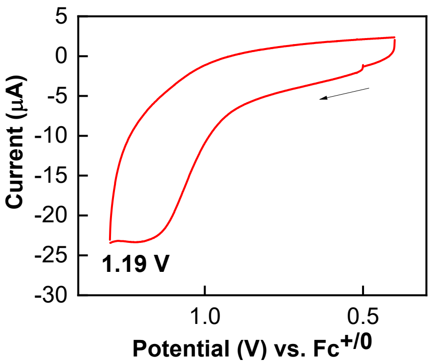


**Figure S27.** CVs recorded for **1** in $N_2$-saturated 0.1 M $TBAPF_6$ MeCN within the electrochemical window between 0.4 V and 1.4 V vs. $Fc^{+/0}$. Scan rate = 0.1 V/s.

## Cyclic voltammograms (CVs) for [(NCS2)Ni(MeCN)$_2$](OTf), 2(OTf)

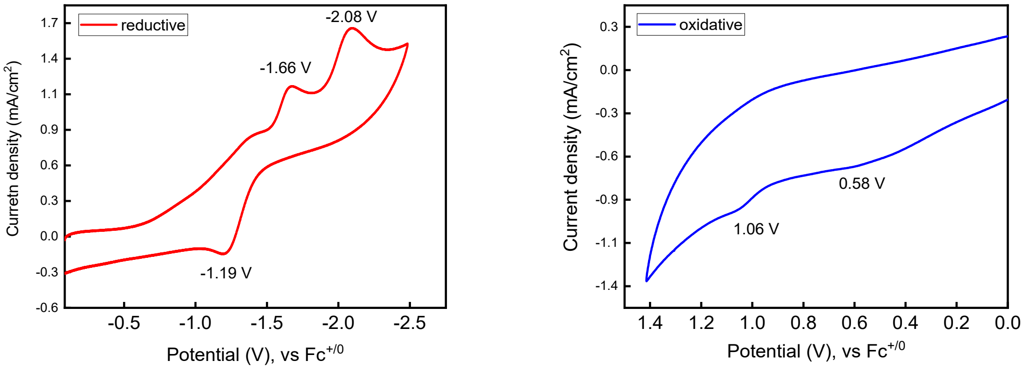


**Figure S28.** CVs recorded for **2** in $N_2$-saturated 0.1 M $TBAPF_6$/MeCN. Oxidative and reductive scans are shown separately.

(a)

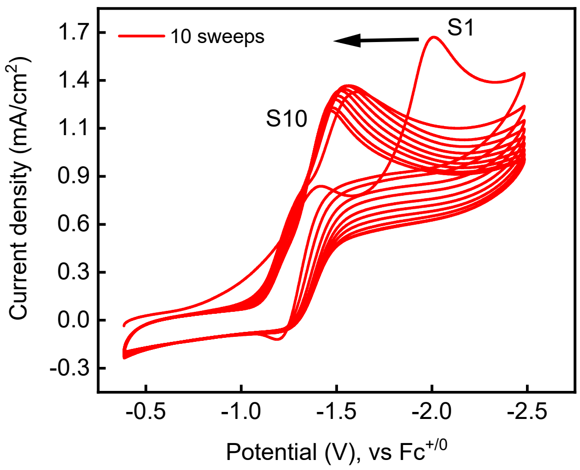


(b)

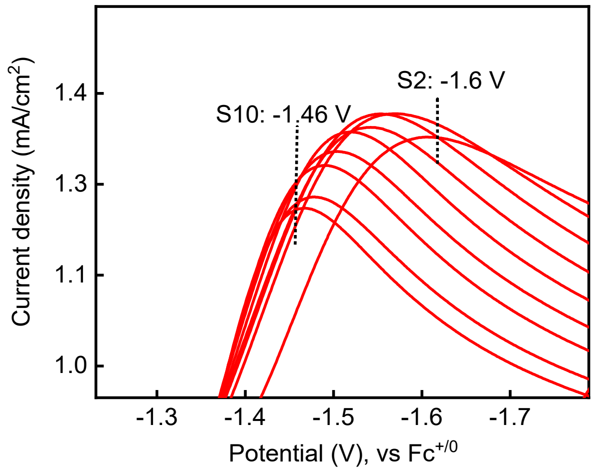


**Figure S29.** Multiple scans of reductive CVs recorded for **2** in $N_2$-saturated 0.1 M $TBAPF_6$/MeCN. It is noteworthy that after the first scan, the CVs resemble the reductive CVs of **1** in MeCN. While the exact nature of the voltammogram can be difficult to assess, we propose an irreversible structural change on reduction, that is, the C-H bond is cleaved on reduction which would give back complex **1**. The CVs seen in the subsequent scans are due to complex **1**.

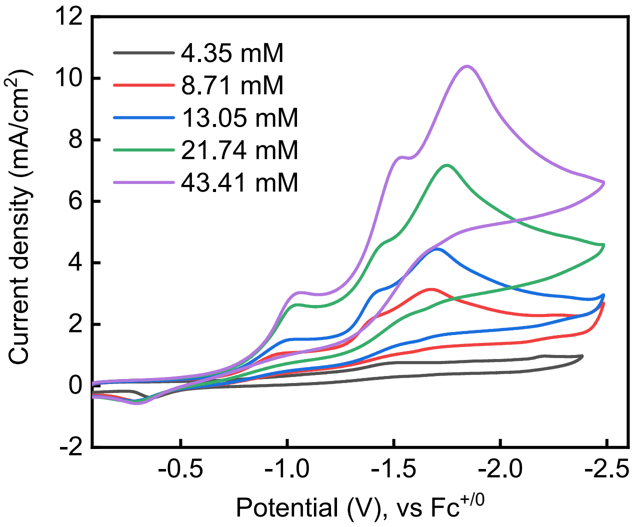


**Figure S30.** CVs recorded for **2** in $N_2$-saturated 0.1 M $TBAPF_6$/MeCN solution in the presence of different concentrations of TFA (4.53 mM – 43.41 mM) as shown in the legend. All CVs were recorded at 0.1 V/s scan rate.

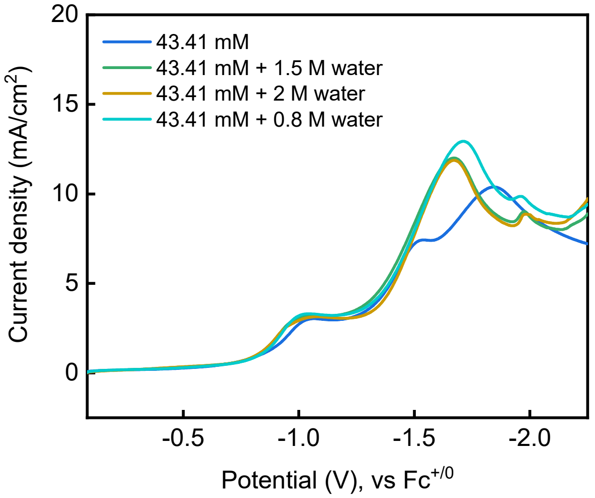


**Figure S31.** CVs recorded for **2** in $N_2$-saturated 0.1 M $TBAPF_6$ MeCN with 0.043 M TFA + varying concentrations of $H_2O$. Scan rate = 0.1 V/s.

**Table S1.** Cathodic peak current densities for **1** and **2** in the presence of TFA

| Conc. of TFA | Peak current densities for **1** ($mA/cm^2$) | Peak current densities for **2** ($mA/cm^2$) |
|---|---|---|
| 4.35 mM | 1.19, 1.66 | 0.73, 0.94 |
| 8.71 mM | 1.77, 2.36 | 2.07, 3.13 |
| 13.05 mM | 2.38, 3.16 | 3.03, 4.45 |
| 21.74 mM | 3.71, 5.33 | 4.59, 7.17 |
| 43.41 mM | 6.63, 9.89 | 7.42, 10.38 |

**Cyclic Voltammograms for [(NCS2)$Ni^{III}$(MeCN)][OTf]$_2$ ([2]$^+$)**

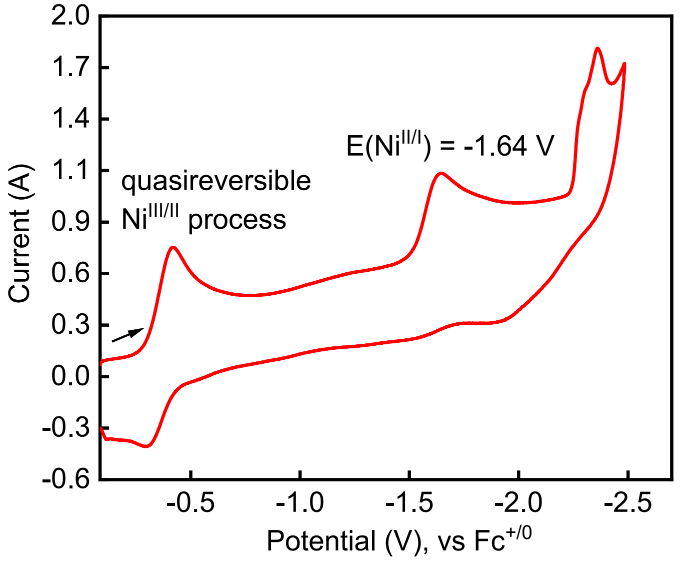


**Figure S32.** CV recorded for **[2]**$^+$ in $N_2$-saturated 0.1 M TBAPF$_6$ MeCN solution at scan rate 0.1 V/s. The arrow shown in the figure indicates the direction of the scan.

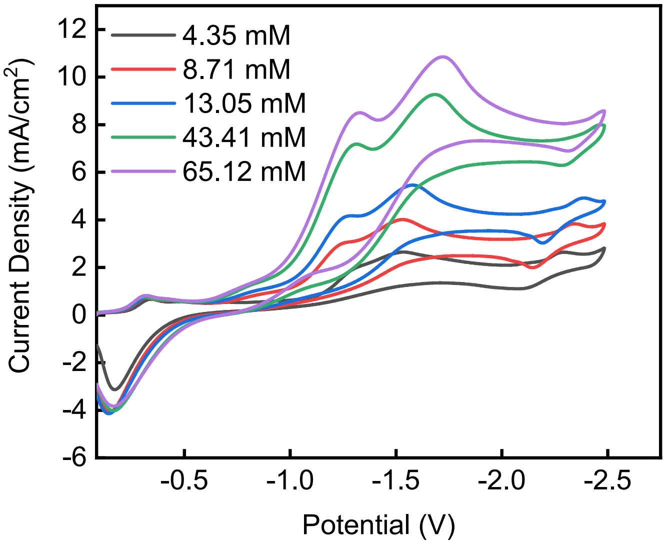


**Figure S33.** Titrations of TFA into 1 mM **[2]**$^+$ produced CVs with similar current densities as **1** and **2**, indicating it is also a viable catalyst for HER. The reductive peak at -0.3 V possibly indicates the reduction of $Ni^{III}$ to $Ni^{II}$ (the lowering of potential could be facilitated by pyridine protonation). The subsequent reductions are assigned as $Ni^{II/I}$ and $Ni^{III}$-H/$Ni^{II}$-H, both of which are active in catalysis according to the proposed mechanism.

**Cyclic Voltammograms for [(NCS2)Ni(μ-Br)]$_2$ (2-Br)**

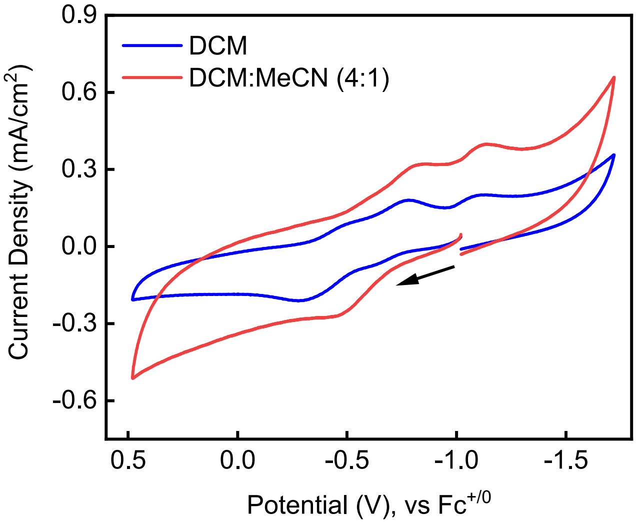


**Figure S34.** Oxidative CVs for **[2-Br]** in DCM and 4:1 DCM:MeCN. The CVs could not be recorded in pure acetonitrile because of solubility issues. Presumably, the dimer breaks apart

in solution, as in evident by the formation of two kinds of $Ni^{III}$ species. These putative $Ni^{II}$ species could be differently charged, which would cause differential solubility.

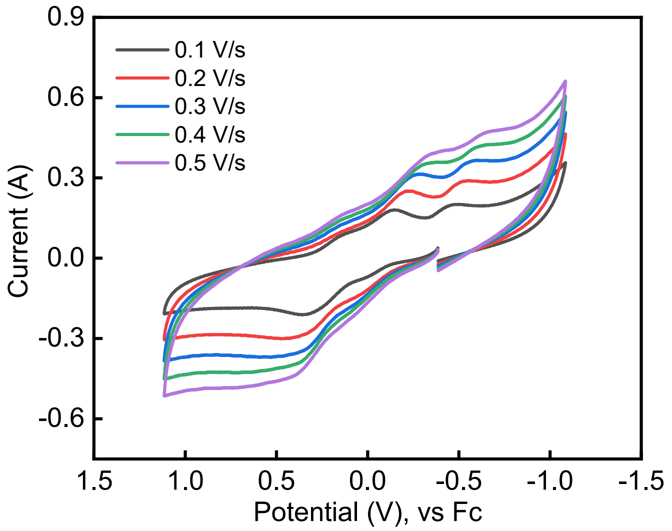


**Figure S35.** Scan rate dependent cyclic voltammograms of **2-Br** in a 0.1 M $TBAPF_6$/DCM solution.

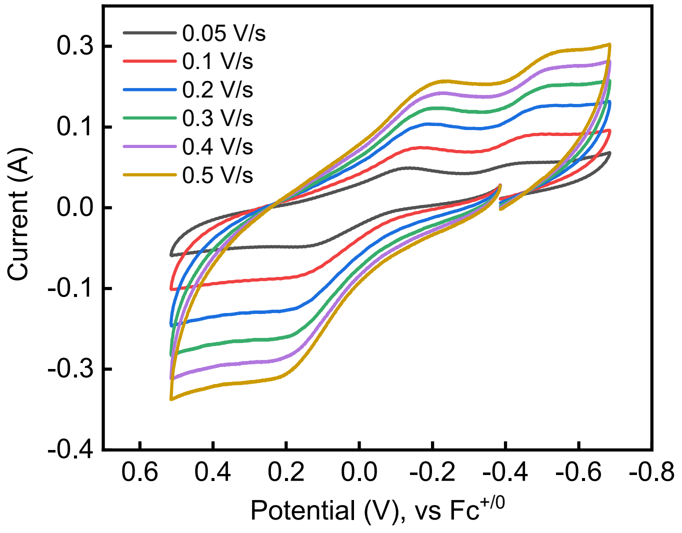


**Figure S36.** Scan rate dependent cyclic voltammograms of **2-Br** in 0.1 M $TBAPF_6$/1:4 MeCN:DCM solution.

**Cyclic Voltammograms for $[(N2S2)Ni(MeCN)_2][BF_4]_2$**

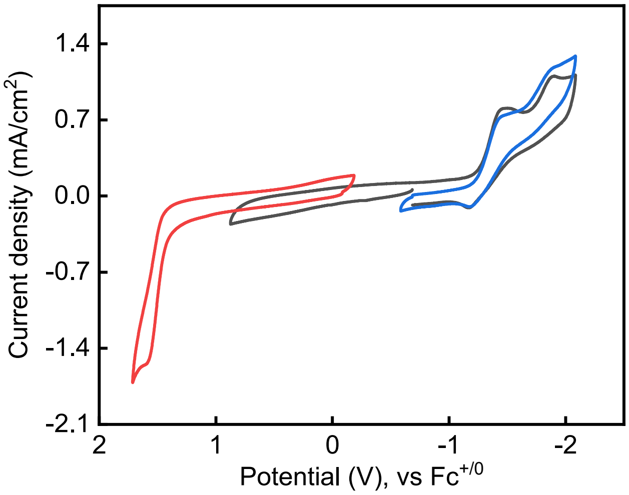


**Figure S37.** Small and wide range CVs of 1 mM $[(N2S2)Ni(MeCN)_2][BF_4]_2$ recorded in a $N_2$-saturated 0.1 M $TBAPF_6$/MeCN solution. Two reductive events are observed which have been assigned to $Ni^{II/I}$ and $Ni^{I/0}$ processes.

**Control experiments for checking the homogeneity of 1**

**Rinse Tests**

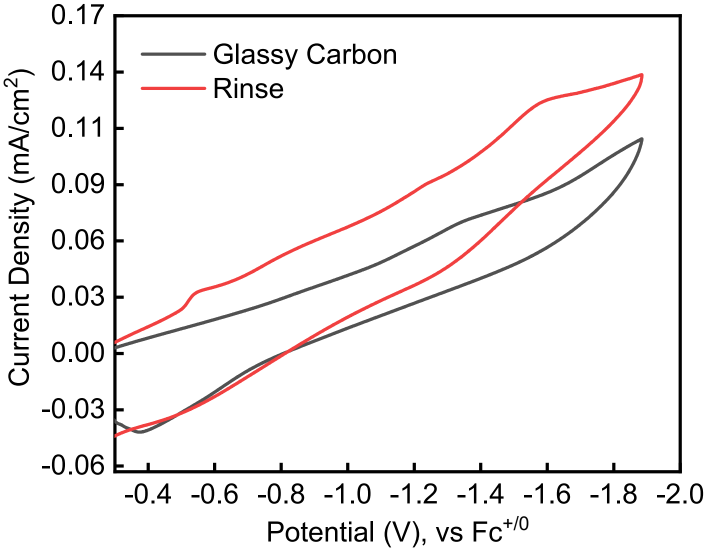


**Figure S38.** To check for the homogeneity of **1** in solution, in these experiments, the working electrode was dipped into the catalyst containing solution before being rinsed with excess MeCN and placed into a fresh solution of MeCN containing 0.1 M $TBAPF_6$. CVs were recorded before (black) and after (red) the rinse.

(a)

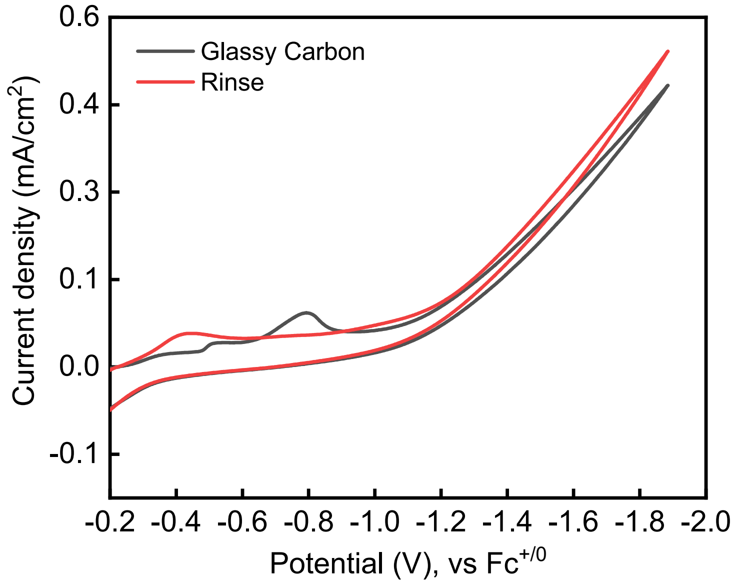

(b)

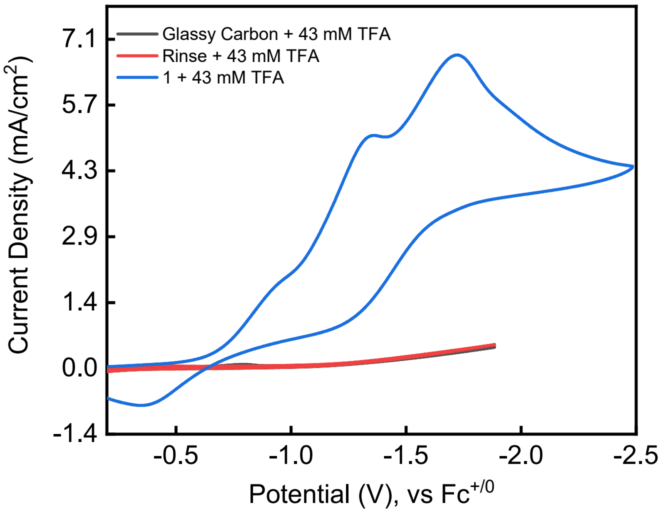


**Figure S39.** To check for the catalytic activity of any surface-adsorbed species, the working electrode was dipped into the catalyst containing solution before being rinsed with excess MeCN and placed into a fresh solution of MeCN containing 0.1 M $TBAPF_6$ and 43 mM TFA.[6] (a) CVs were recorded before (blue) and after (red) the rinse. (b) In comparison, the CV of a homogeneous solution of the catalyst in the presence of the same concentration of acid showed significantly enhanced currents. While we cannot rule out some surface adsorption of the catalyst, the minor changes in the voltammogram indicate that contribution of surface adsorbed species is minimal.

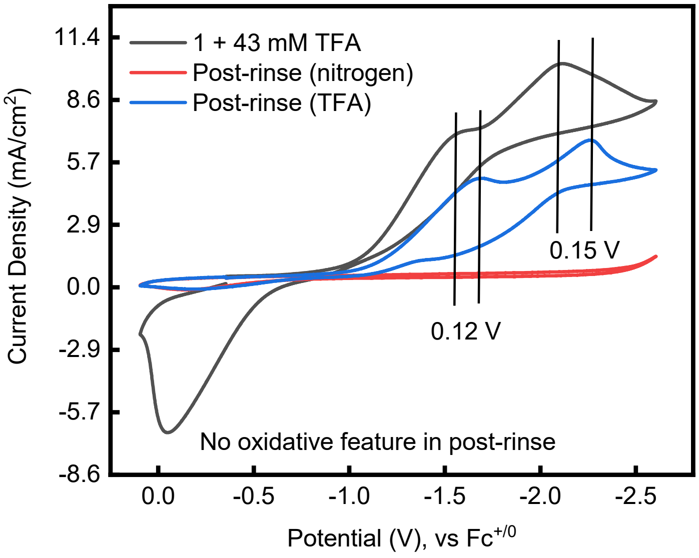

**Fig S40.** In addition to the experiments described above, a second rinse test was performed. A CV of **1** was recorded in the presence of 50 mM TFA (black). Then the same solution was electrolyzed for 1 hour at -1.7 V vs $Fc^{+/0}$. The glassy carbon electrode was then rinsed with acetone and MeCN and a CV was recorded with a $N_2$ purged MeCN solution (red), which showed no distinct Faradaic features, indicating that there is no strong adsorption to the surface. A CV was recorded with 50 mM TFA added (blue), and the voltammogram strongly resembles that of a bare glassy carbon electrode, with higher currents and $E_{pc1}$ and $E_{pc2}$ well separated from the CV seen for **1** under the same conditions.

## Surface analysis of a post-rinse glassy carbon electrode:

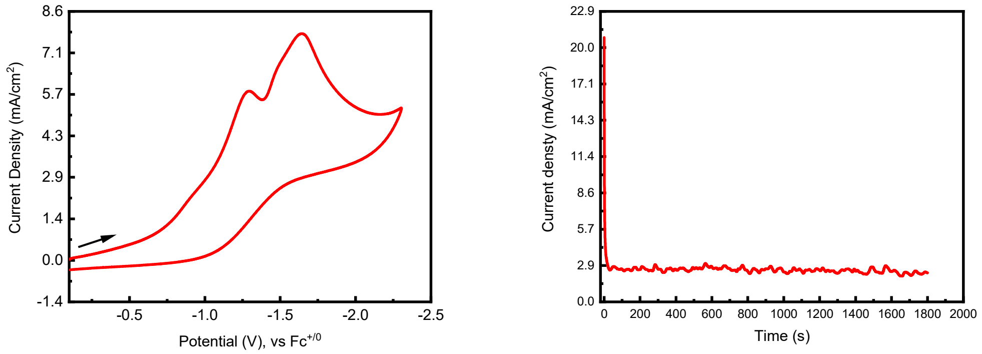


**Fig S41.** A procedure similar to the one described above was used to prepare a glassy carbon disk electrode for surface analysis. A CV of **1** was recorded in the presence of 50 mM TFA (left). Then the same solution was electrolyzed for 1 hour at -1.7 V vs $Fc^{+/0}$ ($i$ vs $t$ trace shown). The glassy carbon electrode was then rinsed with acetone and MeCN and used for SEM and EDX analysis.

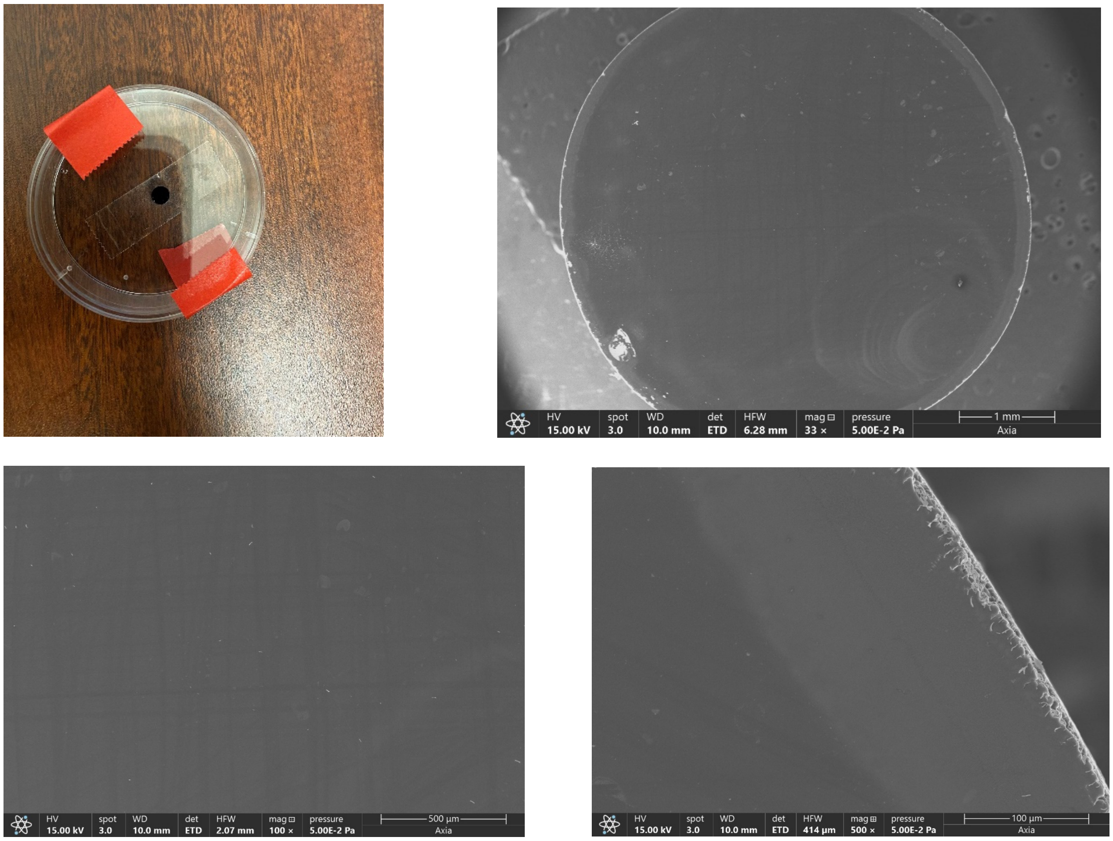

**Figure S42.** (top left) The glassy carbon disk insert used for analysis after rinse test; (top right) SEM image of the glassy carbon disk electrode (the circular object in the image); (bottom left)

SEM image of magnification of the center of the glassy carbon surface; (bottom right) SEM image of edge of the glassy carbon electrode, where deposition is likelier, showing no deposition of Ni.

| Element | Atomic % | Atomic % Error | Weight % | Weight % Error | Net Counts |
|---|---|---|---|---|---|
| C | 91.1 | 0.5 | 89.6 | 0.5 | 37 785 |
| N | 8.9 | 0.7 | 10.2 | 0.8 | 316 |
| O | 0.0 | --- | 0.0 | --- | 0 |
| S | 0.0 | 0.0 | 0.1 | 0.1 | 839 |
| Ni | 0.0 | 0.0 | 0.2 | 0.2 | 22 |

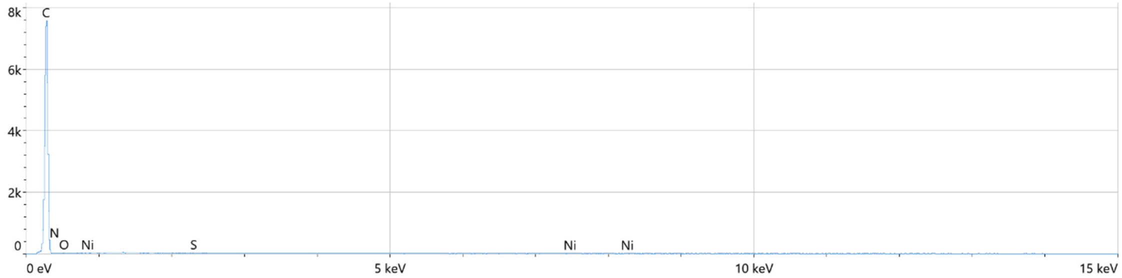


**Figure S43.** Map analysis of the EDX data shows that the amount of Ni or elements from ligand electrodeposited on the surface is minimal.

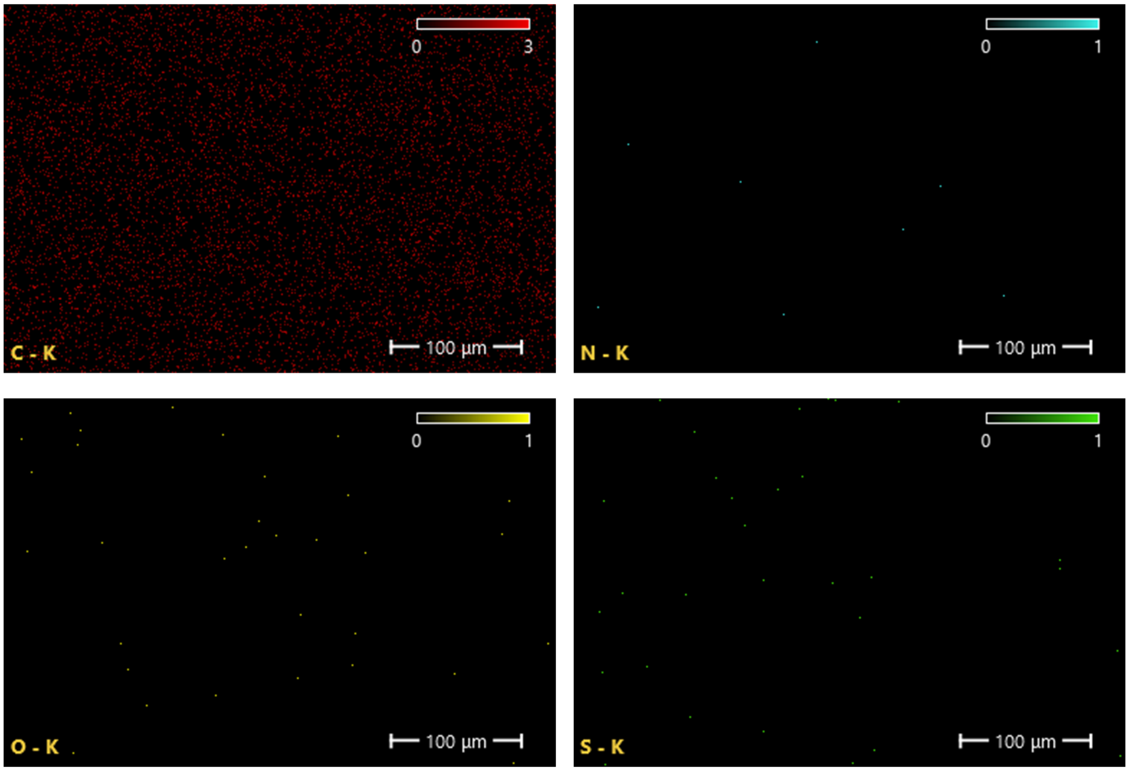

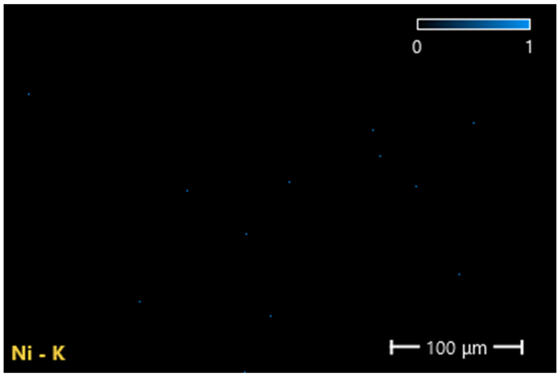


Overall map:

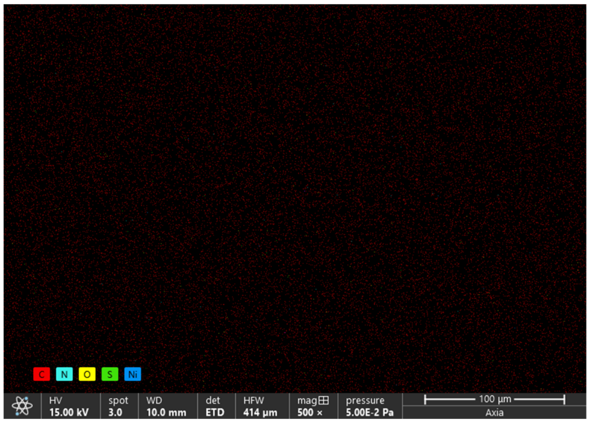


**Figure S44.** Map analysis (map resolution: 1024 x 768) of EDX data showing the frequency of the various elements detected in the analysis.

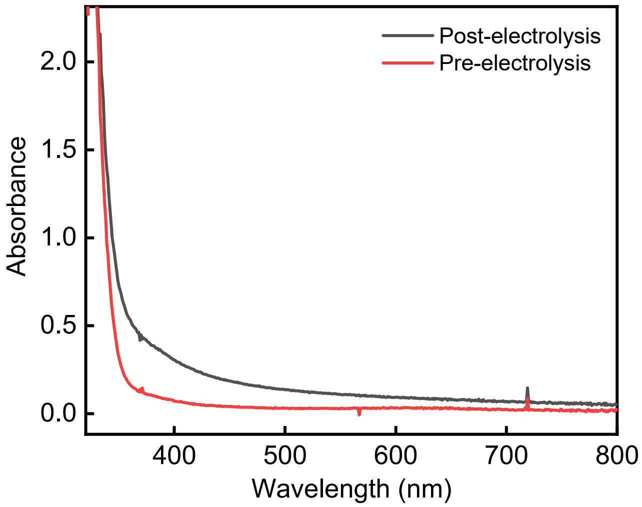


**Figure S45.** UV-vis spectra of pre- and post-electrolysis solutions of **1**.

## Electrolysis experiments data

Chronoamperometric experiments carried out for **1** showed 220 mC of total charge passed over 15 mins of electrolysis at an applied potential of $E_{cat/2}$, in which the GC electrode contributed 52 mC of the charge due to the background acid only reduction (Figure S44). Since the background charge due to the HER performed by the bare carbon electrode is significant, as expected for a strong acid, the applied potential for the controlled potential electrolysis was chosen to be ~$E_{cat/2}$, since the background contribution increases at more negative potentials. Bulk electrolysis was performed for **1** at the applied potential using a carbon cloth electrode (area = 1.5 $cm^2$) in the presence of 0.043 M TFA + 1.5 M $H_2O$ in MeCN, to produce a large amount of $H_2$ in the headspace of the electrochemical cell. The total charge passed over 1 hour of bulk electrolysis was 49.2 C, corresponding to 0.255 mmoles of estimated $H_2$ (Figure S45).

After the bulk electrolysis, 0.239 mmoles of $H_2$ were detected by analyzing the headspace using gas chromatography, corresponding to a TON of 47.8 and an overall Faradaic efficiency (FE) for $H_2$ formation of 94%. Notably, this TON was calculated using the total moles of **1** in solution, rather than the actual concentration of the electroactive catalyst; moreover, catalyst **1** is still active and did not become inactivated after 1 hour of bulk electrolysis, and thus it is difficult to determine the true TON, which is expected to be much larger than 48. Although the background charge passed during the electrocatalytic HER process in the presence of the bare electrode is about 25% vs. the charge passed in the presence of complex **1**, the faradaic efficiency of the background HER process is low (FE <25%) and does not contribute to more than 5-7% of the total $H_2$ produced (Figure S45).

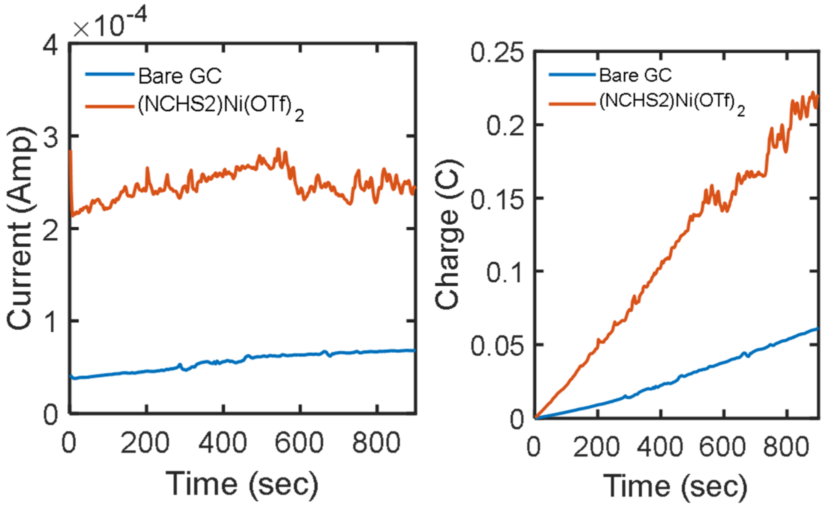


**Figure S46.** Left: Controlled potential electrolysis experiments performed for bare glassy carbon electrode (Bare GC, blue) and **1** (orange) in the presence of 0.043 M TFA and 1.5 M of $H_2O$ added in the MeCN electrolyte at the constant applied potential of $E_{cat/2}$. Right: The corresponding charges passed during the CPE experiments, as shown in the left.

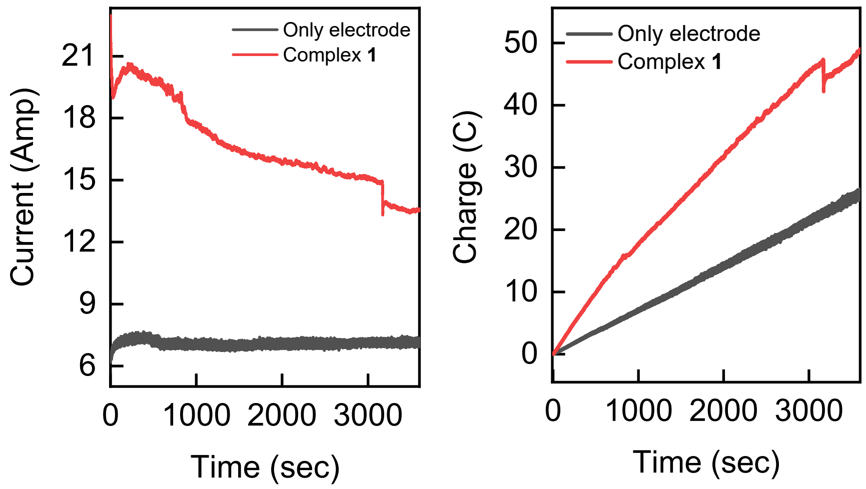


**Figure S47.** Left: Bulk electrolysis performed for bare carbon cloth electrode (black) and **1** (red) in the presence of 0.043 M TFA and 1.5 M of $H_2O$ added in the MeCN electrolyte at the constant applied potential of $E_{cat/2}$. Right: The corresponding charges passed during the CPE experiments, as shown in the left.

## 4. Gas chromatography data

Gas chromatography analysis was performed using Agilent Technologies 7890B GC system equipped with a thermal conductivity detector (TCD). Gas analytes were detected by passing through a HP-Molesieve column (30 m in length, 0.32 mm in diameter, and 25 µm film). The temperature for the detector was set at 220°C and 35°C for the oven. Helium was used as the carrier gas with a flow at 15 mL/min. The calibration curve for $H_2$ was determined by injecting known quantities of pure $H_2$ in MeCN electrolyte (same volume as used for the bulk electrolysis) in the electrolysis cell and by transferring the headspace. The gas sample from the headspace of the post-electrolysis cell was injected using an air-tight syringe (1 mL) and $H_2$ was detected with negative peak area at the retention time of 1.72 min.

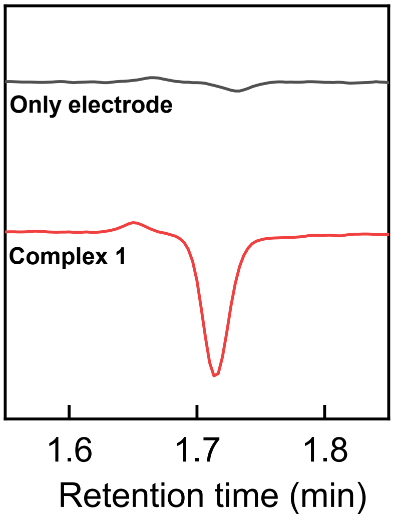


**Figure S48.** GC traces of $H_2$ observed for the bare carbon cloth electrode (black) and **1** (red) by transferring the headspace of the electrochemical cell after performing bulk electrolysis experiments in the presence of 0.043 M TFA + 1.5 M $H_2O$ in the MeCN electrolyte.

## 5. Overpotential, Kinetics and Electrochemical Mechanistic Experiments

### Calculation of Overpotential

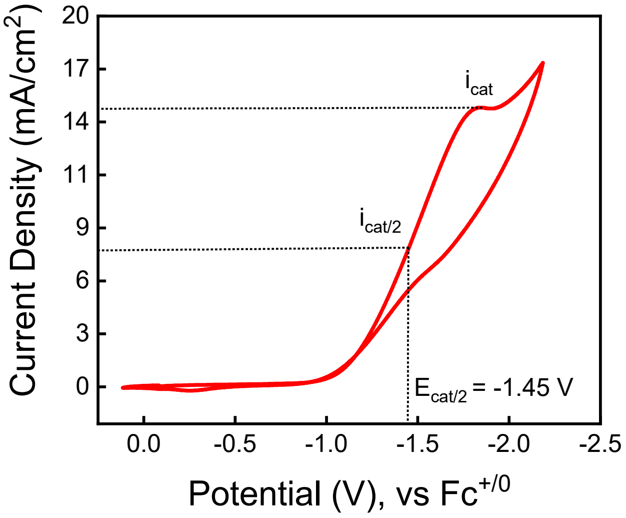


**Figure S49.** CV recorded for **1** in 0.1 M $TBAPF_6$/MeCN in the presence of 0.13 M 1:1 $CF_3COONa$:$CF_3COOH$ buffer, in accordance with the Appel and Helm method.[7] The acid and conjugate base concentrations were chosen based on the presence of a catalytic plateau current.

$$E'(H^+/H_2) = -\ 0.028 - 0.059(pK_a) = -0.028 - 0.059(12.7) = -\ 0.78\ V$$

$$\eta = |E'(H^+/H_2) - E_{cat/2}|$$

$$\text{Therefore, } \eta = |-0.78 - (-1.45)| = 0.67\ V$$

### Kinetic Analysis

Increasing acid titrations into **1** did not produce saturating current densities. So, we increased the scan rate at 0.13 M TFA concentration till the currents reached scan rate independence as shown in Figure S48.[8,9] At a scan rate of 4.5 V/s, the $i_p$ (for $Ni^{II/I}$ under $N_2$) is 2.80 x $10^{-4}$ A and $i_{cat}$ is 5.22 x $10^{-3}$ A. This corresponds to an $i_{cat}/i_p$ of 18.64.

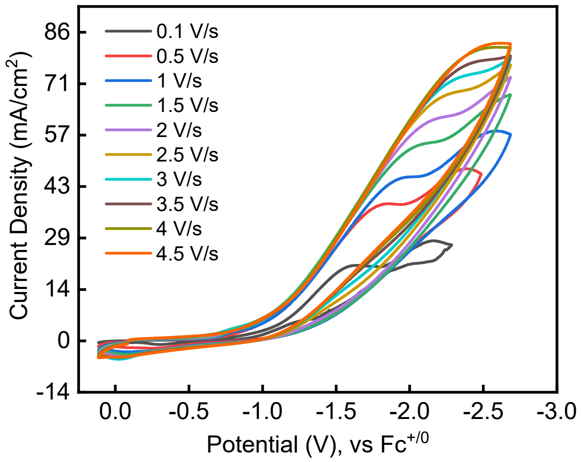


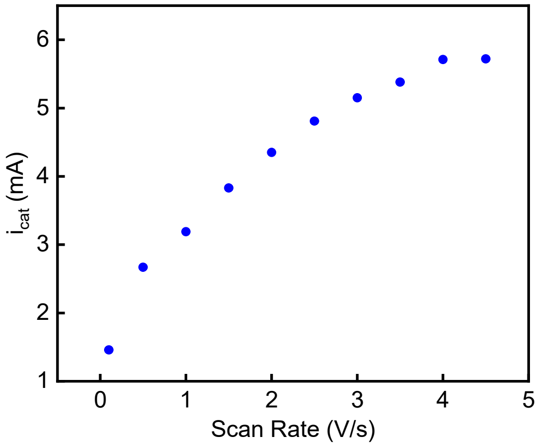


**Figure S50.** Scan rate dependent cyclic voltammograms of **1** with 0.16 M TFA (top) and a plot of $i_{cat}$ *versus* scan rate.

At plateau currents, the following set of equations can be used to calculate $k_{obs}$:

$$i_p = 0.4463FA[cat]\sqrt{\frac{F\nu D}{RT}}$$

$$i_{cat} = nFA[cat]\sqrt{D(k[H^+])}$$

$$k_{obs} = k[H^+]$$

$$k_{obs} = \nu\left(\frac{i_{cat}/i_p}{0.72}\right)^2$$

Accordingly, at ν = 4.5 V/s, $k_{obs}$ = 3016 $s^{-1}$.

A scan rate of 4.5 V/s was then employed to perform TFA titrations

**Determination of order with respect to catalyst**

In order to determine the order of the reaction with respect to the catalyst, serial dilutions of 0.5 mL were done starting from a given catalyst concentration. The concentration of TFA used was fixed in the starting solution and the diluting solution.

The following equation was used to compute the order of the reaction with respect to the catalyst –

$$i_{cat} = nFA[cat]\sqrt{D(k[H^{+}])}$$

A linear relationship between the current density and catalyst concentration is indicative of a first-order dependence.

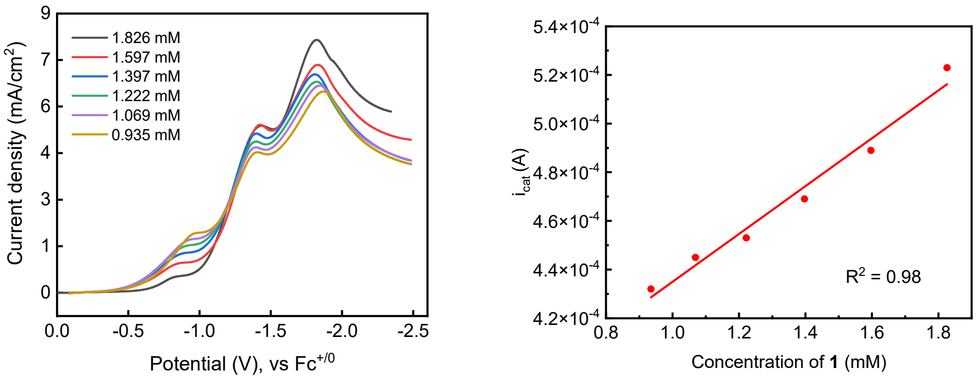


**Figure S51.** CVs recorded for **1** in $N_2$-saturated 0.1 M $TBAPF_6$/MeCN at a concentration of 0.03 M TFA, with concentrations of **1** varying from ~1.8 mM to ~0.9 mM. Only cathodic plots are shown for clarity.

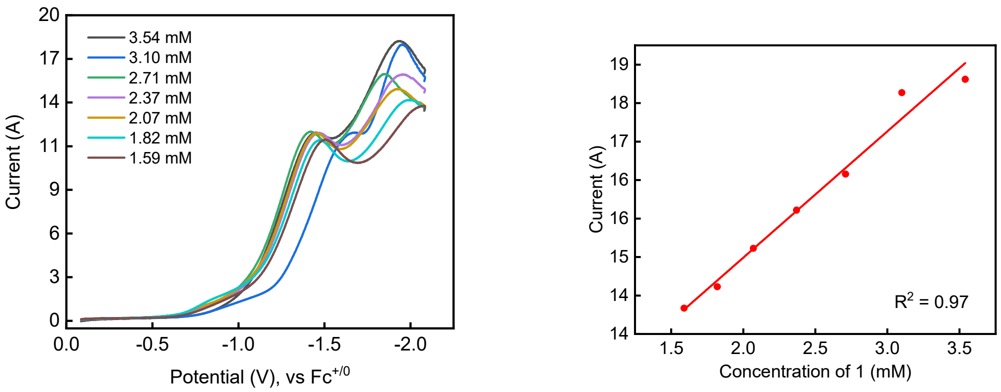


**Figure S52.** CVs recorded for higher concentrations of **1** in $N_2$-saturated 0.1 M $TBAPF_6$/MeCN at a concentration of 0.1 M TFA, with concentrations of **1** varying from ~3.54 mM to ~1.59 mM. Only cathodic plots are shown for clarity.

**Determination of order with respect to acid**

The following equation was used to compute the order of the reaction with respect to the proton source –

$$i_{cat} = nFA[cat]\sqrt{D(k[H^+])}$$

A linear relationship between the current density and the square root of acid concentration is indicative of a first-order dependence.

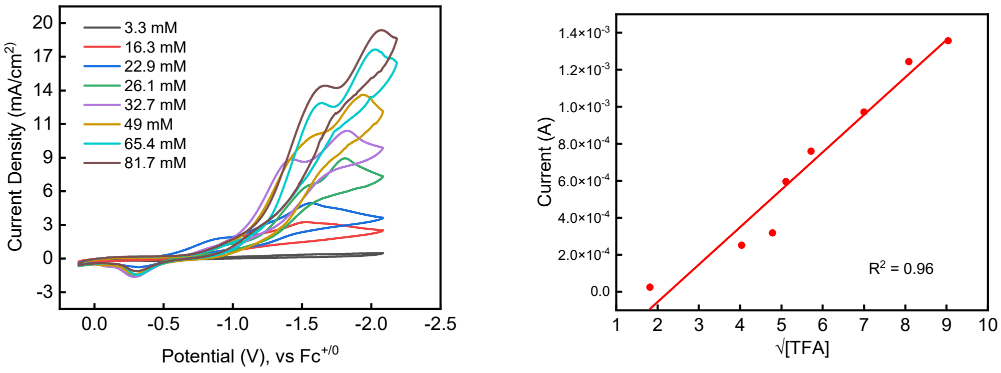


**Figure S53.** TFA titrations into a 1 mM solution of **1** (left) and linear fit of $i_{pc2}$ with the square root of TFA concentration.

In addition, we also computed the $k_{obs}$ at pseudo-plateau currents obtained at a scan rate of 3 V/s.

**Table S2.** Concentrations of TFA and the corresponding $k_{obs}$ and log ($k_{obs}$)

| **Concentration (M)** | $\mathbf{i_{cat}/i_p}$ | $\mathbf{k_{obs}\ (s^{-1})}$ | **log** $\mathbf{k_{obs}}$ |
|---|---|---|---|
| | | | |
| 0.03 | 4.84 | 135.49 | 2.13 |
| 0.07 | 8.46 | 414.07 | 2.62 |
| 0.10 | 10.03 | 582.86 | 2.77 |
| 0.13 | 12.37 | 884.88 | 2.95 |
| 0.16 | 15.38 | 1368.24 | 3.14 |

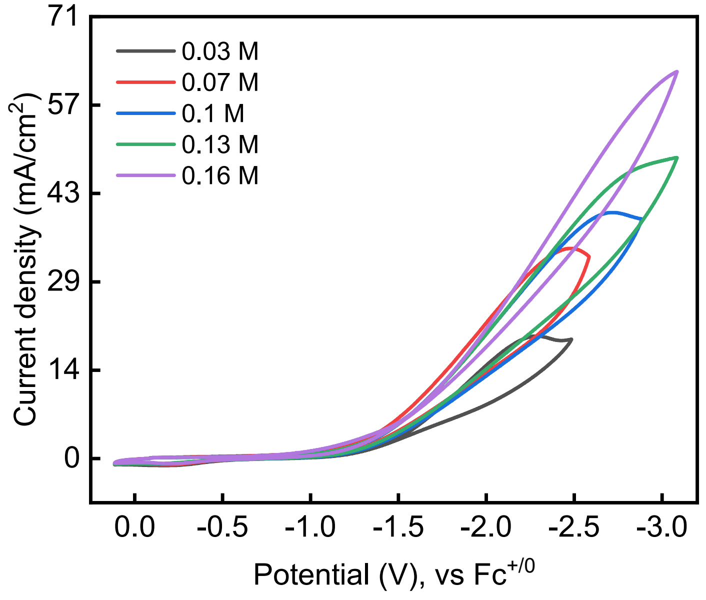


**Figure S54.** Cyclic voltammograms of **1** recorded at 3 V/s with increasing concentrations of TFA. While the currents obtained are not canonical S-shaped voltammograms, the $k_{obs}$ values obtained from current enhancements should approximate the order of the reaction in acid.

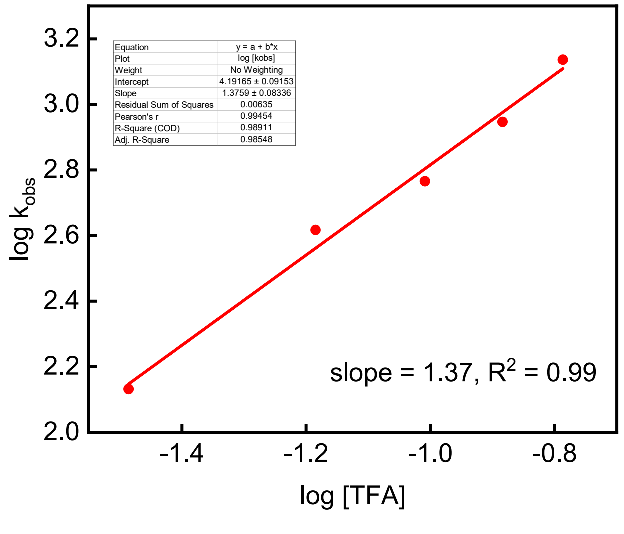


**Figure S55.** The double log plot of $k_{obs}$ and [TFA] has a slope of 1.37, suggestive of a first order reaction in acid.

**Kinetic Isotope Effect Experiments**

The following equation was used to calculate the electrochemical kinetic isotope effect.

$$k[H^+] = \nu\left(\frac{i_{cat}/i_p}{0.72}\right)^2$$

Therefore, at the same scan rate:

$$k_H \propto (i_{cat}/i_p)^2$$

For a plot of $k_H$ or $k_D$ *versus*

$$KIE = k_H/k_D = (slope,H/slope,D)^2$$

Five independent experiments were carried out to get an average of the kinetic isotope effect. A typical set of cyclic voltammograms recorded for $CF_3COOH$ and $CF_3COOD$ (99%) is shown followed by the $i_{cat}/i_p$ *versus* $[H^+]$ plots for the five runs. For every case, higher currents were obtained for $CF_3COOD$, with the difference becoming more pronounced at higher concentrations of acid. The plots of the calculated KIEs are shown below, for $E_{cat1}$ first and $E_{cat2}$ after that:

(a) For $E_{cat1}$

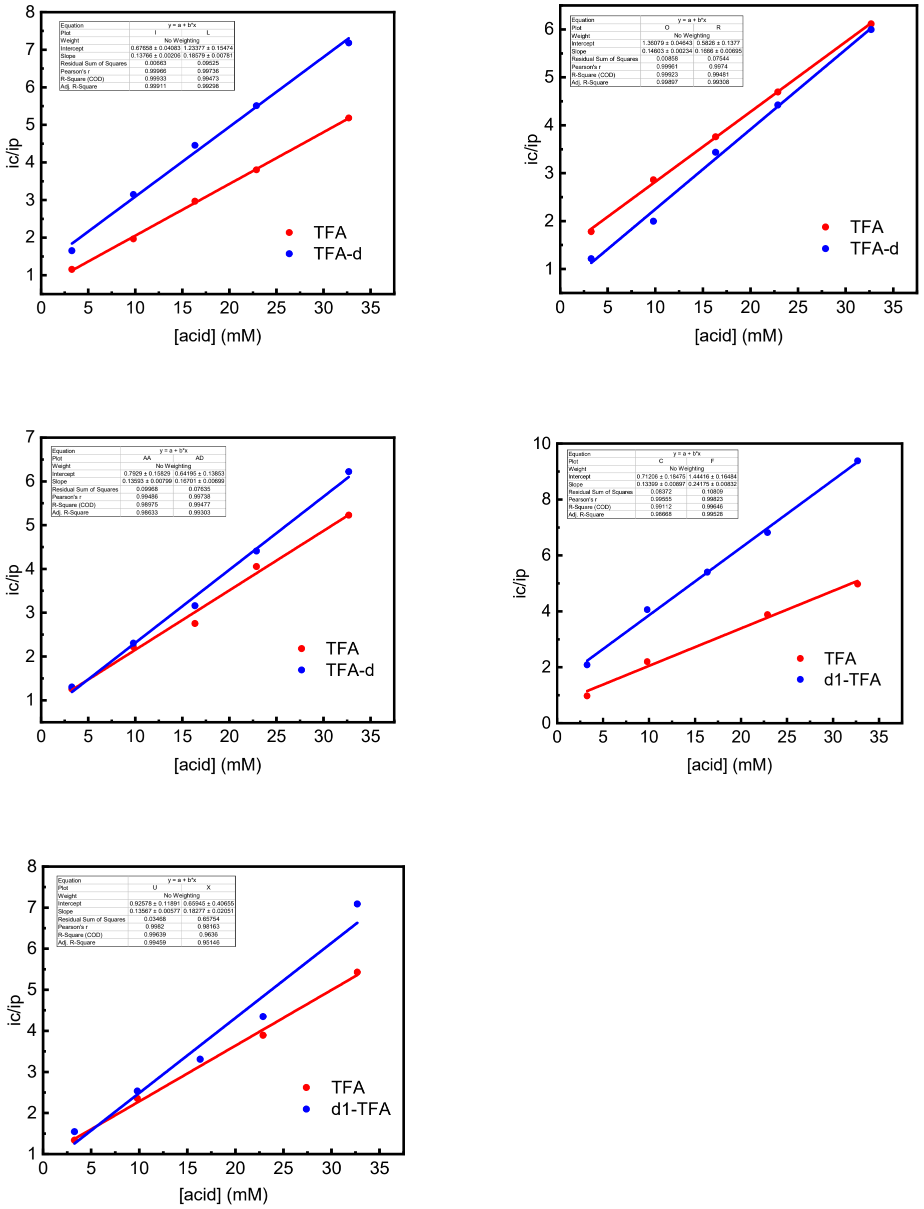


**Figure S56.** Plots of $i_c/i_p$ *versus* concentration of $CF_3COOH/CF_3COOD$ for $E_{cat1}$.

(b) For $E_{cat2}$

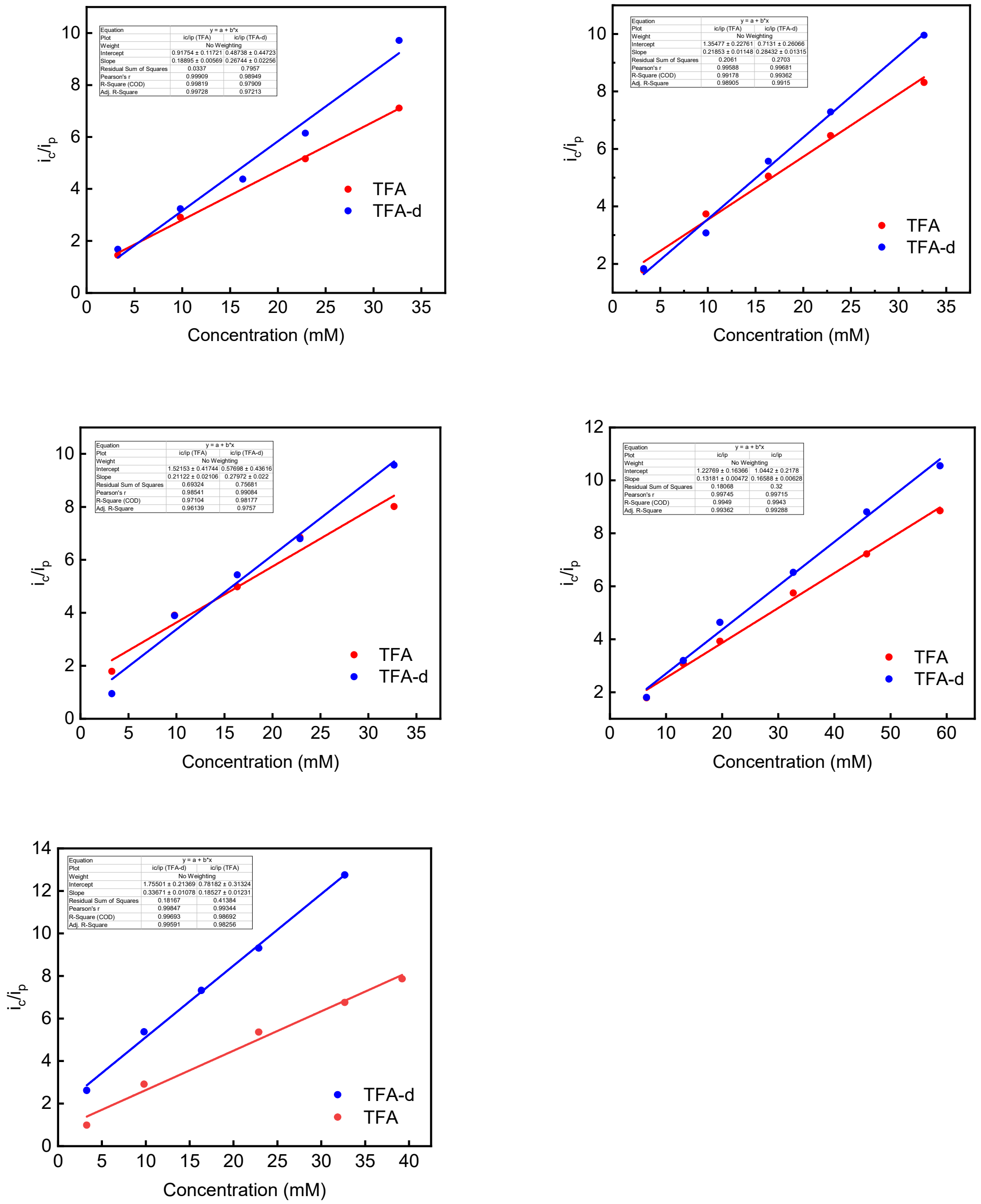


**Figure S57.** Plots of $i_c/i_p$ *versus* concentration of $CF_3COOH/CF_3COOD$ for $E_{cat2}$.

**Table S3.** Calculated electrochemical kinetic isotope effects.

For $E_{pc1}$:

| Run | $Slope_H$ | $Slope_D$ | $[Slope_H/Slope_D]$ | $[Slope_H/Slope_D]^2$ |
|---|---|---|---|---|
| | | | | |
| 1 | 0.138 ± 0.002 | 0.185 ± 0.008 | 0.75 ± 0.05 | 0.56 ± 0.2 |
| 2 | 0.146 ± 0.007 | 0.166 ± 0.002 | 0.88 ± 0.05 | 0.77 ± 0.2 |
| 3 | 0.136 ± 0.006 | 0.18 ± 0.02 | 0.76 ± 0.08 | 0.58 ± 0.3 |
| 4 | 0.136 ± 0.008 | 0.167 ± 0.007 | 0.81 ± 0.08 | 0.65 ± 0.3 |
| 5 | 0.134 ± 0.008 | 0.242 ± 0.008 | 0.55 ± 0.05 | 0.30 ± 0.2 |

For $E_{pc2}$:

| Run | $Slope_H$ | $Slope_D$ | $[Slope_H/Slope_D]$ | $[Slope_H/Slope_D]^2$ |
|---|---|---|---|---|
| | | | | |
| 1 | 0.19 ± 0.01 | 0.27 ± 0.02 | 0.70 ± 0.06 | 0.50 ± 0.09 |
| 2 | 0.22 ± 0.01 | 0.28 ± 0.01 | 0.79 ± 0.04 | 0.61 ± 0.06 |
| 3 | 0.21 ± 0.02 | 0.28 ± 0.01 | 0.75 ± 0.08 | 0.56 ± 0.1 |
| 4 | 0.13 ± 0.01 | 0.17 ± 0.01 | 0.76 ± 0.04 | 0.58 ± 0.06 |
| 5 | 0.19 ± 0.01 | 0.34 ± 0.01 | 0.56 ± 0.03 | 0.31 ± 0.05 |

Based on the above, the kinetic isotope effect for **1** using $CF_3COOH$ and $CF_3COOD$ as proton sources, the average KIE is 0.57 ± 0.2 for $E_{cat1}$ and 0.51 ± 0.09 for $E_{cat2}$. Two catalytic peaks suggest two separate HER mechanisms operating. We would like to note that diminished FEs were observed at lower overpotentials. Under pure kinetic conditions (Fig. S49), the two-peak feature is replaced by a single plateau wave, which suggests one mechanism operating under those conditions. It is also worth noting that for the lower overpotential regime, in presence of lower concentrations of acid, the $E_{cat2}$ is very close to the peak from glassy carbon and could obfuscate analysis. This would also explain the higher uncertainties in the KIE.

As a comparison for a system with a similar ligand framework that also catalyzes HER, we performed KIE experiments with the related nickel complex [(N2S2)Ni$^{II}$(MeCN)$_2$][BF$_4$]$_2$. Electrochemical HER with this system was published recently and was shown to go through a Ni$^0$/Ni$^{II}$-H cycle.[10]

(a)

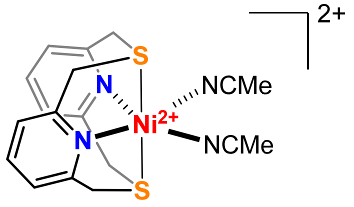


(b)

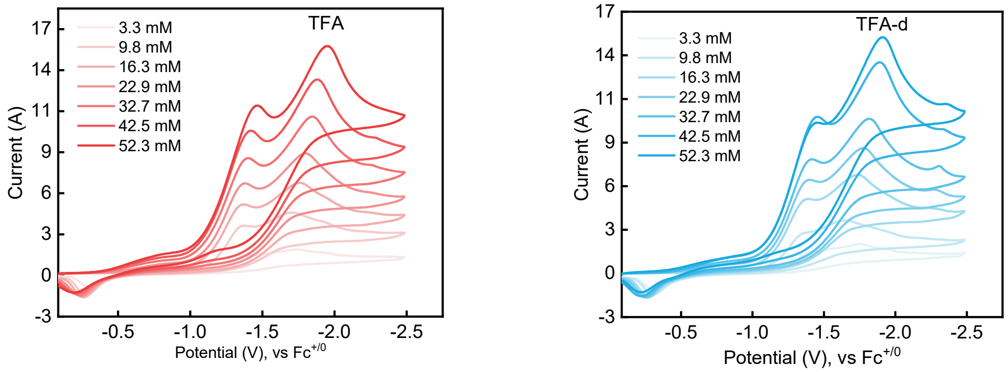


(c)

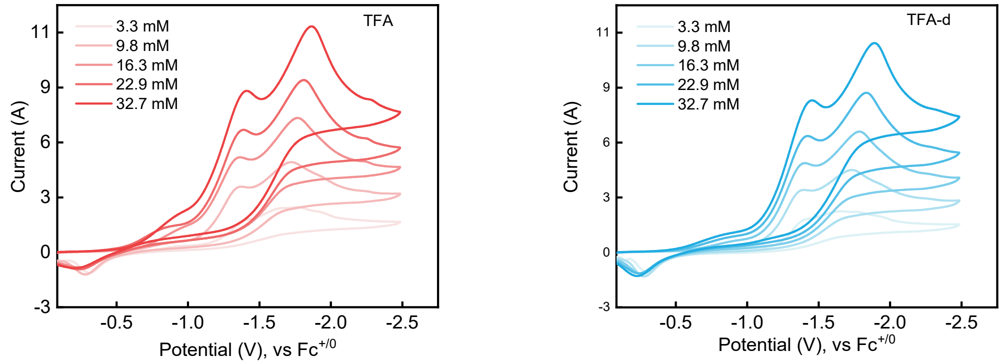


**Figure S58.** (a) Molecular structure of [(N2S2)Ni$^{II}$(MeCN)$_2$][BF$_4$]$_2$ (b) and (c). Catalytic cyclic voltammograms (CVs) recorded in 0.1 M TBAPF$_6$/MeCN for the same concentrations of [(N2S2)Ni$^{II}$(MeCN)$_2$][BF$_4$]$_2$ in the presence of increasing concentrations of CF$_3$COOH (red) and CF$_3$COOD (blue).

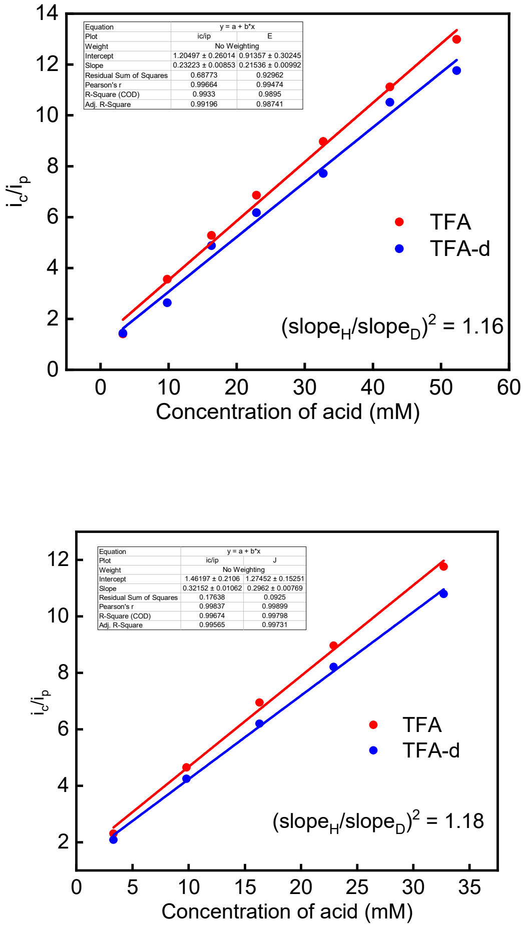


**Figure S59.** Plots of $i_c/i_p$ *versus* concentration of acid (TFA - red, TFA-d – blue) for evaluating the kinetic isotope effect for $[(N2S2)Ni(MeCN)_2]^{2+}$.

The mechanism of proton reduction by $[(N2S2)Ni^{II}(MeCN)_2]^{2+}$ was proven to follow a $Ni^0/Ni^{II}$-H pathway in a recent report from our group.[10] The small, positive KIE of 1.17(1) for this process indicates that protons are probably not involved in the rate determining step. The close structural similarity of this complex with **1**, but a different value of KIE obtained by the same method suggests a difference in the mechanism of hydrogen evolution.

## 6. Absorption spectra

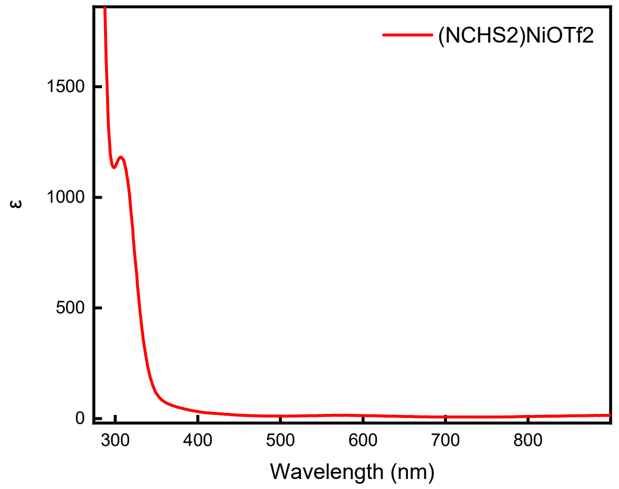


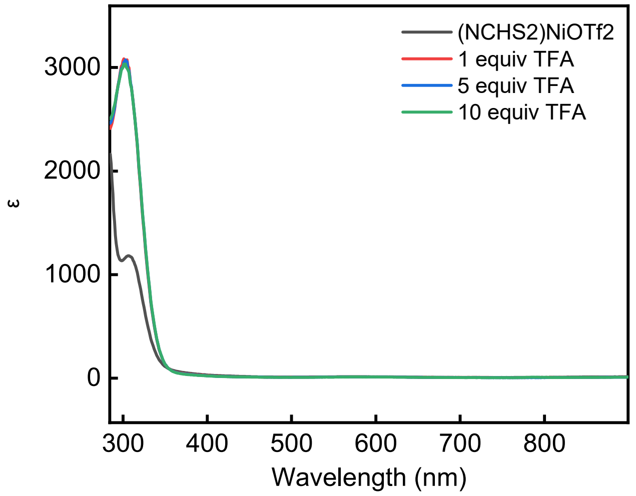


**Figure S60.** Absorption spectra collected for **1** (1.5 mM, left) in MeCN solution. The $\lambda_{max}$ values are 308 nm. Absorption spectra collected for **1** (1.8 mM) in the MeCN solution in the absence of TFA (blue) and in the presence of excess TFA (1 to 10 equivalents) as shown in the legend.

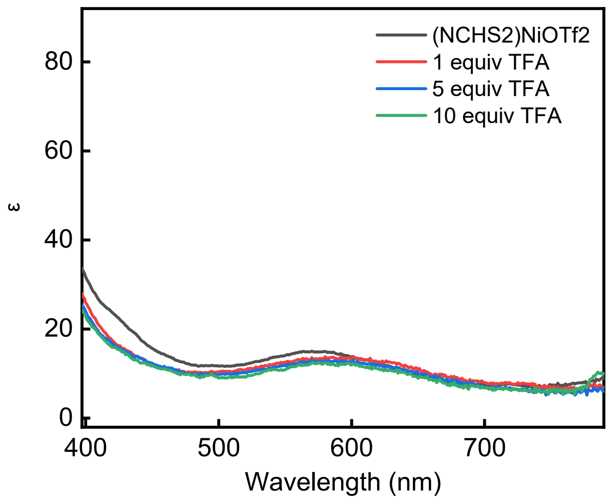


**Figure S61.** The d-d transitions for the complex (588 nm, ε = 14 L $mol^{-1}$ $cm^{-1}$) do not change as such with addition of acid. This proves that **1** is quite stable even in excess acid and protodemetalation is not a problem.

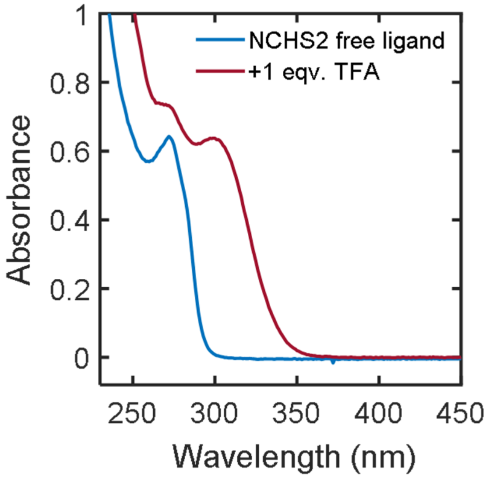


**Figure S62.** Absorption spectra collected for NCHS2 free ligand (1.5 mM) in the MeCN solution in the absence of TFA (blue) and the presence of 1 equivalent TFA (maroon). Note: an additional peak with $\lambda_{max}$ at 302 nm was observed after the addition of TFA.

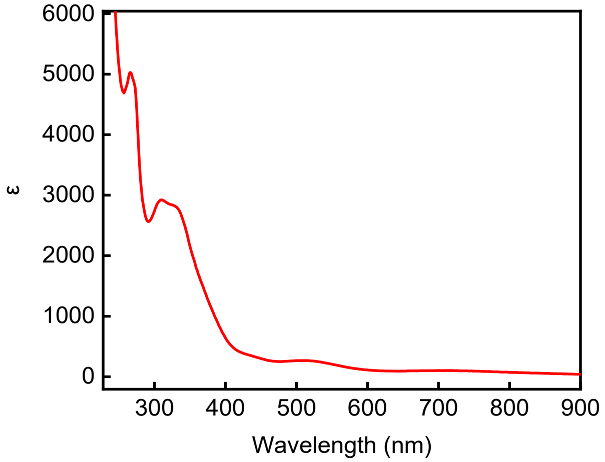


**Figure S63.** Absorption spectra collected for **[2]**$^{+}$ (0.8 mM, left) and in MeCN solution. The $\lambda_{max}$ values observed for 2 are 265, 320 nm (ligand-based), 513, 720 nm (d-d).

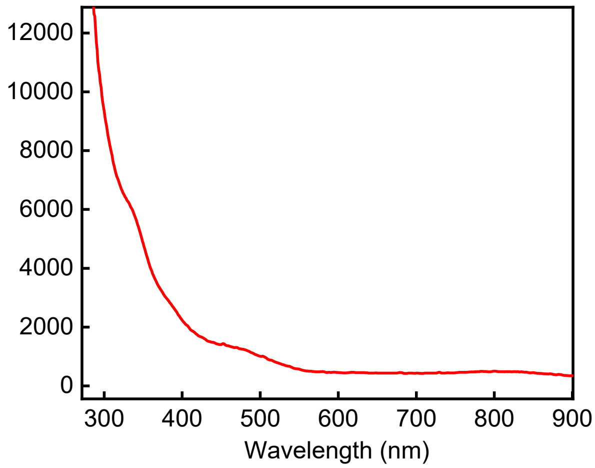


**Figure S64.** Absorption spectra collected for **[2-Br]** (0.4 mM, left) and in MeCN solution. The $\lambda_{max}$ values observed for 2 are 335 nm (ligand-based), 470 nm (MLCT).

## 7. EPR studies of Ni complexes

*General procedure EPR analysis.* An EPR tube was charged with a solution of Ni complex in 1:3 MeCN:PrCN (butyronitrile), which was cooled in an acetone/dry ice cold bath. To that solution was added a 1:3 MeCN:PrCN solution containing 1 equiv of $CoCp_2$, $CoCp^*_2$, or $NOPF_6$. The resulting solution was shaken for 5 seconds and then frozen in liquid nitrogen. Typical experimental conditions: frequency ≈ 9.096 GHz, power = 1 mW, modulation frequency = 100 kHz, modulation amplitude = 3 G, time constant = 0.3 s, linewidth = 15 G.

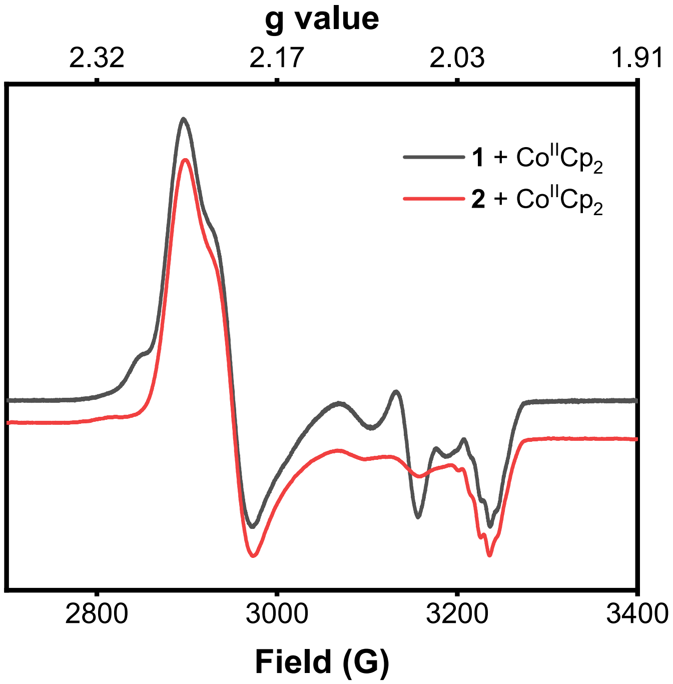


**Figure S65.** Overlaid experimental (MeCN:PrCN, 77K) EPR spectra of **1** + $CoCp^*_2$ (dark grey) and **2** + $CoCp^*_2$ (red).

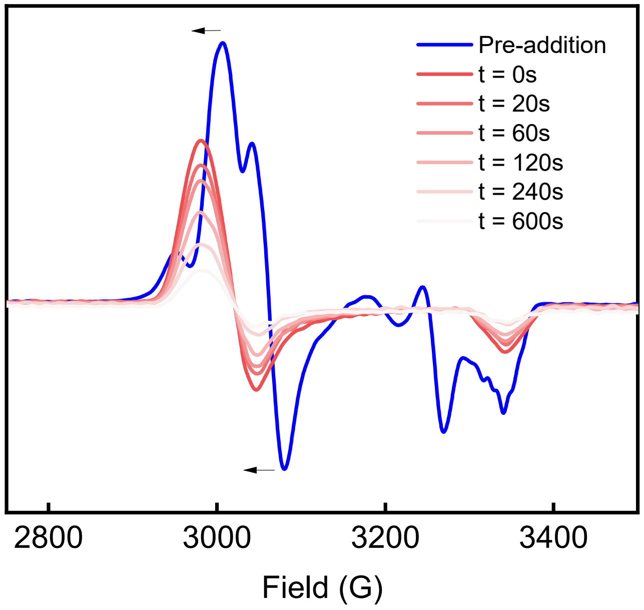


**Figure S66.** EPR spectrum change of **1** + $CoCp^*_2$ with addition of 1 equiv TFA over time. Blue solid line represents the initial EPR spectrum of **1** + $CoCp^*_2$**.** Each spectrum was recorded in 1:3 MeCN:PrCN glass, 77K.

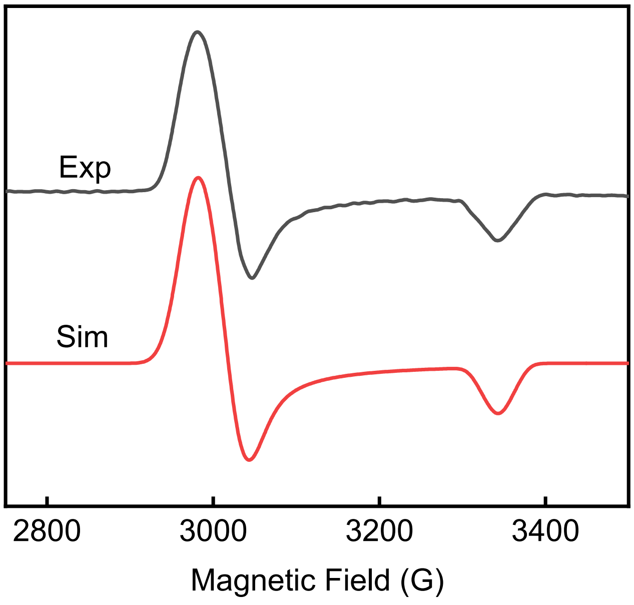


**Figure S67.** Simulation of **1** + $CoCp^*_2$ + TFA. The following parameters were used for simulating the spectrum: $g_x = 2.254$, $g_y = 2.228$, $g_z = 2.012$ ($A_{2N} = 12$ G). The decreased anisotropy for this paramagnetic species suggests a more symmetric ligand field.

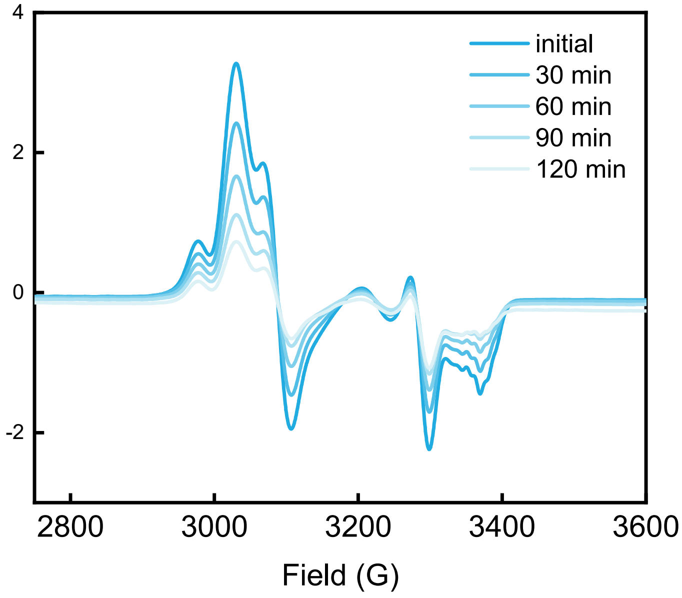


**Figure S68.** EPR spectra for assessing the stability of the $Ni^{I}$ complex generated on adding $CoCp*_2$ to **1**. All warmups were done in a dry ice/acetonitrile bath (-35 ºC). Both species (corresponding to Sim 1 and Sim 2) decay at the same rate.

To probe the formation of the $Ni^{III}$-H, we reacted a 1:3 MeCN:PrCN solution of NCHS2 and NCDS2 to an *in situ* generated $Ni^{I}$ (from $[Ni^{II}(MeCN)_6]^{2+}$ and $KC_8$). The observations suggest that they react differently.

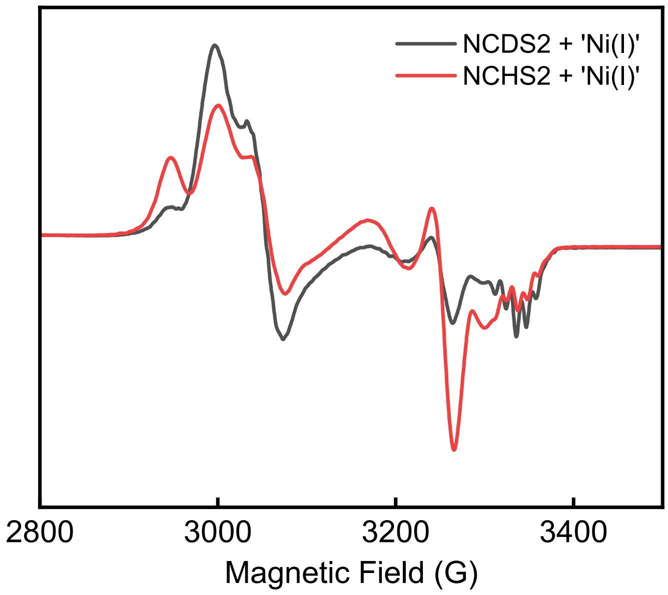

**Figure S69.** EPR spectra of NCDS2 + ‘$Ni^{I}$’ and NCHS2 + ‘$Ni^{I}$’, normalized with respect to the total area under the curve. As is expected, the stronger *ipso* C-D bond in NCDS2 gives rise to a lower population of the species corresponding to *Sim 2*.

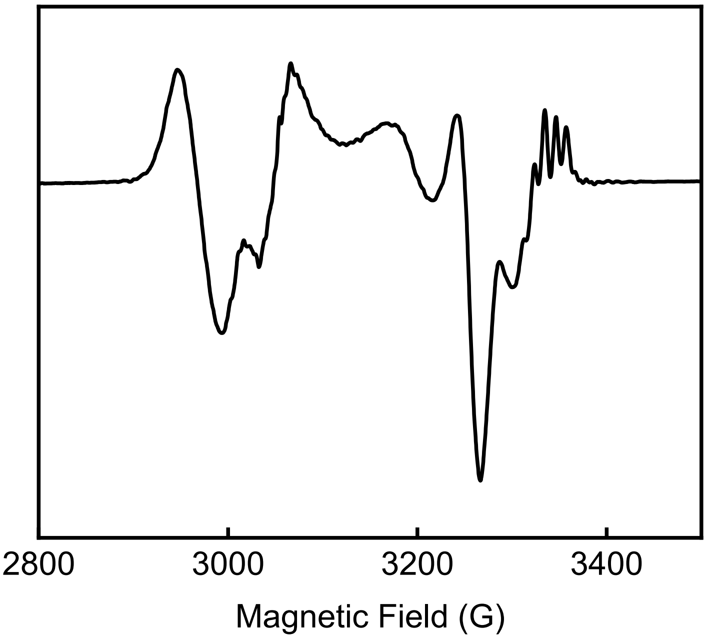


**Figure S70.** Difference spectrum of the EPR spectra of NCHS2 + ‘$Ni^{I}$’ and NCDS2 + ‘$Ni^{I}$’.

In addition, we also monitored the EPR spectra of reducing (NCHS2)$NiOTf_2$ vs (NCDS2)$NiOTf_2$ with $CoCp^*_2$.

**Table S4:** Differences between simulation parameters of the EPR scale reduction of (NCHS2)$Ni^{II}OTf_2$ vs (NCDS2)$Ni^{II}OTf_2$ with $CoCp^*_2$.

<table>
<tr><td></td><td>(NCHS2)$Ni^{II}$ + $CoCp^*_2$</td><td>(NCDS2)$Ni^{II}$ + $CoCp^*_2$</td></tr>
<tr><td></td><td>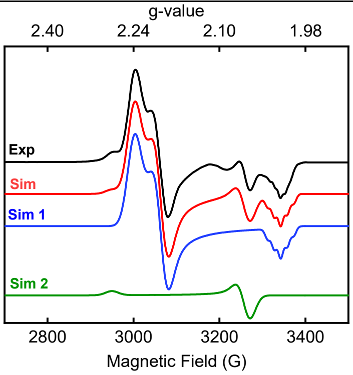
</td><td>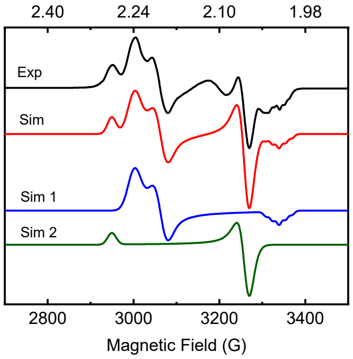
</td></tr>
<tr><td>Sim 1 – g<br>LW</td><td>[2.240, 2.196, 2.013 ($A_{2N}$ = 15 G)]<br>[30, 25, 18] G</td><td>[2.241, 2.196, 2.015 ($A_{2N}$ = 14 G)]<br>[30, 28, 12] G</td></tr>
<tr><td>Sim 2 - g<br>LW</td><td>[2.282, 2.062, 2.062]<br>[30, 30, 30] G</td><td>[2.281, 2.062, 2.062]<br>[20, 35, 20] G</td></tr>
<tr><td>Sim1:Sim2</td><td>1:4.5</td><td>1:2</td></tr>
</table>

The simulation parameters suggest subtle differences in the spectra, however the failure to reliably generate sufficient amounts of $Ni^{III}$ prevents its characterization using pulsed EPR or vibrational spectroscopy. It has already been established CW EPR cannot reliably differentiate between hydride and deuteride coupling.[11]

In fact, given that **1** is a competent electrocatalyst for hydrogen evolution, we think it is reasonable to not be able to reliably generate and characterize a short-lived intermediate like $Ni^{III}$-H with this system alone. Synthesizing a $Ni^{III}$-H would require making complexes which are poorer catalytically, which is an active research direction underway in our lab. However, to provide preliminary evidence of being able to support a $Ni^{III}$-H species with this ligand, we conducted the following experiments –

(a) Reaction of *in situ* generated $Ni^{III}$-OPh species with HBPin

A second method of forming Ni-H complexes is the reaction of Ni-OR species with E-H bonds (E = B, Si) to take advantage of the thermodynamic favorability of the formation of strong E-O bonds. In fact, such a synthetic strategy was elegantly employed by Liaw and coworkers to generate the first reported example of a $Ni^{III}$-H.[12] To test similar reactivity with the (NCS2)$Ni^{III}$ systems, we added 3 equiv NaOPh to a 1:3 MeCN:PrCN EPR solution of **[2]**$^+$. This resulted in a clean conversion of the axial spectrum to a rhombic spectrum, which is reminiscent of the related ($^{tBu}$N3C)$Ni^{III}(OMe)_2$ complex previously reported by our group.

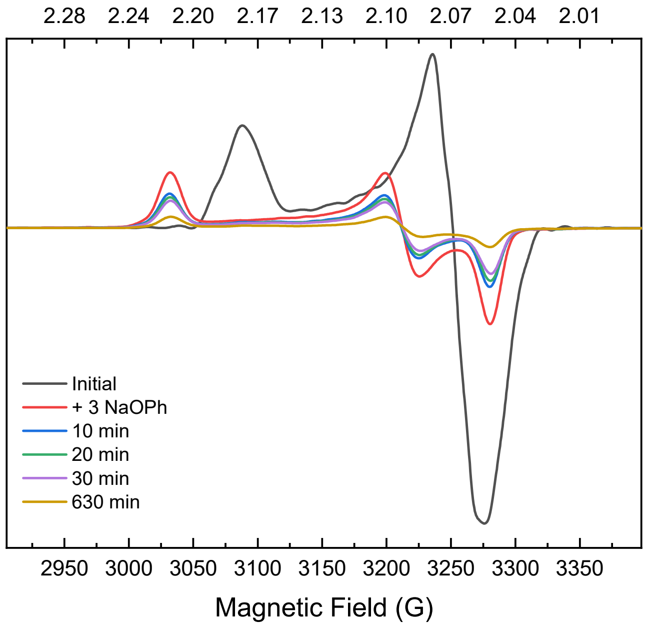


**Figure S71.** EPR spectra of reaction of **[2]**$^+$ in 1:3 MeCN:PrCN with the addition of 3 equivalents NaOPh. Warmups were done at -35 ºC.

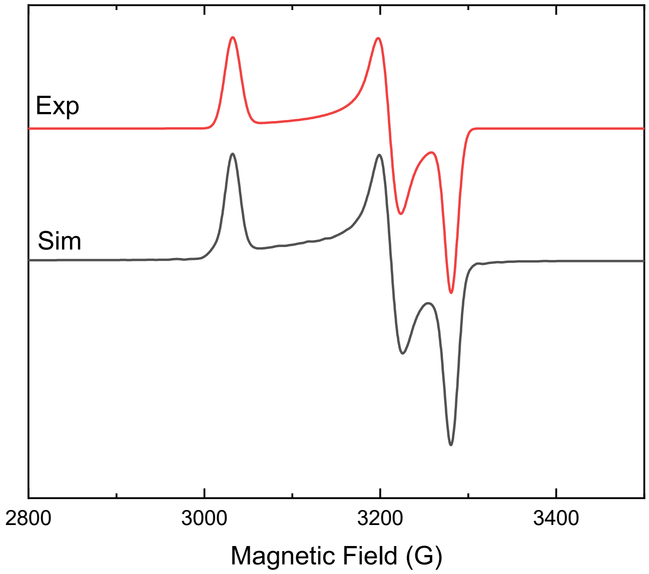


**Figure S72.** Simulation of **[2]**$^+$ + NaOPh. The following parameters were used for simulating the spectrum: $g_x = 2.218$, $g_y = 2.095$, $g_z = 2.050$. The simulation parameters for **[2]**$^+$ are g = [2.184, 2.062, 2.055].

On adding ~4 equiv HBPin to this EPR solution, the rhombic spectrum changed to a mixture of an inverted axial and a rhombic spectrum, with g-tensors that resemble the mixture of paramagnetic species we obtain on reducing **1**. This observation suggests that reduction of a $Ni^{I}$ species or the reaction of a *bona fide* $Ni^{III}$-OR species with a B-H bond results in a mixture that suggests similar chemistry going on in solution. While further characterization of this 'paramagnetic soup' is necessary to unequivocally assign the identity of each species in solution, we believe that these EPR experiments suggest that these Ni complexes exist in an equilibrium of $Ni^{I}$ and $Ni^{III}$-H.

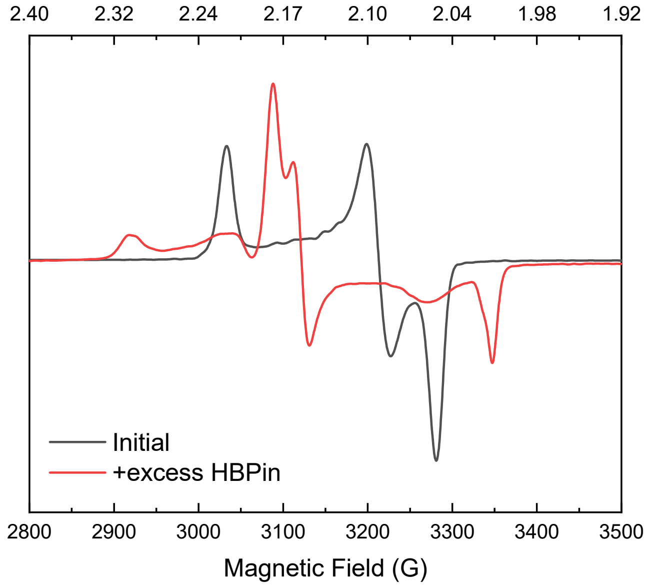


**Figure S73.** EPR spectra of reaction of **[2]**$^{+}$ in 1:3 MeCN:PrCN with the addition of 3 equivalents NaOPh, followed by the addition of 3 equivalents HBPin. The pinacolborane was added as a solution to a cold (-78 ºC) solution of **[2]**$^{+}$ + NaOPh.

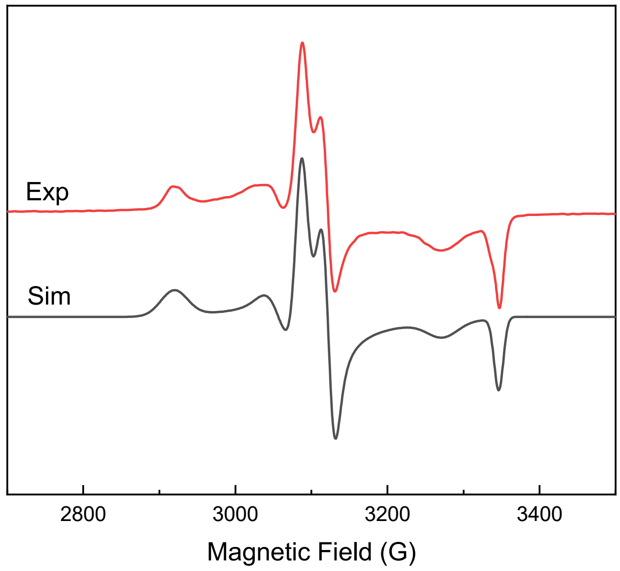


**Figure S74.** Simulation of **[2]**$^+$ + NaOPh + HBPin. The simulation parameters are shown in Fig. S77.

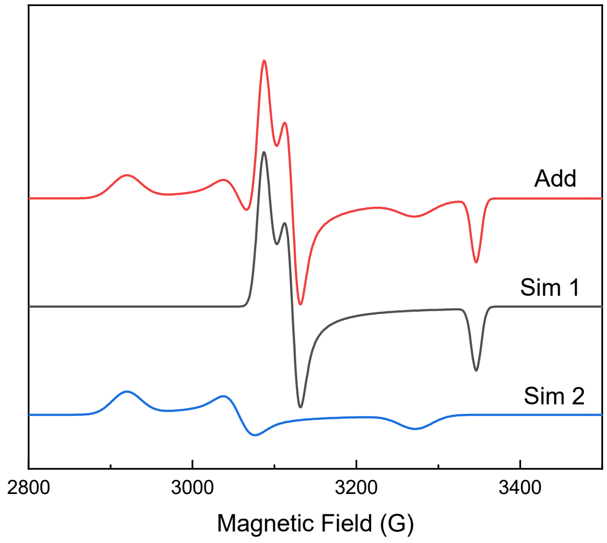


**Figure S75.** Simulation of **[2]**$^+$ + NaOPh + HBPin. The following parameters were used for simulating the spectrum: Sim 1: $g_1 = 2.179$, $g_2 = 2.154$, $g_3 = 2.01$ and Sim 2: $g_1 = 2.304$, $g_2 = 2.200$, $g_3 = 2.055$.

On comparing the two sets of EPRs, we see the following key similarities –

(a) 'Sim 1', which is assigned to a $Ni^{I}$ species in both cases is an inverted axial spectrum, with no coupling in $g_3$ for the phenolate species, as would be expected for an oxygen bound species.

(b) 'Sim 2' in both cases have similar high and low field g-values and the differences in the EPR could be due to differences in geometry and coordination environment.

Importantly, in the first method, we propose a $Ni^{I}/Ni^{III}$ equilibrium by the oxidative cleavage of a C-H bond by a reduced Ni species. While this electron transfer process has no precedent, it is significant we were able to see a very similar mixture of EPR species generated in a redox-neutral fashion, employing chemistry that has significant literature precedent, starting from a *bona fide* $Ni^{III}$ complex.

**Table S5:** Comparison of the simulation parameters of **1** + $CoCp^{*}_2$ and **[2]**$^+$ + NaOPh + HBPin.

| | (NCHS2)$Ni^{II}$ + $CoCp^{*}_2$ | (NCS2)$Ni^{III}(OPh)_2$ + HBPin |
|---|---|---|
| | 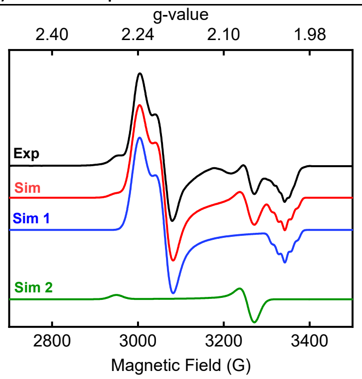 | 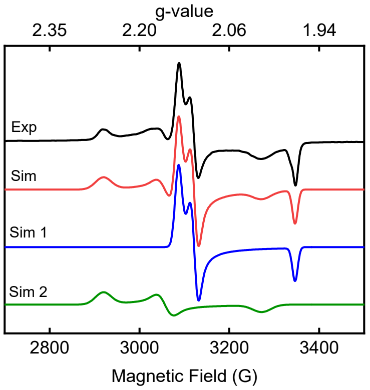 |
| Sim 1 | [2.240, 2.196, 2.013 ($A_{2N}$ = 15 G)] | [2.179, 2.154, 2.01] |
| Sim 2 | [2.282, 2.062, 2.062] | [2.304, 2.200, 2.055] |
| Ratio of Sim1:Sim2 | 1:4.5 | 1:6.7 |

## 8. Infrared Spectroscopy Data

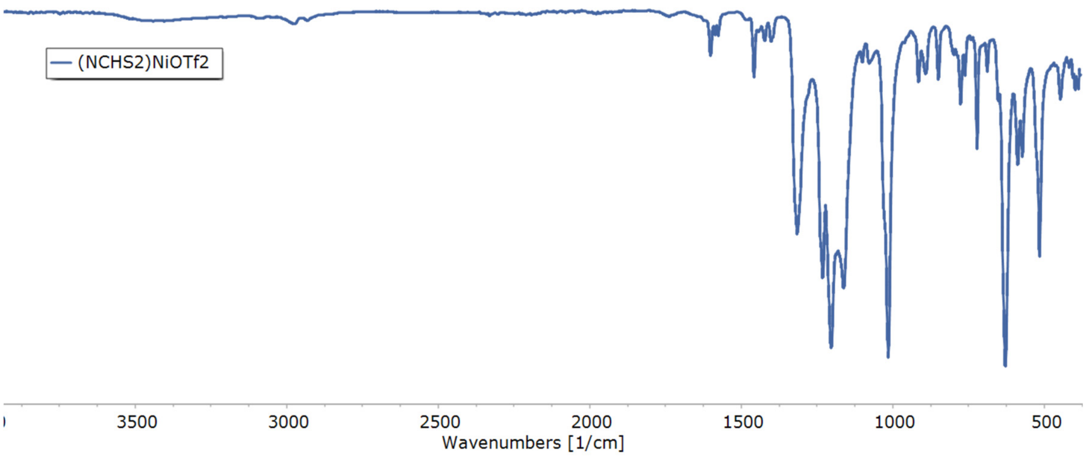


**Figure S76.** IR spectrum for **1**

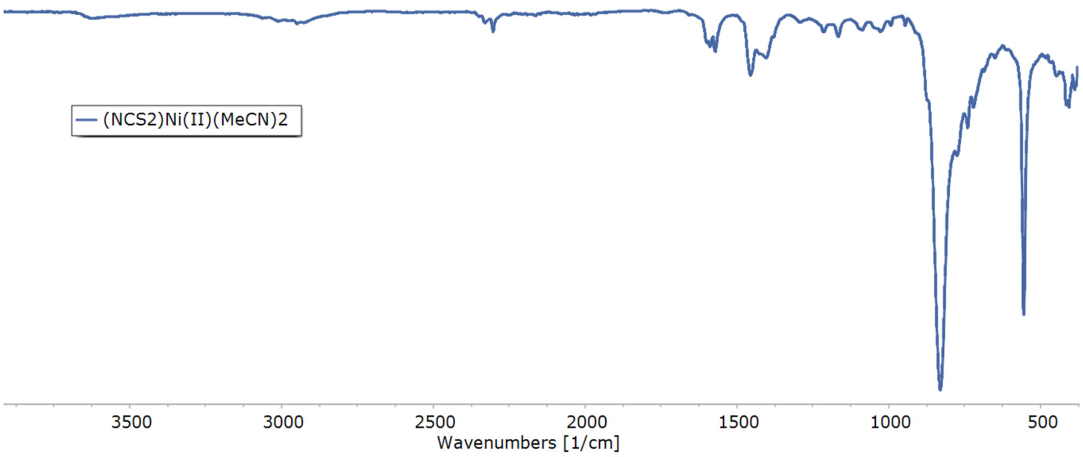


**Figure S77.** IR spectrum for **2**

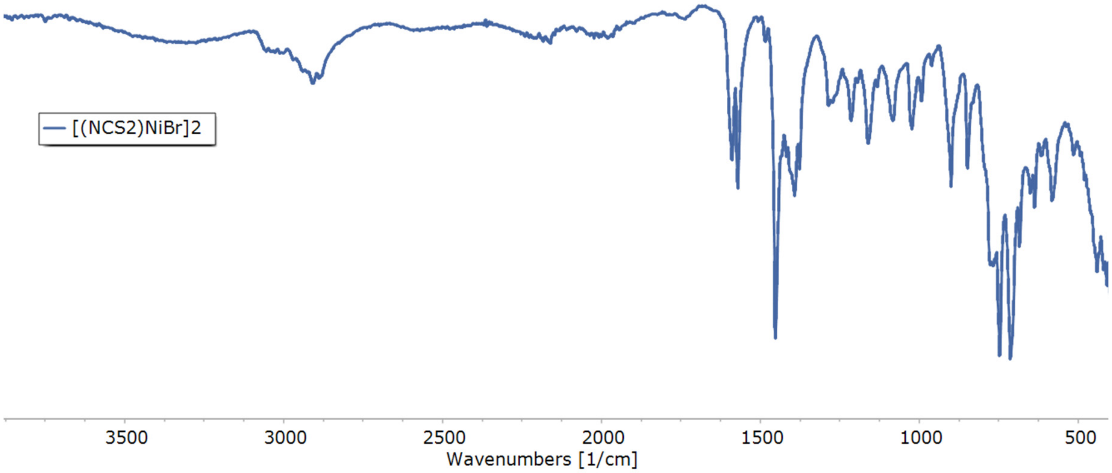


**Figure S78.** IR spectrum for **[2-Br]**

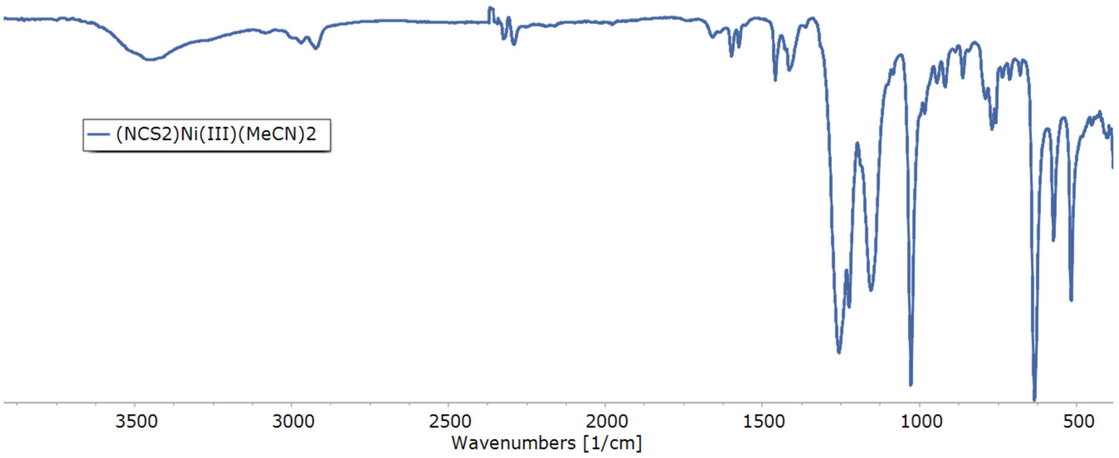


**Figure S79.** IR Spectrum for **[2]**$^{+}$

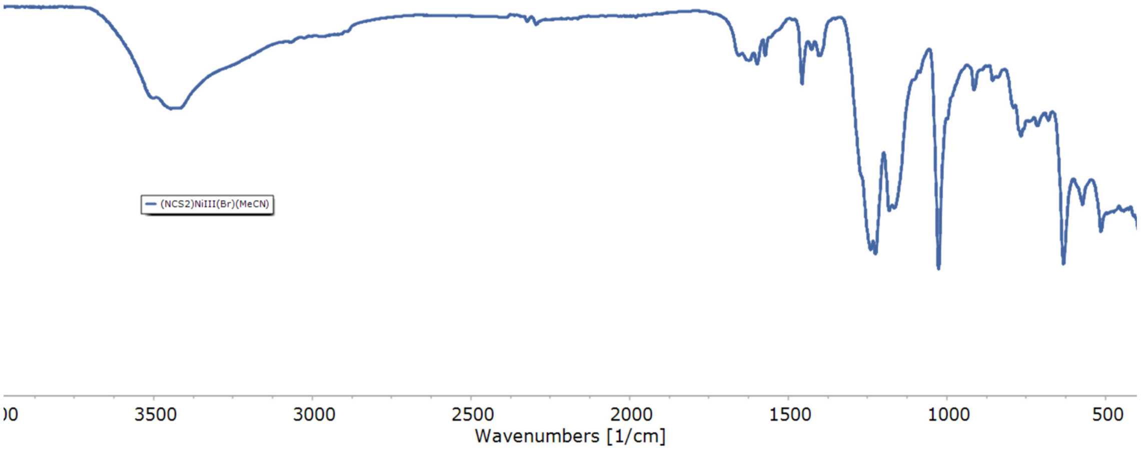


**Figure S80.** IR spectrum of **[2-Br]**$^{+}$

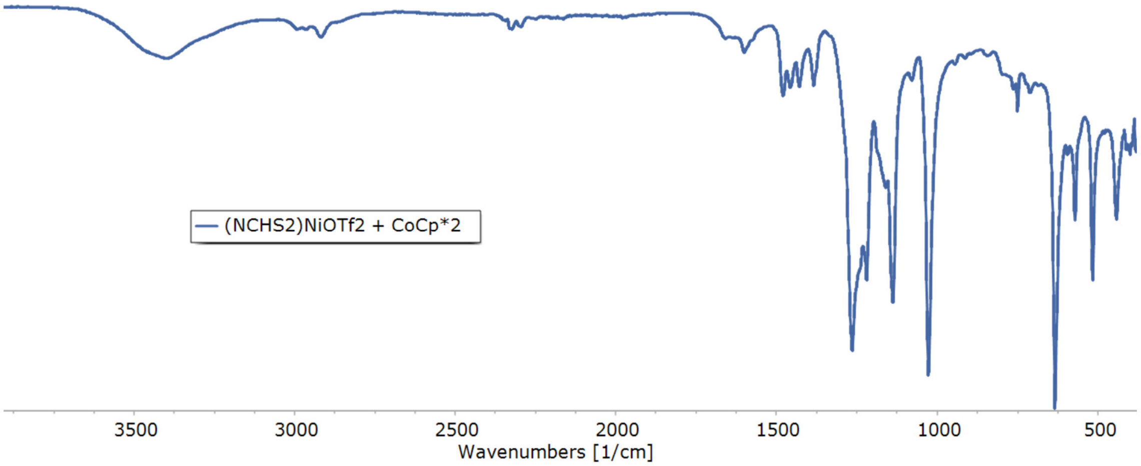


**Figure S81.** IR spectrum of solid crashed out after adding $CoCp*_2$ to **1.** The presence of a nitrile stretch in the IR, coupled with EPR spectroscopic evidence led us to propose a four-coordinate structure for **3**.

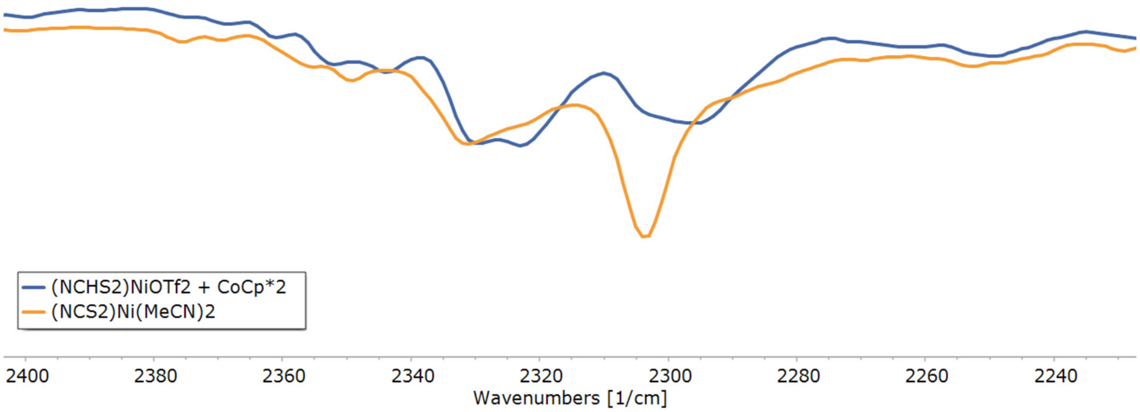


**Figure S82.** Overlaid IR spectra of [**1** + CoCp*$_2$] and **2**. There is a shift in $\nu_{CN}$ to lower wavenumbers (2303 cm$^{-1}$ for **2** to 2297 cm$^{-1}$ for **1 + CoCp*$_2$**) which suggests a change in the oxidation state on reduction. The effect on CN stretch can be obfuscated by the fact that **2** has an electron rich Ni-C bond. Nonetheless, a shift to a lower wavenumber does suggest an increase in electron density at Ni.

## 9. ESI-MS analysis of the post-electrolysis solutions

<u>8.1. Electrolysis in $CH_3CN$.</u> Preparative scale electrolysis for **1** in MeCN electrolyte in the presence of 0.043 M of TFA and 1.5 M of $H_2O$ was carried out for one hour at the applied potential of $E_{cat}$ (–1.7 V) using carbon cloth as a working electrode, Pt-mesh as a counter electrode, and $Ag/AgNO_3$ reference electrode. The post-electrolysis solution was then treated with conc. HCl and extracted with dry $Et_2O$. The ether layer was filtered through a small silica plug and the collected solution was dried over $MgSO_4$. ESI-MS data collected for such ether layer showed a peak at m/z 274.0711 (calcd for $[NCHS2H]^+$, m/z 274.0672, Figure S39), confirming the existence of free ligand, NCHS2, in the ether layer. It is noteworthy that ESI-MS recorded for the post-CPE solution after extracting in $Et_2O$ without doing any acid treatment did not show any peak that corresponds to the free NCHS2 ligand, suggesting that the Ni complex is stable during the CPE experiment (Figure S40).

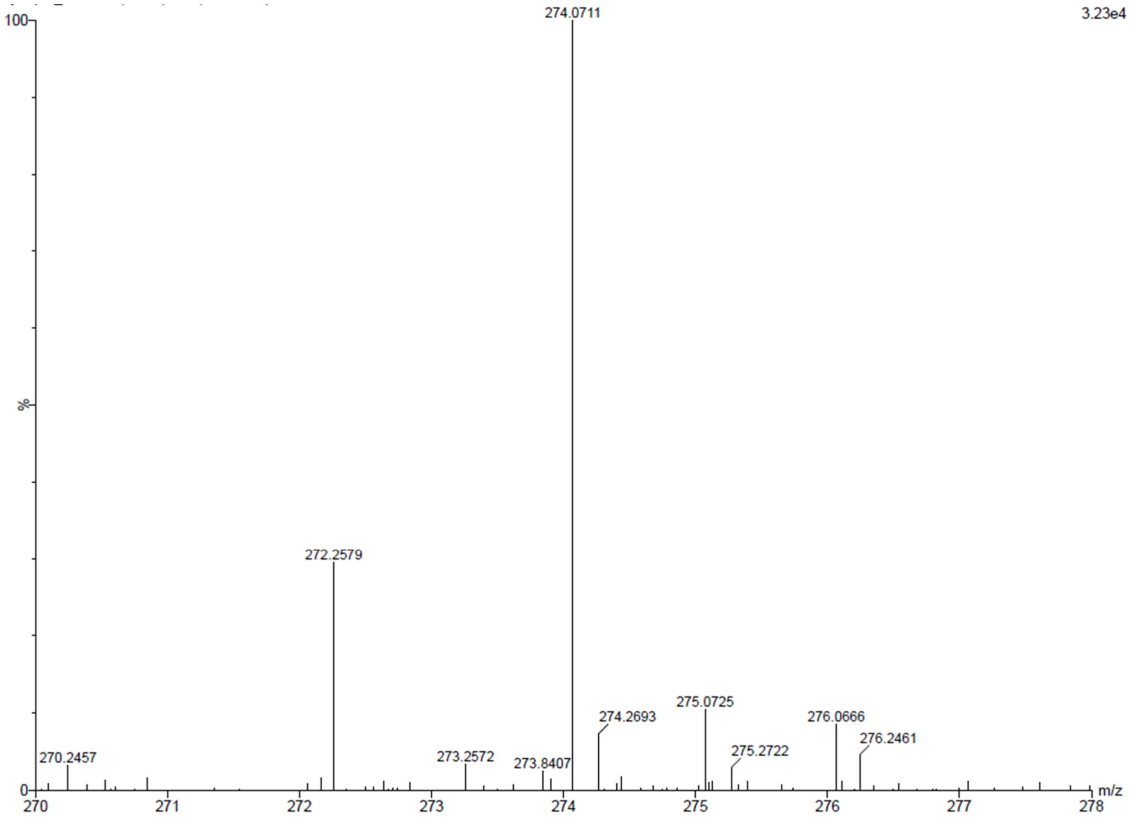


**Figure S83.** ESI-MS data for the post-electrolysis $CH_3CN$ solution after the HCl treatment, followed by ether extraction. The peak at m/z 274.0711 corresponds to the free ligand, NCHS2.

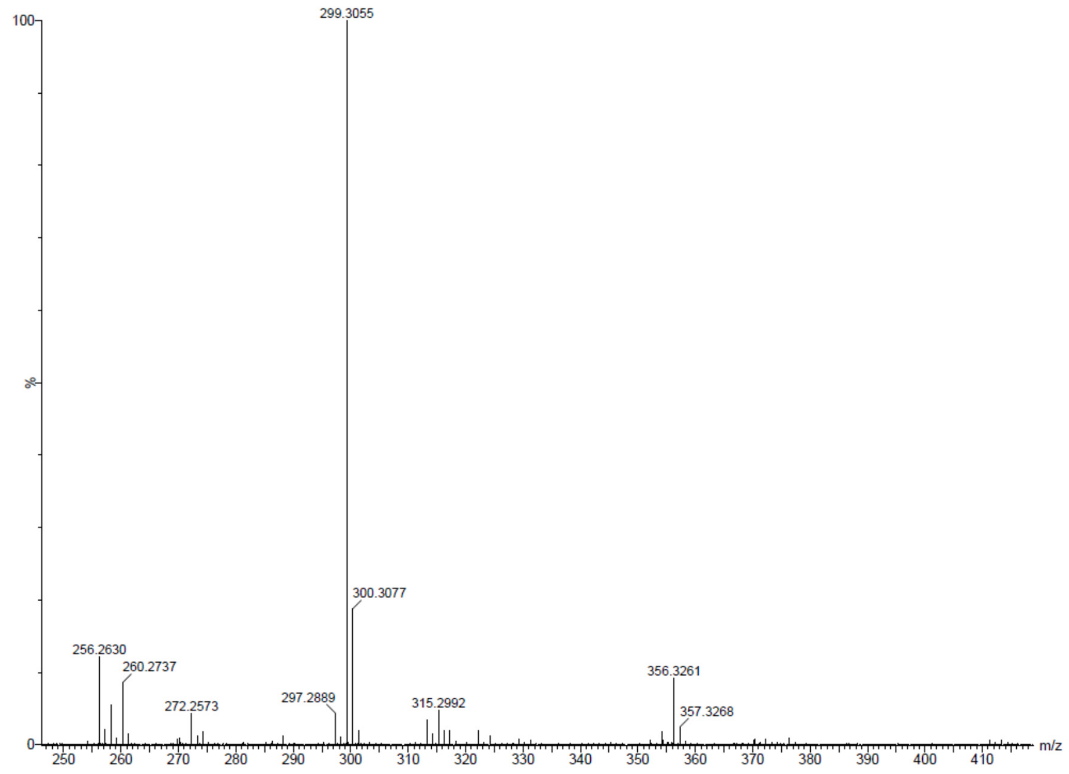


**Figure S84.** ESI-MS data for the post-electrolysis $CH_3CN$ solution extracted in $Et_2O$ without conc. HCl treatment. No such peak for the free NCHS2 ligand was observed.

8.2. Electrolysis in $CD_3CN$. The preparative scale electrolysis for **1** as described above was also repeated in $CD_3CN$ in the presence of 0.043 M of TFA and 1.5 M of $D_2O$. The post-electrolysis solution was then treated with 35% DCl in $D_2O$ and extracted using dry $Et_2O$. The ether layer was filtered through a small silica plug and the collected solution was dried over $MgSO_4$. ESI-MS data collected for such ether layer showed peaks at m/z 274.0756 and 275.0797 (calcd m/z for $[NCHS2H]^+$ and $[NCDS2H]^+$ is 274.0672 and 275.0787, respectively, Figure S41), confirming the existence of free ligand, NCHS2, as well as ~35% NCDS2 due to deuterium incorporation that has likely occurred via the activation of the $C_{ipso}$-H bond of NCHS2 ligand during electrolysis. Furthermore, the post-electrolysis solution after treating with 35% DCl in $D_2O$ was heated at 80°C for 30 mins. The resultant solution was then extracted in dry $Et_2O$ and the formation of NCDS2 in ~60% further supports the activation of the $C_{ipso}$-H bond of NCHS2 ligand during electrolysis, as was observed in the ESI-MS data (Figure S42).

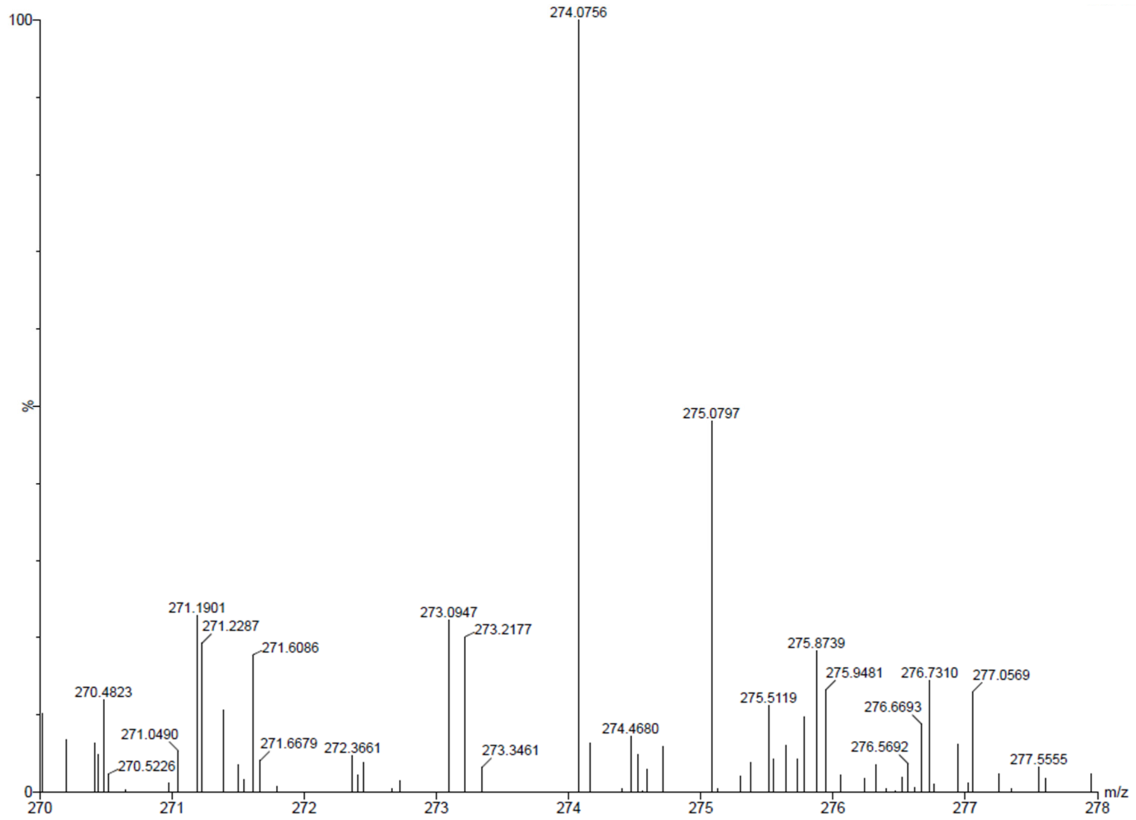


**Figure S85.** ESI-MS data for the post-electrolysis $CD_3CN$ solution upon treating with 35% DCl in $D_2O$, followed by ether extraction. The peak at m/z 274.0756 and 275.0797 correspond to NCHS2 and NCDS2, respectively.

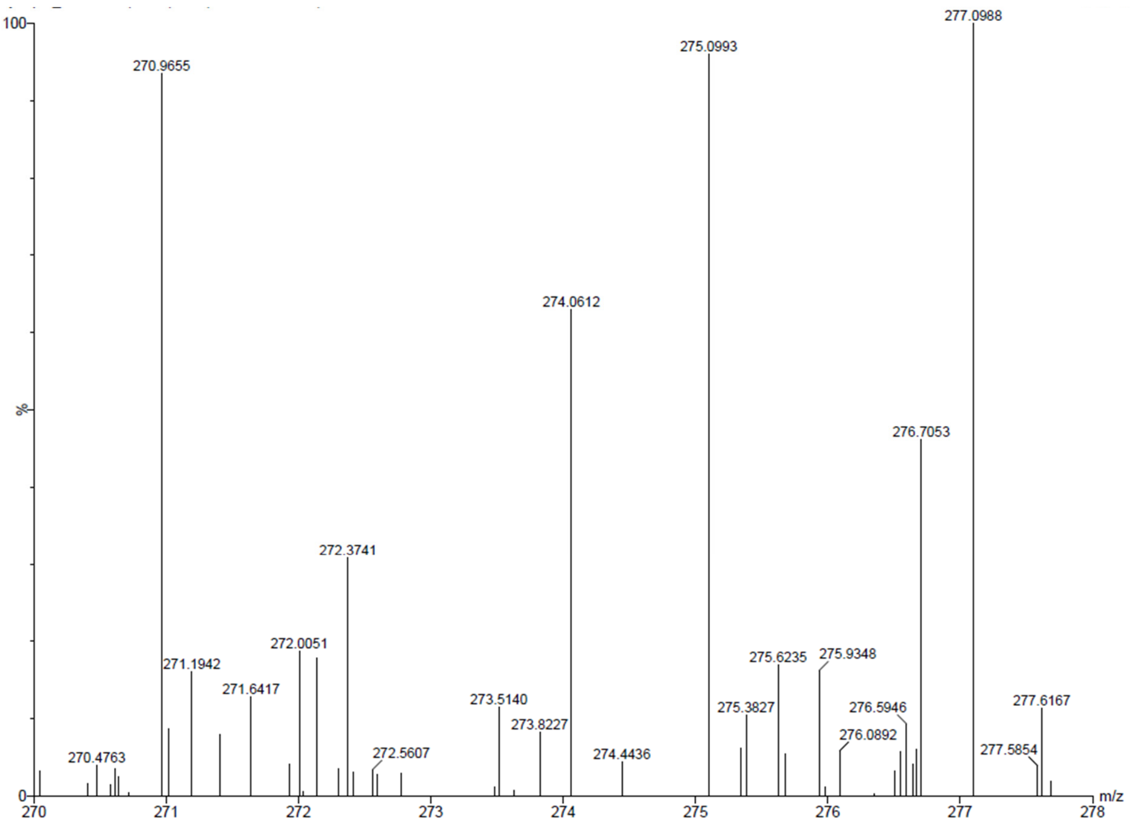


**Figure S86.** ESI-MS data for the post-electrolysis $CD_3CN$ solution upon treating with 35% DCl in $D_2O$, followed by heating at 80°C for 30 mins. The solution was then extracted in $Et_2O$ and ESI-MS showed peaks at m/z 274.0612 and 275.0993 that correspond to NCHS2 and NCDS2, respectively.

## 10. X-ray crystal structure characterization

*General information:* Suitable crystals of appropriate dimensions were mounted on Mitgen loops in random orientations. Preliminary examination and data collection were performed using a Bruker Kappa Apex-II Charge Coupled Device (CCD) Detector system single crystal X-ray diffractometer equipped with an Oxford Cryosteam LT device. Data were collected using graphite monochromated MO Kα radiation (λ = 0.71073 Å) from a fine focus sealed tube X-ray source. Preliminary unit cell constants were determined with a set of 36 narrow frame scans. Typical data sets consist of a combination of ϖ and φ scan frames with typical scan width of 0.5° and counting time of 15-30 seconds/frame at a crystal to detector distance of ~4.0 cm. The collected frames were integrated using an orientation matrix determined from the narrow frame scans. Apex II and SAINT software packages (*Bruker Analytical X-Ray, Madison, WI, 2008*) were used for data collection and data integration. Analysis of the integrated data did not show any decay. Final cell constants were determined by global refinement of reflections from the complete data set. Data were corrected for systematic errors using SADABS (*Bruker Analytical X-Ray, Madison, WI, 2008*) based on the Laue symmetry using equivalent reflections. Structure solutions and refinement were carried out using the SHELXTL- PLUS software package.[13] The structures were refined with full matrix least-squares refinement by minimizing $\Sigma w(Fo^2-Fc^2)^2$. All non-hydrogen atoms were refined anisotropically to convergence.
Typically, H atoms are added at the calculated positions in the final refinement cycles.

The deposition numbers CCDC 2053834 (NCHS2), 2053836 (**1**), 2053837 (**2-Br**), 2195927 ([**2**]$^+$) and 2195932 ([**2-Br**]$^+$) at the Cambridge Crystallographic Data Centre CCDC contain the supplementary crystallographic data. These data are provided free of charge by the Cambridge Crystallographic Data Centre. Crystallographic details are summarized in Tables S6–S14.

## X-ray structure determination of NCHS2

**Table S6.** Crystal data and structure refinement for NCHS2.

| | | |
|---|---|---|
| Identification code | l13116/lt/x8/Gina | |
| Empirical formula | $C_{15} H_{15} N S_2$ | |
| Formula weight | 273.40 | |
| Temperature | 100(2) K | |
| Wavelength | 0.71073 Å | |
| Crystal system | Monoclinic | |
| Space group | $P2_1/c$ | |
| Unit cell dimensions | a = 8.9461(5) Å | $\alpha$= 90°. |
| | b = 13.9735(7) Å | $\beta$= 101.958(3)°. |
| | c = 21.5657(11) Å | $\gamma$ = 90°. |
| Volume | 2637.4(2) $Å^3$ | |
| Z | 8 | |
| Density (calculated) | 1.377 $Mg/m^3$ | |
| Absorption coefficient | 0.384 $mm^{-1}$ | |
| F(000) | 1152 | |
| Crystal size | 0.519 x 0.417 x 0.166 $mm^3$ | |
| Theta range for data collection | 0.965 to 27.541°. | |
| Index ranges | $-11\leq h\leq 11$, $-17\leq k\leq 18$, $-28\leq l\leq 28$ | |
| Reflections collected | 48794 | |
| Independent reflections | 6206 [R(int) = 0.0476] | |
| Completeness to theta = 25.242° | 100.0 % | |
| Absorption correction | Semi-empirical from equivalents | |
| Max. and min. transmission | 0.8621 and 0.7583 | |
| Refinement method | Full-matrix least-squares on $F^2$ | |
| Data / restraints / parameters | 6206 / 0 / 326 | |
| Goodness-of-fit on $F^2$ | 1.070 | |
| Final R indices [I>2sigma(I)] | R1 = 0.0422, wR2 = 0.1093 | |
| R indices (all data) | R1 = 0.0453, wR2 = 0.1116 | |
| Largest diff. peak and hole | 1.183 and -0.337 e.$Å^{-3}$ | |

**Table S7.** Bond lengths [Å] and angles [°] for NCHS2 are included in Supplementary Data 1.

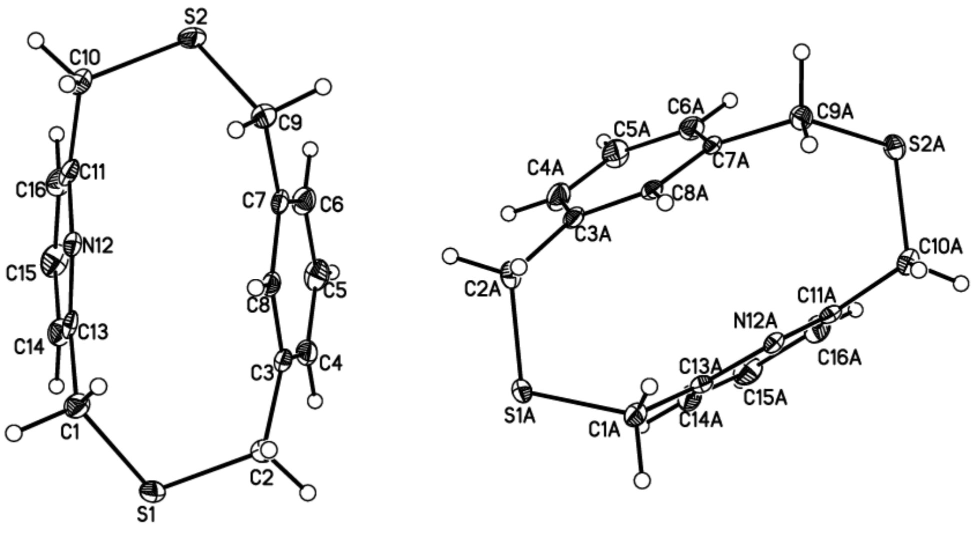


**Figure S87.** Projection view of NCHS2 with 50% probability ellipsoids.

**X-ray structure determination of (NCHS2)Ni(OTf)$_2$, 1**

**Table S8.** Crystal data and structure refinement for **1**

| | | |
|---|---|---|
| Identification code | l3317/lt/X8/1GT-109 | |
| Empirical formula | $C_{35} H_{32} Cl_2 F_{12} N_2 Ni_2 O_{12} S_8$ | |
| Formula weight | 1345.42 | |
| Temperature | 100(2) K | |
| Wavelength | 0.71073 Å | |
| Crystal system | Triclinic | |
| Space group | P-1 | |
| Unit cell dimensions | a = 8.5396(15) Å | $\alpha$= 110.541(8)°. |
| | b = 16.341(3) Å | $\beta$= 91.030(9)°. |
| | c = 18.902(3) Å | $\gamma$ = 103.948(8)°. |
| Volume | 2381.9(7) Å$^3$ | |
| Z | 2 | |
| Density (calculated) | 1.876 Mg/m$^3$ | |
| Absorption coefficient | 1.362 mm$^{-1}$ | |
| F(000) | 1356 | |
| Crystal size | 0.211 x 0.102 x 0.067 mm$^3$ | |
| Theta range for data collection | 1.380 to 26.446°. | |
| Index ranges | -10≤h≤9, -20≤k≤13, -17≤l≤23 | |
| Reflections collected | 25609 | |
| Independent reflections | 9797 [R(int) = 0.1202] | |
| Completeness to theta = 25.242° | 99.8 % | |
| Absorption correction | Semi-empirical from equivalents | |
| Max. and min. transmission | 0.8013 and 0.6827 | |
| Refinement method | Full-matrix least-squares on F$^2$ | |
| Data / restraints / parameters | 9797 / 1 / 664 | |
| Goodness-of-fit on F$^2$ | 1.012 | |
| Final R indices [I>2sigma(I)] | R1 = 0.0705, wR2 = 0.1208 | |
| R indices (all data) | R1 = 0.1695, wR2 = 0.1567 | |
| Largest diff. peak and hole | 0.783 and -0.711 e.Å$^{-3}$ | |

**Table S9.** Bond lengths [Å] and angles [°] for **1** are included in Supplementary Data 2.

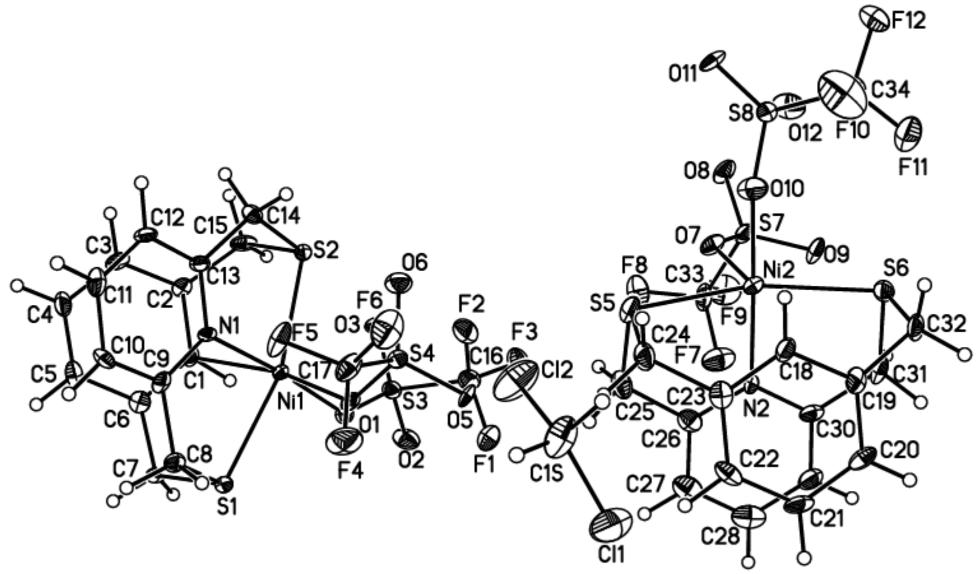


**Figure S88.** Projection view of **1(OTf)**2 with 50% probability ellipsoids.

**X-ray structure determination of [(NCS2)Ni($\mu$-Br)]$_2$, 2-Br**

**Table S10.** Crystal data and structure refinement for [(NCS2)Ni($\mu$-Br)]$_2$

| | |
|---|---|
| Chemical formula | $C_{15}H_{14}BrNNiS_2$ |
| $M_r$ | 411.01 |
| Crystal system, space group | Monoclinic, *I*2/*a* |
| Temperature (K) | 100 |
| *a*, *b*, *c* (Å) | 14.0350 (2), 12.8399 (2), 16.3513 (3) |
| b (°) | 95.27 |
| *V* (Å$^3$) | 2934.20 (8) |
| *Z* | 8 |
| Radiation type | Mo *K*a |
| m (mm$^{-1}$) | 4.32 |
| Crystal size (mm) | 0.31 × 0.09 × 0.04 |
| Data collection | |
| Diffractometer | Bruker *APEX*-II CCD |
| Absorption correction | Multi-scan<br>*SADABS2016*/2 (Bruker,2016/2) was used for absorption correction. wR2(int) was 0.1165 before and 0.0478 after correction. The Ratio of minimum to maximum transmission is 0.7617. The l/2 correction factor is Not present. |
| $T_{min}$, $T_{max}$ | 0.568, 0.746 |
| No. of measured, independent and observed [$I$ > 2s($I$)] reflections | 104967, 3647, 3541 |
| $R_{int}$ | 0.032 |
| (sin q/l)$_{max}$ (Å$^{-1}$) | 0.667 |
| Refinement | |
| $R[F^2 > 2s(F^2)]$, $wR(F^2)$, $S$ | 0.016, 0.042, 1.10 |
| No. of reflections | 3647 |
| No. of parameters | 181 |
| H-atom treatment | H-atom parameters constrained |
| D$\rho_{max}$, D$\rho_{min}$ (e Å$^{-3}$) | 0.58, -0.28 |

Computer programs: *SAINT* V8.38A (Bruker, 2018), XL (Sheldrick, 2008), Olex2 1.3 (Dolomanov *et al.*, 2009).

**Table S11.** Bond lengths [Å] and angles [°] for [(NCS2)Ni($\mu$-Br)]$_2$

| | |
|---|---|
| Br1—Ni1 | 2.5487 (2) |
| Br1—Ni1$^{i}$ | 2.7200 (2) |
| Ni1—Br1$^{i}$ | 2.7199 (2) |
| Ni1—S1 | 2.3758 (4) |
| Ni1—S2 | 2.3898 (4) |
| Ni1—N1 | 2.0423 (11) |
| Ni1—C1 | 1.9798 (13) |
| S1—C8 | 1.8221 (14) |
| S1—C7 | 1.8235 (14) |
| S2—C15 | 1.8292 (16) |
| S2—C14 | 1.8093 (15) |
| N1—C13 | 1.3427 (17) |
| N1—C9 | 1.3477 (17) |
| C1—C2 | 1.3987 (18) |
| C1—C6 | 1.4037 (19) |
| C10—H10 | 0.9500 |
| C10—C9 | 1.3895 (18) |
| C10—C11 | 1.386 (2) |
| C5—H5 | 0.9500 |
| C5—C6 | 1.3977 (19) |
| C5—C4 | 1.398 (2) |
| C13—C12 | 1.3894 (19) |
| C13—C14 | 1.5099 (19) |
| C2—C3 | 1.400 (2) |
| C2—C15 | 1.513 (2) |
| C9—C8 | 1.5120 (18) |
| C6—C7 | 1.5147 (19) |
| C11—H11 | 0.9500 |
| C11—C12 | 1.390 (2) |
| C12—H12 | 0.9500 |
| C4—H4 | 0.9500 |
| C4—C3 | 1.389 (2) |
| C3—H3 | 0.9500 |
| C8—H8A | 0.9900 |
| C8—H8B | 0.9900 |
| C15—H00A | 0.9900 |
| C15—H00B | 0.9900 |
| C14—H14A | 0.9900 |
| C14—H14B | 0.9900 |
| C7—H7A | 0.9900 |
| C7—H7B | 0.9900 |
| | |
| Ni1—Br1—Ni1$^{i}$ | 94.461 (6) |

| | |
|---|---|
| Br1—Ni1—Br1$^{i}$ | 85.539 (6) |
| S1—Ni1—Br1$^{i}$ | 95.467 (10) |
| S1—Ni1—Br1 | 91.133 (10) |
| S1—Ni1—S2 | 167.309 (14) |
| S2—Ni1—Br1$^{i}$ | 92.127 (11) |
| S2—Ni1—Br1 | 99.598 (10) |
| N1—Ni1—Br1 | 167.91 (3) |
| N1—Ni1—Br1$^{i}$ | 83.12 (3) |
| N1—Ni1—S1 | 85.80 (3) |
| N1—Ni1—S2 | 85.03 (3) |
| C1—Ni1—Br1 | 99.17 (4) |
| C1—Ni1—Br1$^{i}$ | 175.16 (4) |
| C1—Ni1—S1 | 85.63 (4) |
| C1—Ni1—S2 | 85.99 (4) |
| C1—Ni1—N1 | 92.27 (5) |
| C8—S1—Ni1 | 95.87 (4) |
| C8—S1—C7 | 100.53 (7) |
| C7—S1—Ni1 | 95.42 (5) |
| C15—S2—Ni1 | 97.93 (5) |
| C14—S2—Ni1 | 94.97 (5) |
| C14—S2—C15 | 103.15 (7) |
| C13—N1—Ni1 | 119.82 (9) |
| C13—N1—C9 | 119.74 (11) |
| C9—N1—Ni1 | 120.16 (9) |
| C2—C1—Ni1 | 121.13 (10) |
| C2—C1—C6 | 117.97 (12) |
| C6—C1—Ni1 | 120.29 (10) |
| C9—C10—H10 | 120.6 |
| C11—C10—H10 | 120.6 |
| C11—C10—C9 | 118.90 (12) |
| C6—C5—H5 | 120.4 |
| C6—C5—C4 | 119.22 (14) |
| C4—C5—H5 | 120.4 |
| N1—C13—C12 | 121.52 (12) |
| N1—C13—C14 | 118.73 (12) |
| C12—C13—C14 | 119.70 (12) |
| C1—C2—C3 | 120.87 (13) |
| C1—C2—C15 | 119.74 (13) |
| C3—C2—C15 | 119.39 (13) |
| N1—C9—C10 | 121.49 (12) |
| N1—C9—C8 | 118.17 (11) |
| C10—C9—C8 | 120.25 (12) |
| C1—C6—C7 | 117.35 (12) |
| C5—C6—C1 | 121.65 (13) |
| C5—C6—C7 | 120.99 (12) |

| | |
|---|---|
| C10—C11—H11 | 120.4 |
| C10—C11—C12 | 119.30 (12) |
| C12—C11—H11 | 120.4 |
| C13—C12—C11 | 118.91 (13) |
| C13—C12—H12 | 120.5 |
| C11—C12—H12 | 120.5 |
| C5—C4—H4 | 120.0 |
| C3—C4—C5 | 120.04 (13) |
| C3—C4—H4 | 120.0 |
| C2—C3—H3 | 119.9 |
| C4—C3—C2 | 120.23 (13) |
| C4—C3—H3 | 119.9 |
| S1—C8—H8A | 108.5 |
| S1—C8—H8B | 108.5 |
| C9—C8—S1 | 114.96 (9) |
| C9—C8—H8A | 108.5 |
| C9—C8—H8B | 108.5 |
| H8A—C8—H8B | 107.5 |
| S2—C15—H00A | 108.7 |
| S2—C15—H00B | 108.7 |
| C2—C15—S2 | 114.33 (10) |
| C2—C15—H00A | 108.7 |
| C2—C15—H00B | 108.7 |
| H00A—C15—H00B | 107.6 |
| S2—C14—H14A | 108.2 |
| S2—C14—H14B | 108.2 |
| C13—C14—S2 | 116.17 (10) |
| C13—C14—H14A | 108.2 |
| C13—C14—H14B | 108.2 |
| H14A—C14—H14B | 107.4 |
| S1—C7—H7A | 109.2 |
| S1—C7—H7B | 109.2 |
| C6—C7—S1 | 112.19 (9) |
| C6—C7—H7A | 109.2 |
| C6—C7—H7B | 109.2 |
| H7A—C7—H7B | 107.9 |

Symmetry code(s): (i) $-x+1/2$, $-y+1/2$, $-z+1/2$.

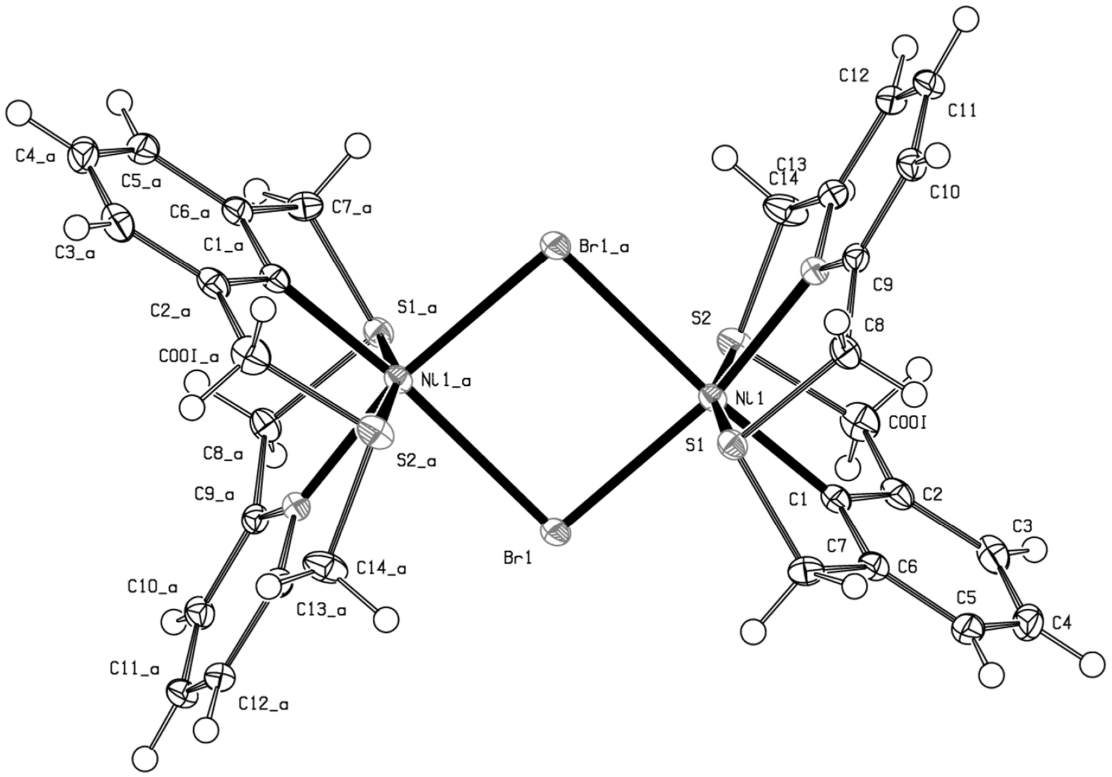


**Figure S89.** Projection view of [(NCS2)Ni($\mu$-Br)]$_2$ with 50% probability ellipsoids.

**X-ray structure determination of $[(NCS2)Ni(MeCN)]_2[SbF_6][OTf]$, $[2]^+$**

**Table S11.** Crystal data and structure refinement for [(NCS2)Ni(MeCN)]2[SbF6][OTf], [2]+

| | |
|---|---|
| Identification code | NCS2NiIII_t2 |
| Empirical formula | $C_{20.22}H_{20}F_{8.32}N_3NiO_{3.67}S_{3.22}Sb_{0.78}$ |
| Formula weight | 778.05 |
| Temperature/K | 130.0 |
| Crystal system | triclinic |
| Space group | P-1 |
| a/Å | 11.7318(3) |
| b/Å | 12.0951(3) |
| c/Å | 12.1897(3) |
| α/° | 114.6400(10) |
| β/° | 92.4590(10) |
| γ/° | 116.41 |
| Volume/Å$^3$ | 1351.58(6) |
| Z | 2 |
| $\rho_{calc}$g/cm$^3$ | 1.912 |
| μ/mm$^{-1}$ | 1.815 |
| F(000) | 771.0 |
| Crystal size/mm$^3$ | 0.338 × 0.255 × 0.205 |
| Radiation | MoKα (λ = 0.71073) |
| 2Θ range for data collection/° | 4.038 to 61.046 |
| Index ranges | $-16 \leq h \leq 16$, $-17 \leq k \leq 17$, $-17 \leq l \leq 17$ |
| Reflections collected | 115863 |
| Independent reflections | 8259 [$R_{int}$ = 0.0304, $R_{sigma}$ = 0.0127] |
| Data/restraints/parameters | 8259/769/562 |
| Goodness-of-fit on $F^2$ | 1.243 |
| Final R indexes [I>=2σ (I)] | $R_1$ = 0.0291, $wR_2$ = 0.0665 |
| Final R indexes [all data] | $R_1$ = 0.0311, $wR_2$ = 0.0672 |
| Largest diff. peak/hole / e Å$^{-3}$ | 0.55/-0.55 |

**Table S12.** Bond lengths [Å] and angles [°] for [(NCS2)Ni(MeCN)$_2$][SbF$_6$][OTf]

| | | |
|---|---|---|
| Ni1 | S1 | 2.3431(5) |
| Ni1 | S2 | 2.3556(5) |
| Ni1 | N2 | 1.9821(17) |
| Ni1 | N1 | 1.9885(17) |
| Ni1 | N3 | 1.9865(16) |
| Ni1 | C1 | 1.9651(17) |
| S1 | C11 | 1.809(2) |
| S1 | C12 | 1.814(2) |
| S2 | C18 | 1.809(2) |
| S2 | C19 | 1.818(2) |
| N2 | C4 | 1.138(3) |
| N1 | C2 | 1.130(3) |
| N3 | C13 | 1.361(2) |
| N3 | C17 | 1.365(2) |
| C1 | C10 | 1.376(3) |
| C1 | C6 | 1.371(3) |
| C13 | C12 | 1.497(3) |
| C13 | C14 | 1.395(3) |
| C10 | C11 | 1.502(3) |
| C10 | C9 | 1.388(3) |
| C6 | C7 | 1.399(3) |
| C6 | C19 | 1.501(3) |
| C4 | C5 | 1.450(3) |
| C17 | C18 | 1.504(3) |
| C17 | C16 | 1.389(3) |
| C9 | C8 | 1.391(3) |
| C2 | C3 | 1.457(3) |
| C7 | C8 | 1.381(3) |
| C14 | C15 | 1.380(3) |
| C16 | C15 | 1.377(3) |
| S3 | O1 | 1.442(2) |
| S3 | O2 | 1.443(3) |
| S3 | O3 | 1.436(2) |
| S3 | C20 | 1.817(3) |
| C20 | F1 | 1.323(3) |
| C20 | F2 | 1.337(3) |
| C20 | F3 | 1.331(3) |
| Sb1 | F4 | 1.867(10) |
| Sb1 | F5 | 1.884(10) |
| Sb1 | F6 | 1.877(10) |
| Sb1 | F7 | 1.881(10) |
| Sb1 | F8 | 1.870(10) |
| Sb1 | F9 | 1.892(10) |

| | | |
|---|---|---|
| Sb2 | F10 | 1.872(4) |
| Sb2 | F11 | 1.885(3) |
| Sb2 | F12 | 1.885(4) |
| Sb2 | F13 | 1.884(3) |
| Sb2 | F14 | 1.871(4) |
| Sb2 | F15 | 1.891(3) |
| S4 | O4 | 1.443(10) |
| S4 | O5 | 1.466(10) |
| S4 | O6 | 1.441(10) |
| S4 | C21 | 1.828(9) |
| C21 | F16 | 1.314(10) |
| C21 | F17 | 1.346(10) |
| C21 | F18 | 1.337(10) |
| S4A | O4A | 1.440(10) |
| S4A | O5A | 1.466(9) |
| S4A | O6A | 1.448(9) |
| S4A | C21A | 1.824(9) |
| C21A | F16A | 1.316(10) |
| C21A | F17A | 1.351(9) |
| C21A | F18A | 1.334(9) |

| | | | |
|---|---|---|---|
| S1 | Ni1 | S2 | 169.10(2) |
| N2 | Ni1 | S1 | 94.07(5) |
| N2 | Ni1 | S2 | 93.20(5) |
| N2 | Ni1 | N1 | 89.40(7) |
| N2 | Ni1 | N3 | 92.29(7) |
| N1 | Ni1 | S1 | 91.46(5) |
| N1 | Ni1 | S2 | 96.73(5) |
| N3 | Ni1 | S1 | 85.74(5) |
| N3 | Ni1 | S2 | 85.86(5) |
| N3 | Ni1 | N1 | 176.82(7) |
| C1 | Ni1 | S1 | 86.39(5) |
| C1 | Ni1 | S2 | 86.54(5) |
| C1 | Ni1 | N2 | 178.57(7) |
| C1 | Ni1 | N1 | 89.23(7) |
| C1 | Ni1 | N3 | 89.10(7) |
| C11 | S1 | Ni1 | 93.86(6) |
| C11 | S1 | C12 | 101.43(10) |
| C12 | S1 | Ni1 | 97.77(7) |
| C18 | S2 | Ni1 | 94.30(7) |
| C18 | S2 | C19 | 101.24(11) |
| C19 | S2 | Ni1 | 97.29(8) |
| C4 | N2 | Ni1 | 168.13(17) |
| C2 | N1 | Ni1 | 175.93(18) |

| | | | |
|---|---|---|---|
| C13 | N3 | Ni1 | 120.27(13) |
| F1 | C20 | F2 | 107.7(2) |
| F1 | C20 | F3 | 107.0(2) |
| F2 | C20 | S3 | 111.60(17) |
| F3 | C20 | S3 | 111.57(17) |
| F3 | C20 | F2 | 107.6(2) |
| F4 | Sb1 | F5 | 89.2(12) |
| F4 | Sb1 | F6 | 92.5(12) |
| F4 | Sb1 | F7 | 89.2(13) |
| F4 | Sb1 | F8 | 91.4(12) |
| F4 | Sb1 | F9 | 177.1(14) |
| F5 | Sb1 | F9 | 89.8(12) |
| F6 | Sb1 | F5 | 90.7(11) |
| F6 | Sb1 | F7 | 89.8(13) |
| F6 | Sb1 | F9 | 90.2(11) |
| F7 | Sb1 | F5 | 178.3(14) |
| F7 | Sb1 | F9 | 91.8(13) |
| F8 | Sb1 | F5 | 90.6(11) |
| F8 | Sb1 | F6 | 175.9(11) |
| F8 | Sb1 | F7 | 89.1(13) |
| F8 | Sb1 | F9 | 85.9(11) |
| F10 | Sb2 | F11 | 89.6(2) |
| F10 | Sb2 | F12 | 90.2(3) |
| F10 | Sb2 | F13 | 88.1(2) |
| F10 | Sb2 | F15 | 179.1(2) |
| C17 | C18 | S2 | 112.75(14) |
| C15 | C14 | C13 | 119.7(2) |
| C15 | C16 | C17 | 119.8(2) |
| C6 | C19 | S2 | 112.80(15) |
| C16 | C15 | C14 | 120.1(2) |
| O1 | S3 | O2 | 115.08(15) |
| O1 | S3 | C20 | 103.05(12) |
| O2 | S3 | C20 | 103.96(17) |
| O3 | S3 | O1 | 114.74(18) |
| O3 | S3 | O2 | 114.36(19) |
| O3 | S3 | C20 | 103.48(14) |
| F1 | C20 | S3 | 111.15(19) |
| F11 | Sb2 | F12 | 91.45(18) |
| F11 | Sb2 | F15 | 89.55(16) |
| F12 | Sb2 | F15 | 89.90(19) |
| F13 | Sb2 | F11 | 177.63(14) |
| F13 | Sb2 | F12 | 88.1(2) |
| F13 | Sb2 | F15 | 92.79(15) |
| F14 | Sb2 | F10 | 92.0(4) |
| F14 | Sb2 | F11 | 91.59(17) |

| | | | |
|---|---|---|---|
| F14 | Sb2 | F12 | 176.2(2) |
| F14 | Sb2 | F13 | 88.90(18) |
| F14 | Sb2 | F15 | 87.9(2) |
| O4 | S4 | O5 | 119.4(15) |
| O4 | S4 | C21 | 100.1(15) |
| O5 | S4 | C21 | 94.2(12) |
| O6 | S4 | O4 | 122.8(16) |
| O6 | S4 | O5 | 112.9(13) |
| O6 | S4 | C21 | 97.6(13) |
| F16 | C21 | S4 | 110.4(15) |
| F16 | C21 | F17 | 109.4(19) |
| F16 | C21 | F18 | 111(2) |
| F17 | C21 | S4 | 106.0(13) |
| F18 | C21 | S4 | 110.1(13) |
| F18 | C21 | F17 | 109.9(17) |
| O4A | S4A | O5A | 114.8(15) |
| O4A | S4A | O6A | 122.3(14) |
| O4A | S4A | C21A | 101.4(15) |
| O5A | S4A | C21A | 96.2(8) |
| O6A | S4A | O5A | 115.1(10) |
| O6A | S4A | C21A | 100.2(9) |
| F16A | C21A | S4A | 111.4(9) |
| F16A | C21A | F17A | 106.5(12) |
| F16A | C21A | F18A | 108.3(13) |
| F17A | C21A | S4A | 106.2(8) |
| F18A | C21A | S4A | 109.7(8) |
| F18A | C21A | F17A | 114.6(11) |

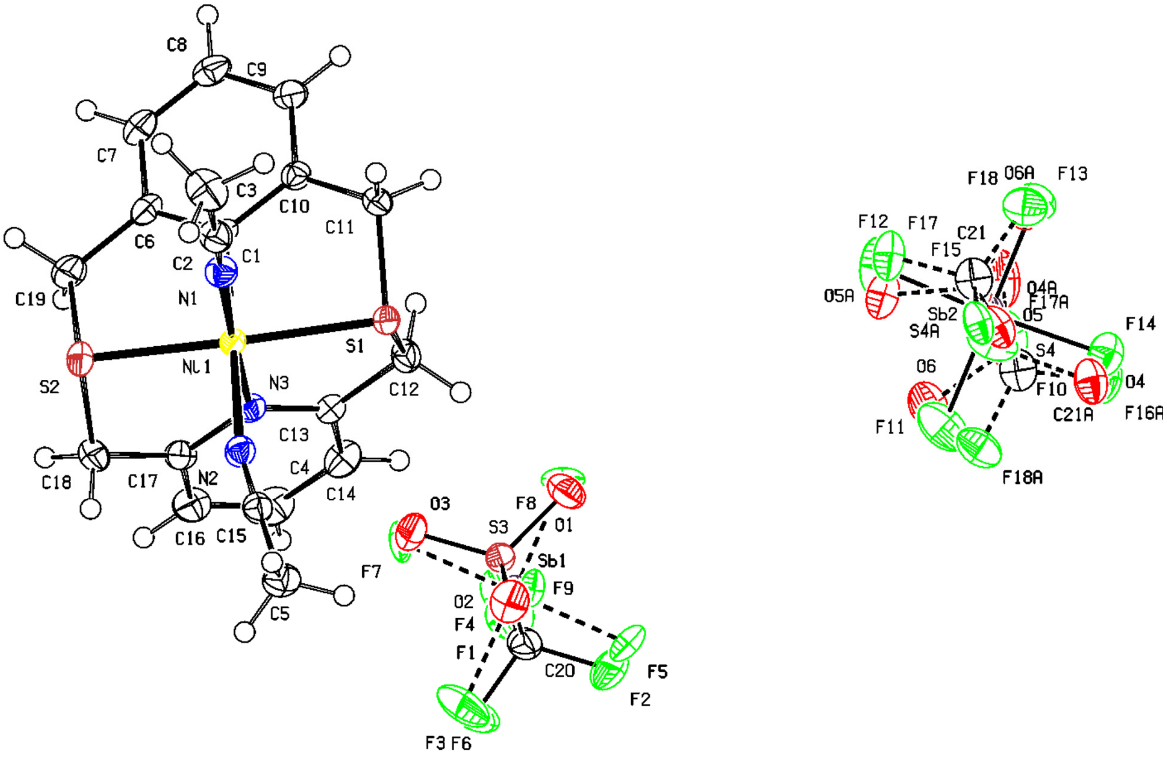


**Figure S90.** Projection view of [(NCS2)Ni(MeCN)2][SbF6][OTf] with 50% probability ellipsoids.

Calculated and reported formulae differ because there is partial occupation of the counteranion sites which have been modeled as above. There is also some disorder between C1 and N3 positions which has not been modeled as data/parameter ratio is low.

## X-ray structure determination of [(NCS2)Ni(MeCN)(Br)][OTf], [2-Br]$^{+}$

**Table S13.** Crystal data and structure refinement for [(NCS2)Ni(MeCN)(Br)][OTf]

| | |
|---|---|
| Identification code | ed66h (1) |
| Empirical formula | $C_{18}H_{17}BrF_3N_2NiO_3S_3$ |
| Formula weight | 601.13 |
| Temperature/K | 100.0 |
| Crystal system | monoclinic |
| Space group | $P2_1/n$ |
| a/Å | 21.9643(7) |
| b/Å | 8.7254(3) |
| c/Å | 22.0103(7) |
| α/° | 90 |
| β/° | 94.0010(10) |
| γ/° | 90 |
| Volume/Å$^3$ | 4207.9(2) |
| Z | 8 |
| $\rho_{calc}$g/cm$^3$ | 1.898 |
| μ/mm$^{-1}$ | 3.170 |
| F(000) | 2408.0 |
| Crystal size/mm$^3$ | 0.461 × 0.431 × 0.14 |
| Radiation | MoKα (λ = 0.71073) |
| 2Θ range for data collection/° | 4.264 to 56.606 |
| Index ranges | $-29 \leq h \leq 29$, $-11 \leq k \leq 11$, $-29 \leq l \leq 29$ |
| Reflections collected | 139719 |
| Independent reflections | 10474 [$R_{int}$ = 0.0644, $R_{sigma}$ = 0.0288] |
| Data/restraints/parameters | 10474/0/562 |
| Goodness-of-fit on F$^2$ | 1.066 |
| Final R indexes [I>=2σ (I)] | $R_1$ = 0.0419, $wR_2$ = 0.1041 |
| Final R indexes [all data] | $R_1$ = 0.0452, $wR_2$ = 0.1068 |
| Largest diff. peak/hole / e Å$^{-3}$ | 1.86/-1.28 |

**Table S14.** Bond lengths [Å] and angles [°] for [(NCS2)Ni(Br)(MeCN)][OTf]

| | | |
|---|---|---|
| Br1 | Ni1 | 2.4268(7) |
| Ni1 | S1 | 2.3571(11) |
| Ni1 | S2 | 2.3578(11) |
| Ni1 | N1 | 2.024(4) |
| Ni1 | N2 | 1.988(4) |
| Ni1 | C1 | 1.939(4) |
| S1 | C7 | 1.818(5) |
| S1 | C8 | 1.808(5) |
| S2 | C14 | 1.813(5) |
| S2 | C15 | 1.808(4) |
| N1 | C9 | 1.355(5) |
| N1 | C13 | 1.342(5) |
| N2 | C16 | 1.139(6) |
| C1 | C2 | 1.394(6) |
| C1 | C6 | 1.385(6) |
| C2 | C3 | 1.384(6) |
| C2 | C15 | 1.501(6) |
| C3 | C4 | 1.387(7) |
| C4 | C5 | 1.388(8) |
| C5 | C6 | 1.404(6) |
| C6 | C7 | 1.504(6) |
| C8 | C9 | 1.507(6) |
| C9 | C10 | 1.385(6) |
| C10 | C11 | 1.390(6) |
| C11 | C12 | 1.390(6) |
| C12 | C13 | 1.397(6) |
| C13 | C14 | 1.513(6) |
| C16 | C17 | 1.451(7) |
| Br2 | Ni2 | 2.4206(7) |
| Ni2 | S3 | 2.3616(12) |
| Ni2 | S4 | 2.3617(12) |
| Ni2 | N3 | 2.019(4) |
| Ni2 | N4 | 1.995(4) |
| Ni2 | C18 | 1.936(4) |
| S3 | C24 | 1.811(5) |
| S3 | C25 | 1.815(5) |
| S4 | C31 | 1.811(5) |
| S4 | C32 | 1.815(5) |
| N3 | C26 | 1.349(5) |
| N3 | C30 | 1.361(5) |
| N4 | C33 | 1.125(7) |
| C18 | C19 | 1.379(6) |
| C18 | C23 | 1.400(6) |

| | | |
|---|---|---|
| C19 | C20 | 1.416(6) |
| C19 | C32 | 1.490(6) |
| C20 | C21 | 1.380(7) |
| C21 | C22 | 1.385(7) |
| C22 | C23 | 1.405(6) |
| C23 | C24 | 1.494(6) |
| C25 | C26 | 1.507(6) |
| C26 | C27 | 1.389(6) |
| C27 | C28 | 1.388(6) |
| C28 | C29 | 1.384(6) |
| C29 | C30 | 1.397(6) |
| C30 | C31 | 1.503(6) |
| C33 | C34 | 1.472(7) |
| S5 | O1 | 1.428(7) |
| S5 | O2 | 1.423(5) |
| S5 | O3 | 1.435(4) |
| S5 | C35 | 1.781(5) |
| F1 | C35 | 1.330(7) |
| F2 | C35 | 1.346(9) |
| F3 | C35 | 1.324(7) |
| S6 | O4 | 1.440(4) |
| S6 | O5 | 1.471(6) |
| S6 | O6 | 1.410(5) |
| S6 | C36 | 1.807(10) |
| F4 | C36 | 1.406(12) |
| F5 | C36 | 1.327(10) |
| F6 | C36 | 1.296(9) |

| | | | |
|---|---|---|---|
| S1 | Ni1 | Br1 | 94.57(3) |
| S1 | Ni1 | S2 | 170.35(5) |
| S2 | Ni1 | Br1 | 93.07(3) |
| N1 | Ni1 | Br1 | 174.61(11) |
| N1 | Ni1 | S1 | 86.09(10) |
| N1 | Ni1 | S2 | 85.75(10) |
| N2 | Ni1 | Br1 | 95.76(12) |
| N2 | Ni1 | S1 | 91.31(12) |
| N2 | Ni1 | S2 | 93.77(12) |
| N2 | Ni1 | N1 | 89.57(16) |
| C1 | Ni1 | Br1 | 85.91(13) |
| C1 | Ni1 | S1 | 87.43(13) |
| C1 | Ni1 | S2 | 87.26(13) |
| C1 | Ni1 | N1 | 88.78(17) |
| C1 | Ni1 | N2 | 177.99(18) |
| C7 | S1 | Ni1 | 97.87(15) |
| C8 | S1 | Ni1 | 95.78(15) |

| | | | |
|---|---|---|---|
| C18 | Ni2 | S4 | 87.25(12) |
| C18 | Ni2 | N3 | 87.44(17) |
| C18 | Ni2 | N4 | 176.65(17) |
| C24 | S3 | Ni2 | 96.71(14) |
| C24 | S3 | C25 | 102.9(2) |
| C25 | S3 | Ni2 | 96.56(15) |
| C31 | S4 | Ni2 | 97.38(14) |
| C31 | S4 | C32 | 103.6(2) |
| C32 | S4 | Ni2 | 95.98(15) |
| C26 | N3 | Ni2 | 120.3(3) |
| C26 | N3 | C30 | 119.3(4) |
| C30 | N3 | Ni2 | 120.3(3) |
| C33 | N4 | Ni2 | 169.9(4) |
| C19 | C18 | Ni2 | 119.0(3) |
| C19 | C18 | C23 | 122.2(4) |
| C23 | C18 | Ni2 | 118.7(3) |
| C18 | C19 | C20 | 118.5(4) |
| C8 | S1 | C7 | 102.7(2) |
| C14 | S2 | Ni1 | 97.90(14) |
| C15 | S2 | Ni1 | 95.01(15) |
| C15 | S2 | C14 | 100.9(2) |
| C9 | N1 | Ni1 | 119.4(3) |
| C13 | N1 | Ni1 | 120.8(3) |
| C13 | N1 | C9 | 119.8(4) |
| C16 | N2 | Ni1 | 173.2(4) |
| C2 | C1 | Ni1 | 118.5(3) |
| C6 | C1 | Ni1 | 119.7(3) |
| C6 | C1 | C2 | 121.7(4) |
| C1 | C2 | C15 | 119.6(4) |
| C3 | C2 | C1 | 118.7(4) |
| C3 | C2 | C15 | 121.5(4) |
| C2 | C3 | C4 | 120.6(4) |
| C3 | C4 | C5 | 120.3(4) |
| C4 | C5 | C6 | 119.9(4) |
| C1 | C6 | C5 | 118.7(4) |
| C1 | C6 | C7 | 121.7(4) |
| C5 | C6 | C7 | 119.5(4) |
| C6 | C7 | S1 | 112.9(3) |
| C9 | C8 | S1 | 115.1(3) |
| N1 | C9 | C8 | 119.0(4) |
| N1 | C9 | C10 | 121.1(4) |
| C10 | C9 | C8 | 119.8(4) |
| C9 | C10 | C11 | 119.7(4) |
| C12 | C11 | C10 | 118.7(4) |

| | | | |
|---|---|---|---|
| C18 | C19 | C32 | 121.0(4) |
| C20 | C19 | C32 | 120.4(4) |
| C21 | C20 | C19 | 119.9(4) |
| C20 | C21 | C22 | 121.1(4) |
| C21 | C22 | C23 | 120.0(5) |
| C18 | C23 | C22 | 118.3(4) |
| C18 | C23 | C24 | 120.9(4) |
| C22 | C23 | C24 | 120.7(4) |
| C23 | C24 | S3 | 113.3(3) |
| C26 | C25 | S3 | 115.5(3) |
| N3 | C26 | C25 | 119.2(4) |
| N3 | C26 | C27 | 121.7(4) |
| C27 | C26 | C25 | 119.1(4) |
| C28 | C27 | C26 | 119.2(4) |
| C29 | C28 | C27 | 119.4(4) |
| C28 | C29 | C30 | 119.2(4) |
| N3 | C30 | C29 | 121.2(4) |
| N3 | C30 | C31 | 119.5(4) |
| C29 | C30 | C31 | 119.2(4) |
| C30 | C31 | S4 | 115.8(3) |
| C19 | C32 | S4 | 112.0(3) |
| N4 | C33 | C34 | 179.2(6) |
| O1 | S5 | O3 | 115.1(4) |
| O1 | S5 | C35 | 102.4(5) |
| O2 | S5 | O1 | 113.1(4) |
| O2 | S5 | O3 | 116.4(3) |
| O2 | S5 | C35 | 105.4(3) |
| C11 | C12 | C13 | 119.1(4) |
| N1 | C13 | C12 | 121.5(4) |
| N1 | C13 | C14 | 120.0(4) |
| C12 | C13 | C14 | 118.5(4) |
| C13 | C14 | S2 | 114.9(3) |
| C2 | C15 | S2 | 111.9(3) |
| N2 | C16 | C17 | 179.1(6) |
| S3 | Ni2 | Br2 | 93.29(3) |
| S3 | Ni2 | S4 | 171.27(5) |
| S4 | Ni2 | Br2 | 93.54(3) |
| N3 | Ni2 | Br2 | 172.29(11) |
| N3 | Ni2 | S3 | 86.25(10) |
| N3 | Ni2 | S4 | 86.25(10) |
| N4 | Ni2 | Br2 | 98.29(12) |
| N4 | Ni2 | S3 | 93.02(12) |
| N4 | Ni2 | S4 | 91.37(12) |
| N4 | Ni2 | N3 | 89.42(16) |

| | | | |
|---|---|---|---|
| C18 | Ni2 | Br2 | 84.85(13) |
| C18 | Ni2 | S3 | 87.96(13) |
| O3 | S5 | C35 | 102.2(3) |
| F1 | C35 | S5 | 109.7(4) |
| F1 | C35 | F2 | 109.1(6) |
| F2 | C35 | S5 | 110.1(6) |
| F3 | C35 | S5 | 112.4(4) |
| F3 | C35 | F1 | 108.7(6) |
| F3 | C35 | F2 | 106.9(6) |
| O4 | S6 | O5 | 114.4(3) |
| O4 | S6 | C36 | 99.8(3) |
| O5 | S6 | C36 | 100.0(5) |
| O6 | S6 | O4 | 118.0(3) |
| O6 | S6 | O5 | 116.1(4) |
| O6 | S6 | C36 | 104.4(4) |
| F4 | C36 | S6 | 105.7(6) |
| F5 | C36 | S6 | 109.8(7) |
| F5 | C36 | F4 | 111.8(8) |
| F6 | C36 | S6 | 113.1(6) |
| F6 | C36 | F4 | 106.9(9) |
| F6 | C36 | F5 | 109.4(7) |

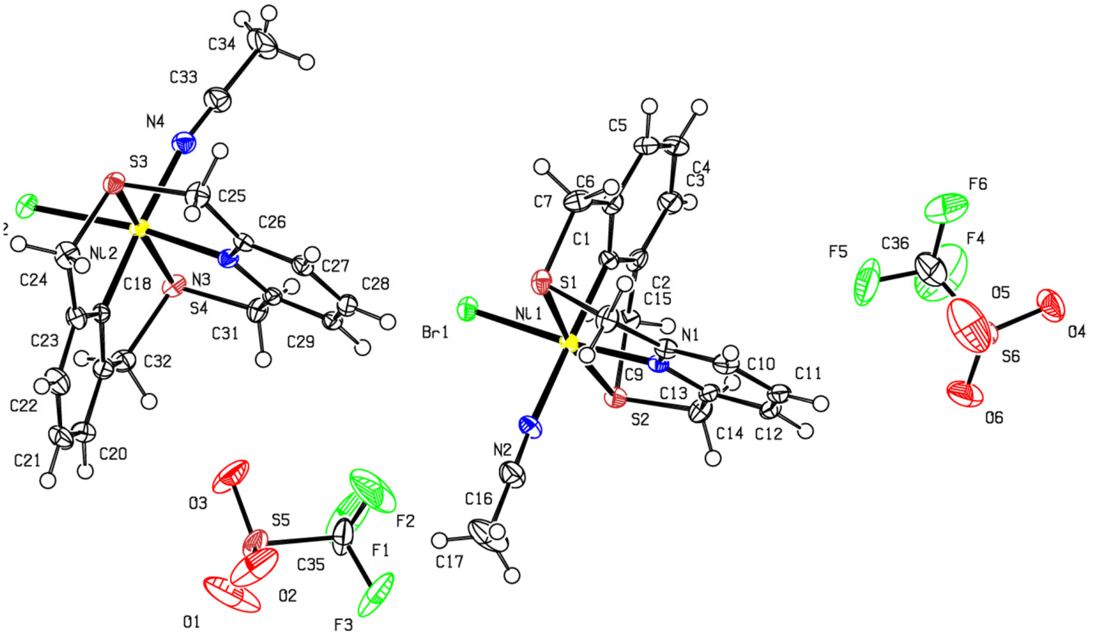


**Figure S91.** Projection view of [(NCS2)Ni(MeCN)(Br)][OTf] with 50% probability ellipsoids.

## 11. DFT Calculations

The density functional theory (DFT) calculations were performed using software package Gaussian 16.[14] The B3LYP[15,16] functional and tzvp[17,18] basis set was used for all atoms, as it was shown to work well for Ni complexes in our group previously[19]. Single points calculations were performed using the crystallographic coordinates for **1** and **[2]**$^{+}$. For calculating the energies of the electrochemical reaction pathway, the following considerations were used:

(a) All electron transfer reactions were calculated according to the following general reaction:

$$\text{Ox} + \text{Ferrocene} \rightarrow \text{Red} + \text{Ferrocenium}$$

Thus, the calculated potentials are relative to the $Fc^{+/0}$ couple, which is the internal standard used in the experiments.

(b) All proton transfer reactions were calculated according to the following reaction:

$$[A]^{n} + CF_3COOH \rightarrow [B\text{-}H]^{n+1} + CF_3COO^{-}$$

(c) The overall reaction can be represented as:

$$\text{catalyst} + 2H^{+} + 2e^{-} \rightarrow \text{catalyst} + H_2$$

Since the protons and electrons were added and hydrogen was released according to the energy diagram, the mass and charge balance was achieved by adding the energy of the reverse reaction. Thus, the energy of the following reaction was calculated to be 39.05 kcal/mol at the level of theory used –

$$2\ CF_3COOH + 2\ Fc \rightarrow 2\ CF_3COO^{-} + 2\ Fc^{+} + H_2$$

The atomic contributions to frontier molecular orbital were analyzed by the program Chemissian.[20]

(a) Frontier orbitals of [(NCHS2)Ni$^{I}$(MeCN)]$^{+}$, **3**

Various reports of electrocatalysts often cite ligand-centered redox as crucial steps in electrocatalysis. Hence, we analyzed the frontier orbitals of the Ni$^{I}$ state discussed in the main text and show that the paramagnetic states are unequivocally metal-centered.

**Table S14.** Atomic contributions of selected molecular orbitals for [(NCHS2)Ni$^{I}$(MeCN)]$^{+}$ (**3**).

| **Molecular Orbital** | **Contour Plots (Isovalue = 0.07)** | **Ni** | **S** | **Other** |
|---|---|---|---|---|
| | | | | |
| α-HOMO | 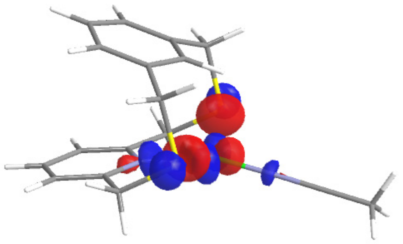 | 46% | 34% | - |
| β-HOMO | 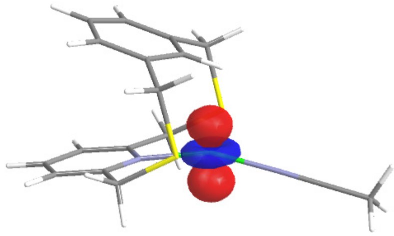 | 94% | - | 3% on ipso carbon of phenyl group |
| α-LUMO | 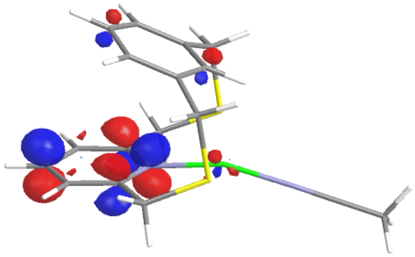 | 6% | 6% | Pyridine centered – 63% |
| β-LUMO | 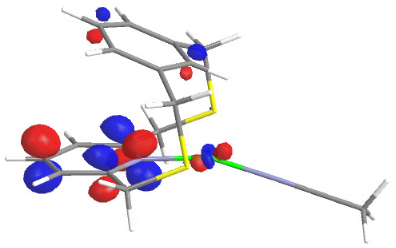 | 11% | 6% | Pyridine centered – 56% |

(b) DFT optimized coordinates of the intermediates discussed in the main text

**1. [(NCHS2)Ni$^{II}$(MeCN)]$^{2+}$**

| | | | |
|---|---|---|---|
| Ni | -0.889300 | -0.000000 | -0.676700 |
| S | -0.778600 | -2.268600 | -0.380100 |
| S | -0.778300 | 2.268600 | -0.380900 |
| N | 0.989700 | -0.000200 | -1.053100 |
| C | 0.899000 | -2.458000 | -1.098400 |
| H | 1.442900 | -3.209600 | -0.527600 |
| H | 0.731700 | -2.859500 | -2.100700 |
| C | 1.660100 | -1.171500 | -1.189500 |
| C | 3.019700 | -1.195500 | -1.458400 |
| H | 3.522500 | -2.147900 | -1.556000 |
| C | 3.708300 | -0.000600 | -1.593600 |
| H | 4.770900 | -0.000700 | -1.799800 |
| C | 3.019900 | 1.194500 | -1.458800 |
| H | 3.522900 | 2.146800 | -1.556800 |
| C | 1.660300 | 1.170800 | -1.189800 |
| C | 0.899600 | 2.457500 | -1.098900 |
| H | 0.732500 | 2.859100 | -2.101300 |
| H | 1.443600 | 3.209100 | -0.528200 |
| N | -2.763100 | 0.000100 | -0.547500 |
| C | -3.909700 | 0.000000 | -0.524700 |
| C | -5.349400 | -0.000100 | -0.491300 |
| H | -5.723800 | 0.892200 | -0.996200 |
| H | -5.723700 | -0.891500 | -0.998100 |
| H | -5.686900 | -0.001200 | 0.546600 |
| C | -0.462600 | 0.000400 | 1.994400 |
| H | -1.531800 | 0.000400 | 1.817500 |
| C | 0.220500 | 1.212700 | 2.033700 |
| C | 1.577100 | 1.210200 | 2.361900 |
| H | 2.124700 | 2.143700 | 2.412900 |
| C | 2.232500 | 0.000400 | 2.585400 |
| H | 3.281000 | 0.000400 | 2.856600 |
| C | 1.577000 | -1.209500 | 2.362300 |
| H | 2.124500 | -2.143000 | 2.413600 |
| C | 0.220400 | -1.212000 | 2.034100 |
| C | -0.482600 | -2.415000 | 1.488100 |
| H | -1.493100 | -2.533800 | 1.878600 |
| H | 0.067100 | -3.346100 | 1.614000 |
| C | -0.482400 | 2.415600 | 1.487300 |
| H | 0.067300 | 3.346700 | 1.613000 |
| H | -1.492900 | 2.534600 | 1.877700 |

**2. $[(NCHS2)Ni^{II}(MeCN)_2]^{2+}$, singlet**

| | | | |
|---|---|---|---|
| Ni | -0.837100 | -0.180300 | -0.000400 |
| S | -0.451600 | -0.461500 | -2.274500 |
| S | -0.453100 | -0.459000 | 2.274400 |
| N | 0.538600 | 1.158600 | -0.000600 |
| C | 0.435400 | 1.134700 | -2.454100 |
| H | 1.195200 | 1.036100 | -3.228200 |
| H | -0.321300 | 1.833400 | -2.819100 |
| C | 1.017100 | 1.643500 | -1.171300 |
| C | 1.989900 | 2.631300 | -1.195800 |
| H | 2.353400 | 2.991500 | -2.148400 |
| C | 2.481600 | 3.132600 | -0.001000 |
| H | 3.244900 | 3.900400 | -0.001200 |
| C | 1.989100 | 2.632700 | 1.193900 |
| H | 2.351800 | 2.993900 | 2.146400 |
| C | 1.016300 | 1.644800 | 1.169800 |
| C | 0.433500 | 1.137600 | 2.452800 |
| H | -0.323700 | 1.836600 | 2.816100 |
| H | 1.192500 | 1.040100 | 3.227800 |
| N | -2.179800 | -1.502500 | -0.000100 |
| C | -3.028900 | -2.272400 | 0.000100 |
| C | -4.098600 | -3.238400 | 0.000500 |
| H | -4.712200 | -3.099400 | 0.892200 |
| H | -4.712700 | -3.099500 | -0.891000 |
| H | -3.678400 | -4.245700 | 0.000400 |
| N | -2.488900 | 1.503400 | -0.001600 |
| C | -3.279900 | 2.338600 | -0.001100 |
| C | -4.273400 | 3.390700 | -0.000500 |
| H | -4.808400 | 3.392100 | -0.951400 |
| H | -4.983600 | 3.225800 | 0.811100 |
| H | -3.785300 | 4.356500 | 0.139400 |
| C | 1.232100 | -2.135200 | 0.001400 |
| H | 0.258400 | -2.609600 | 0.001400 |
| C | 1.808600 | -1.767800 | 1.213200 |
| C | 3.098000 | -1.232600 | 1.210700 |
| H | 3.571000 | -0.953400 | 2.144700 |
| C | 3.759400 | -1.025000 | 0.001500 |
| H | 4.766300 | -0.625900 | 0.001500 |
| C | 3.098700 | -1.234200 | -1.207900 |
| H | 3.572300 | -0.956100 | -2.142000 |
| C | 1.809300 | -1.769300 | -1.210500 |
| C | 0.925500 | -1.754800 | -2.415900 |

| | | | |
|---|---|---|---|
| H | 0.364300 | -2.680100 | -2.545200 |
| H | 1.446100 | -1.516900 | -3.342000 |
| C | 0.924100 | -1.751800 | 2.418100 |
| H | 1.444200 | -1.512800 | 3.344200 |
| H | 0.362900 | -2.677100 | 2.548200 |

**3. $[(NCHS2)Ni^{II}(MeCN)_2]^{2+}$, triplet**

| | | | |
|---|---|---|---|
| Ni | -0.895100 | 0.029700 | 0.000000 |
| S | -0.512500 | -0.210000 | -2.421900 |
| S | -0.512600 | -0.209800 | 2.421900 |
| N | 0.883600 | 1.085400 | -0.000100 |
| C | 0.595100 | -2.125800 | 0.000200 |
| H | -0.471700 | -2.330900 | 0.000200 |
| C | 1.267000 | -2.003800 | 1.218400 |
| C | 2.658000 | -1.919200 | 1.211000 |
| H | 3.199400 | -1.832100 | 2.145200 |
| C | 3.347400 | -1.921200 | 0.000100 |
| H | 4.429200 | -1.865000 | 0.000100 |
| C | 2.657900 | -1.919400 | -1.210800 |
| H | 3.199300 | -1.832500 | -2.145000 |
| C | 1.267000 | -2.004000 | -1.218100 |
| C | 0.463700 | -1.807300 | -2.468700 |
| H | -0.309300 | -2.566700 | -2.592300 |
| H | 1.077700 | -1.778000 | -3.366600 |
| C | 0.784900 | 1.089300 | -2.472100 |
| H | 1.520900 | 0.812200 | -3.225700 |
| H | 0.261100 | 1.973900 | -2.842500 |
| C | 1.462600 | 1.427700 | -1.165900 |
| C | 2.655600 | 2.139000 | -1.196300 |
| H | 3.102000 | 2.396000 | -2.147700 |
| C | 3.256700 | 2.500000 | -0.000100 |
| H | 4.188700 | 3.050900 | -0.000100 |
| C | 2.655500 | 2.139200 | 1.196100 |
| H | 3.101800 | 2.396200 | 2.147500 |
| C | 1.462500 | 1.427900 | 1.165700 |
| C | 0.784600 | 1.089800 | 2.471900 |
| H | 0.260500 | 1.974500 | 2.841800 |
| H | 1.520500 | 0.813200 | 3.225700 |
| C | 0.463800 | -1.806900 | 2.469000 |
| H | 1.077800 | -1.777400 | 3.366900 |
| H | -0.309200 | -2.566300 | 2.592800 |
| N | -2.600300 | -1.124300 | 0.000000 |
| C | -3.583500 | -1.716500 | 0.000000 |

| | | | |
|---|---|---|---|
| C | -4.817300 | -2.463900 | 0.000000 |
| H | -5.395500 | -2.211800 | 0.890600 |
| H | -5.394400 | -2.213600 | -0.891800 |
| H | -4.595100 | -3.532300 | 0.001200 |
| N | -2.010700 | 1.739000 | 0.000100 |
| C | -2.634900 | 2.701600 | -0.000000 |
| C | -3.421100 | 3.911400 | -0.000300 |
| H | -4.050000 | 3.936200 | -0.891800 |
| H | -4.052400 | 3.935100 | 0.889500 |
| H | -2.757500 | 4.777600 | 0.001100 |

**4. [(NCHS2)Ni$^{I}$(MeCN)]$^{+}$**

| | | | |
|---|---|---|---|
| Ni | -1.204000 | 0.000000 | -0.406500 |
| S | -0.774600 | -2.395600 | 0.038100 |
| S | -0.774500 | 2.395600 | 0.038000 |
| N | 0.645400 | -0.000000 | -1.249000 |
| C | 0.253800 | 0.000000 | 2.013400 |
| H | -0.831800 | 0.000000 | 2.009000 |
| C | 0.931800 | 1.214600 | 1.951600 |
| C | 2.326900 | 1.208800 | 2.001700 |
| H | 2.872400 | 2.144700 | 1.961500 |
| C | 3.017100 | -0.000000 | 2.068200 |
| H | 4.099600 | -0.000000 | 2.113800 |
| C | 2.326800 | -1.208800 | 2.001800 |
| H | 2.872400 | -2.144700 | 1.961500 |
| C | 0.931800 | -1.214600 | 1.951600 |
| C | 0.150800 | -2.463700 | 1.667200 |
| H | -0.651500 | -2.624500 | 2.389100 |
| H | 0.780100 | -3.351800 | 1.645300 |
| C | 0.555800 | -2.460600 | -1.228100 |
| H | 1.264200 | -3.246500 | -0.969600 |
| H | 0.033400 | -2.779700 | -2.134000 |
| C | 1.277200 | -1.162800 | -1.500000 |
| C | 2.558200 | -1.195300 | -2.038900 |
| H | 3.038400 | -2.147200 | -2.224100 |
| C | 3.204200 | -0.000100 | -2.319200 |
| H | 4.203600 | -0.000100 | -2.736000 |
| C | 2.558300 | 1.195200 | -2.038900 |
| H | 3.038500 | 2.147000 | -2.224100 |
| C | 1.277300 | 1.162700 | -1.500000 |
| C | 0.555900 | 2.460600 | -1.228200 |
| H | 0.033600 | 2.779700 | -2.134100 |
| H | 1.264300 | 3.246500 | -0.969700 |

| | | | |
|---|---|---|---|
| C | 0.150900 | 2.463700 | 1.667200 |
| H | 0.780200 | 3.351900 | 1.645300 |
| H | -0.651500 | 2.624500 | 2.389100 |
| N | -3.149000 | -0.000000 | -0.274900 |
| C | -4.298700 | -0.000000 | -0.225300 |
| C | -5.742800 | 0.000100 | -0.156900 |
| H | -6.136100 | 0.888700 | -0.653500 |
| H | -6.136100 | -0.890700 | -0.649900 |
| H | -6.061500 | 0.002200 | 0.887000 |

**5. $[(NCS2)Ni^{I}(MeCN)]^{+}$**

| | | | |
|---|---|---|---|
| Ni | -1.044300 | 0.000200 | 0.536100 |
| S | -0.721900 | -2.474500 | 0.279900 |
| S | -0.721300 | 2.474600 | 0.280700 |
| N | -0.011300 | 0.000200 | -1.544600 |
| C | 0.764400 | -0.000200 | 1.367900 |
| C | 1.497000 | 1.196500 | 1.510400 |
| C | 2.860800 | 1.201900 | 1.822000 |
| H | 3.398000 | 2.141900 | 1.916900 |
| C | 3.544100 | -0.000700 | 1.992100 |
| H | 4.602800 | -0.000900 | 2.227400 |
| C | 2.860400 | -1.203100 | 1.821800 |
| H | 3.397300 | -2.143300 | 1.916600 |
| C | 1.496600 | -1.197200 | 1.510200 |
| C | 0.804100 | -2.534100 | 1.321000 |
| H | 0.425700 | -2.921300 | 2.272200 |
| H | 1.472900 | -3.293100 | 0.911900 |
| C | -0.095500 | -2.439400 | -1.456900 |
| H | 0.573400 | -3.285500 | -1.608100 |
| H | -0.996100 | -2.620800 | -2.050000 |
| C | 0.561400 | -1.156800 | -1.905000 |
| C | 1.719600 | -1.196400 | -2.678400 |
| H | 2.159300 | -2.147900 | -2.948800 |
| C | 2.302500 | 0.000200 | -3.076000 |
| H | 3.206700 | 0.000200 | -3.672400 |
| C | 1.719700 | 1.196800 | -2.678100 |
| H | 2.159600 | 2.148400 | -2.948300 |
| C | 0.561600 | 1.157200 | -1.904700 |
| C | -0.095200 | 2.439800 | -1.456300 |
| H | -0.995900 | 2.621200 | -2.049200 |
| H | 0.573700 | 3.285800 | -1.607600 |
| C | 0.804900 | 2.533600 | 1.321500 |
| H | 1.473900 | 3.292500 | 0.912400 |

| | | | |
|---|---|---|---|
| H | 0.426800 | 2.920900 | 2.272700 |
| N | -2.996200 | 0.000200 | 0.371700 |
| C | -4.147100 | 0.000100 | 0.296300 |
| C | -5.592500 | -0.000000 | 0.211300 |
| H | -5.933200 | 0.886900 | -0.326000 |
| H | -5.933300 | -0.890100 | -0.320800 |
| H | -6.026400 | 0.002900 | 1.213200 |

**5. [(NCS2)Ni$^{III}$(H)(MeCN)]$^+$**

| | | | |
|---|---|---|---|
| Ni | -1.203900 | -0.000100 | 0.409200 |
| N | -3.148800 | -0.000100 | 0.277400 |
| C | -4.298400 | -0.000000 | 0.225200 |
| C | -5.742400 | 0.000100 | 0.153800 |
| H | -6.084900 | 0.890500 | -0.376500 |
| H | -6.084900 | -0.889300 | -0.378100 |
| H | -6.161800 | -0.000800 | 1.161400 |
| S | -0.774400 | -2.395700 | -0.036500 |
| S | -0.774600 | 2.395600 | -0.037300 |
| C | 1.278300 | 1.163000 | 1.499400 |
| C | 2.559700 | 1.195700 | 2.037500 |
| H | 3.040000 | 2.147600 | 2.222200 |
| C | 3.205900 | 0.000600 | 2.317500 |
| H | 4.205600 | 0.000700 | 2.733800 |
| C | 2.559900 | -1.194700 | 2.037800 |
| H | 3.040400 | -2.146600 | 2.222700 |
| C | 1.278600 | -1.162500 | 1.499600 |
| C | 0.557500 | -2.460600 | 1.228100 |
| H | 0.036600 | -2.780500 | 2.134600 |
| H | 1.265900 | -3.245900 | 0.968200 |
| C | 0.149400 | -2.463900 | -1.666400 |
| H | 0.778800 | -3.352000 | -1.644900 |
| H | -0.653500 | -2.625000 | -2.387600 |
| C | 0.930000 | -1.214800 | -1.951900 |
| C | 2.325000 | -1.209200 | -2.004500 |
| H | 2.870600 | -2.145000 | -1.965200 |
| C | 3.015200 | -0.000400 | -2.072300 |
| H | 4.097700 | -0.000500 | -2.119700 |
| C | 2.325200 | 1.208500 | -2.004800 |
| H | 2.870900 | 2.144300 | -1.965600 |
| C | 0.930200 | 1.214400 | -1.952200 |
| C | 0.149700 | 2.463600 | -1.666900 |
| H | -0.652900 | 2.624800 | -2.388400 |
| H | 0.779300 | 3.351600 | -1.645200 |

| | | | |
|---|---|---|---|
| C | 0.556800 | 2.460800 | 1.227800 |
| H | 1.265100 | 3.246500 | 0.968100 |
| H | 0.035500 | 2.780500 | 2.134000 |
| H | -0.833500 | -0.000100 | -2.005700 |
| C | 0.252100 | -0.000200 | -2.012600 |
| N | 0.646400 | 0.000200 | 1.249100 |

**6. [(NCS2)Ni$^{II}$(H)(MeCN)], singlet**

| | | | |
|---|---|---|---|
| Ni | -1.528200 | -0.052400 | -0.739100 |
| S | 0.024300 | -2.835600 | 0.536300 |
| S | -1.186100 | 2.104600 | 0.030300 |
| C | 0.938900 | 1.298600 | 1.763100 |
| C | 2.229500 | 1.820700 | 1.825000 |
| H | 2.387800 | 2.891600 | 1.830600 |
| C | 3.299900 | 0.934300 | 1.861500 |
| H | 4.317300 | 1.303200 | 1.913000 |
| C | 3.048100 | -0.428100 | 1.792300 |
| H | 3.860300 | -1.143800 | 1.770000 |
| C | 1.721900 | -0.868200 | 1.733300 |
| C | 1.426800 | -2.348700 | 1.626100 |
| H | 1.126000 | -2.737100 | 2.603300 |
| H | 2.327800 | -2.886200 | 1.330900 |
| C | 0.634500 | -2.563400 | -1.197500 |
| H | 1.426400 | -3.286500 | -1.393200 |
| H | -0.242800 | -2.854200 | -1.779900 |
| C | 1.088600 | -1.171900 | -1.564000 |
| C | 2.395700 | -1.033700 | -2.047500 |
| H | 3.015600 | -1.917000 | -2.164600 |
| C | 2.919300 | 0.216600 | -2.361300 |
| H | 3.930100 | 0.307700 | -2.742400 |
| C | 2.149700 | 1.350100 | -2.126900 |
| H | 2.569200 | 2.336300 | -2.299000 |
| C | 0.847900 | 1.221700 | -1.634700 |
| C | 0.075500 | 2.466900 | -1.274800 |
| H | -0.526200 | 2.843100 | -2.106300 |
| H | 0.718000 | 3.281500 | -0.939500 |
| C | -0.252700 | 2.203000 | 1.644100 |
| H | 0.022900 | 3.246800 | 1.783200 |
| H | -1.016900 | 1.940700 | 2.377000 |
| N | -3.324800 | -0.191000 | -0.094100 |
| C | -4.396200 | -0.298700 | 0.308900 |
| C | -5.743900 | -0.438300 | 0.813800 |
| H | -6.407300 | 0.249500 | 0.286300 |

| | | | |
|---|---|---|---|
| H | -6.092000 | -1.461200 | 0.660400 |
| H | -5.764700 | -0.207400 | 1.880200 |
| C | 0.258300 | -0.040100 | -1.406600 |
| N | 0.697700 | -0.016500 | 1.766100 |
| H | -1.731800 | -1.217500 | -1.613600 |

**7. [(NCS2)Ni$^{II}$(H)(MeCN)], triplet**

| | | | |
|---|---|---|---|
| Ni | -0.723300 | -0.002800 | -1.040900 |
| S | -0.485900 | -2.470600 | -0.696800 |
| S | -0.491500 | 2.467200 | -0.703700 |
| C | -0.162800 | 1.160900 | 1.807300 |
| C | 0.500400 | 1.201400 | 3.031200 |
| H | 0.751500 | 2.154200 | 3.478900 |
| C | 0.838800 | 0.006300 | 3.651400 |
| H | 1.363200 | 0.008600 | 4.599100 |
| C | 0.505000 | -1.191800 | 3.034500 |
| H | 0.759800 | -2.142500 | 3.484800 |
| C | -0.158300 | -1.157200 | 1.810500 |
| C | -0.571900 | -2.443700 | 1.142300 |
| H | -1.626900 | -2.639400 | 1.351000 |
| H | 0.000200 | -3.282100 | 1.536300 |
| C | 1.326300 | -2.532700 | -1.052800 |
| H | 1.770600 | -3.287400 | -0.402100 |
| H | 1.362100 | -2.929100 | -2.071600 |
| C | 2.042300 | -1.198000 | -0.965300 |
| C | 3.435000 | -1.202100 | -0.826000 |
| H | 3.977600 | -2.140600 | -0.756700 |
| C | 4.131300 | 0.002900 | -0.763100 |
| H | 5.209300 | 0.004100 | -0.646600 |
| C | 3.432500 | 1.206400 | -0.827500 |
| H | 3.973200 | 2.146100 | -0.759200 |
| C | 2.039800 | 1.199400 | -0.966700 |
| C | 1.321300 | 2.532700 | -1.055500 |
| H | 1.359000 | 2.929600 | -2.074100 |
| H | 1.762700 | 3.288000 | -0.403600 |
| C | -0.581400 | 2.443700 | 1.135400 |
| H | -0.014200 | 3.285800 | 1.528700 |
| H | -1.637800 | 2.634800 | 1.341400 |
| N | -2.905900 | -0.001700 | -0.689000 |
| C | -4.053100 | -0.002400 | -0.610800 |
| C | -5.498100 | -0.003600 | -0.523000 |
| H | -5.895800 | 0.899100 | -0.989600 |
| H | -5.897000 | -0.878200 | -1.039400 |

| | | | |
|---|---|---|---|
| H | -5.806400 | -0.032800 | 0.523100 |
| C | 1.316500 | -0.000100 | -1.044800 |
| N | -0.480500 | 0.000400 | 1.214900 |
| H | -0.995400 | -0.007800 | -2.690800 |

**8. $[(NCS2)Ni^{III}(MeCN)_2]^{2+}$**

| | | | |
|---|---|---|---|
| Ni | -0.559900 | -0.414700 | -0.000200 |
| S | -0.402800 | -0.306500 | -2.381300 |
| S | -0.368600 | -0.353400 | 2.379200 |
| C | 2.157000 | -0.723200 | 1.144200 |
| C | 3.545200 | -0.715500 | 1.171500 |
| H | 4.062000 | -0.745000 | 2.121300 |
| C | 4.241700 | -0.666300 | -0.027500 |
| H | 5.324100 | -0.643900 | -0.030700 |
| C | 3.537100 | -0.651400 | -1.222000 |
| H | 4.047300 | -0.630200 | -2.175600 |
| C | 2.148400 | -0.661700 | -1.185800 |
| C | 1.374600 | -0.747300 | -2.476200 |
| H | 1.382500 | -1.783200 | -2.822900 |
| H | 1.836100 | -0.138300 | -3.251900 |
| C | -0.383200 | 1.527700 | -2.511100 |
| H | 0.320900 | 1.807000 | -3.294300 |
| H | -1.385900 | 1.783200 | -2.861400 |
| C | -0.094000 | 2.192200 | -1.199300 |
| C | 0.230200 | 3.552500 | -1.175200 |
| H | 0.325700 | 4.087000 | -2.113400 |
| C | 0.424400 | 4.212300 | 0.030900 |
| H | 0.682300 | 5.264100 | 0.036400 |
| C | 0.285000 | 3.526800 | 1.229700 |
| H | 0.422500 | 4.041400 | 2.173900 |
| C | -0.036700 | 2.165100 | 1.240200 |
| C | -0.256100 | 1.475000 | 2.553100 |
| H | -1.213800 | 1.768400 | 2.988400 |
| H | 0.520700 | 1.697400 | 3.284300 |
| C | 1.387400 | -0.878400 | 2.429900 |
| H | 1.878200 | -0.352900 | 3.247400 |
| H | 1.347900 | -1.937900 | 2.693700 |
| N | -0.884200 | -2.398100 | -0.015400 |
| C | -1.089100 | -3.526400 | -0.026200 |
| C | -1.349900 | -4.945100 | -0.039600 |
| H | -1.924400 | -5.216900 | 0.847600 |
| H | -1.919700 | -5.201200 | -0.934500 |
| H | -0.404400 | -5.489700 | -0.041900 |

| | | | |
|---|---|---|---|
| C | -0.202100 | 1.516400 | 0.016700 |
| N | 1.487100 | -0.676700 | -0.018500 |
| N | -2.561800 | -0.034200 | 0.014700 |
| C | -3.686200 | 0.194000 | 0.027800 |
| C | -5.100200 | 0.480800 | 0.045000 |
| H | -5.537100 | 0.218400 | -0.919900 |
| H | -5.580500 | -0.102700 | 0.832200 |
| H | -5.252900 | 1.544400 | 0.236200 |

**9. [(NCHS2)Ni$^{II}$(H)(MeCN)]$^{+}$, singlet**

| | | | |
|---|---|---|---|
| Ni | -0.952800 | 0.000600 | -0.888400 |
| S | -0.385800 | -2.235900 | -0.983900 |
| S | -0.383700 | 2.236600 | -0.984100 |
| C | -0.034100 | 1.167600 | 1.599800 |
| C | 0.448500 | 1.198700 | 2.902000 |
| H | 0.643600 | 2.148700 | 3.381300 |
| C | 0.684900 | 0.000000 | 3.560800 |
| H | 1.065700 | -0.000100 | 4.574400 |
| C | 0.447400 | -1.198500 | 2.902100 |
| H | 0.641700 | -2.148600 | 3.381500 |
| C | -0.035100 | -1.167000 | 1.599900 |
| C | -0.333300 | -2.436700 | 0.850700 |
| H | -1.333400 | -2.791600 | 1.111400 |
| H | 0.371900 | -3.226500 | 1.105700 |
| C | 1.429600 | -2.418600 | -1.482800 |
| H | 1.783300 | -3.338300 | -1.020200 |
| H | 1.363300 | -2.564200 | -2.561200 |
| C | 2.230900 | -1.213600 | -1.109800 |
| C | 3.171700 | -1.210900 | -0.078100 |
| H | 3.484100 | -2.145700 | 0.373200 |
| C | 3.679600 | -0.001600 | 0.395000 |
| H | 4.419700 | -0.001900 | 1.186200 |
| C | 3.172900 | 1.208300 | -0.078100 |
| H | 3.486100 | 2.142700 | 0.373100 |
| C | 2.232000 | 1.211700 | -1.109800 |
| C | 1.431900 | 2.417500 | -1.482900 |
| H | 1.365800 | 2.563100 | -2.561300 |
| H | 1.786400 | 3.336900 | -1.020400 |
| C | -0.331100 | 2.437500 | 0.850400 |
| H | 0.374700 | 3.226700 | 1.105500 |
| H | -1.330900 | 2.793300 | 1.111100 |
| N | -3.023900 | 0.001200 | -0.366200 |
| C | -4.152600 | 0.000700 | -0.144500 |

| | | | |
|---|---|---|---|
| C | -5.573400 | -0.000000 | 0.131000 |
| H | -6.033200 | 0.889300 | -0.302900 |
| H | -6.032500 | -0.889200 | -0.303900 |
| H | -5.740500 | -0.000600 | 1.209200 |
| H | -1.288500 | 0.000700 | -2.311400 |
| N | -0.283300 | 0.000400 | 0.976600 |
| C | 1.857600 | -0.000700 | -1.679800 |
| H | 1.149200 | -0.000400 | -2.498100 |

**10. [(NCHS2)Ni$^{II}$(H)(MeCN)]$^{+}$, triplet**

| | | | |
|---|---|---|---|
| Ni | -0.952600 | 0.008100 | -1.055900 |
| S | -0.409500 | -2.439200 | -0.925400 |
| S | -0.394100 | 2.450200 | -0.906700 |
| C | -0.007100 | 1.156200 | 1.611400 |
| C | 0.442200 | 1.181900 | 2.927300 |
| H | 0.623700 | 2.131200 | 3.413800 |
| C | 0.659300 | -0.016000 | 3.590000 |
| H | 1.013000 | -0.021500 | 4.613400 |
| C | 0.430300 | -1.207100 | 2.918700 |
| H | 0.602100 | -2.161600 | 3.398300 |
| C | -0.018500 | -1.167400 | 1.603000 |
| C | -0.308700 | -2.476700 | 0.905500 |
| H | -1.293100 | -2.836200 | 1.216300 |
| H | 0.416000 | -3.233100 | 1.203800 |
| C | 1.384100 | -2.467800 | -1.464900 |
| H | 1.830700 | -3.362600 | -1.035700 |
| H | 1.310200 | -2.596400 | -2.545400 |
| C | 2.125500 | -1.218500 | -1.088900 |
| C | 3.126700 | -1.217100 | -0.117100 |
| H | 3.475900 | -2.153900 | 0.300700 |
| C | 3.654700 | -0.009700 | 0.335300 |
| H | 4.438400 | -0.013500 | 1.083400 |
| C | 3.133700 | 1.202700 | -0.111900 |
| H | 3.488400 | 2.135700 | 0.310000 |
| C | 2.132500 | 1.214200 | -1.083600 |
| C | 1.397700 | 2.469300 | -1.453400 |
| H | 1.320600 | 2.601600 | -2.533200 |
| H | 1.851400 | 3.360000 | -1.023000 |
| C | -0.283900 | 2.473700 | 0.923800 |
| H | 0.452400 | 3.218500 | 1.223200 |
| H | -1.261500 | 2.844100 | 1.242800 |
| N | -2.907400 | 0.009900 | -0.414800 |
| C | -4.015500 | 0.008400 | -0.114000 |

| | | | |
|---|---|---|---|
| C | -5.411000 | 0.006000 | 0.259500 |
| H | -5.858600 | 0.968300 | 0.005800 |
| H | -5.931800 | -0.787200 | -0.279400 |
| H | -5.504100 | -0.164700 | 1.333200 |
| H | -1.298800 | 0.015900 | -2.615500 |
| N | -0.237500 | -0.002100 | 0.967800 |
| C | 1.706500 | 0.000200 | -1.619800 |
| H | 0.979000 | 0.003900 | -2.427800 |

(c) Energy of other reactions in the electrocatalytic cycle

**1. Relative energies of protonation of C-H activated $Ni^{II}$ complexes**

We evaluated the relative energies of protonation of the first protonation event from (NCS2)$Ni^{II}$(H)(MeCN). There are two possibilities:
(a) The protonation of the Ni-C bond
(b) Proton-hydride recombination to evolve $H_2$
The reaction free energy differences showed that the protonation of the hydride is endergonic while the protonation of the Ni-C bond is exergonic:

| Reaction | Spin State | Energy (Hartree) | Energy (kcal/mol) |
|---|---|---|---|
| | | | |
| (NCS2)$Ni^{II}$H + $H^+$ → (NCS2)$Ni^{II}$ + $H_2$ | S = 0 | 0.035139 | 22.05 |
| (NCS2)$Ni^{II}$H + $H^+$ → (NCS2)$Ni^{II}$ + $H_2$ | S = 1 | 0.027859 | 17.48 |
| (NCS2)$Ni^{II}$H + $H^+$ → (NCHS2)$Ni^{II}$(H) | S = 0 | -0.044679 | -28.04 |
| (NCS2)$Ni^{II}$H + $H^+$ → (NCHS2)$Ni^{II}$(H) | S = 1 | -0.047555 | -29.84 |

These calculations prove that the $Ni^{II}$-C bond is more basic than the $Ni^{II}$-H and is more likely to be protonated first.

**2. Hydrogen evolution from $Ni^{III}$**

**Protonation of [(NCS2)$Ni^{III}$(H)(MeCN)]$^+$**

Energetics of heterolytic $H_2$ evolution was evaluated and shown to be endergonic for the following reaction:

$$\mathbf{4} + \text{TfOH} + \text{MeCN} \rightarrow \text{TfO}^- + [(\text{NCS2})\text{Ni}^{III}(\text{MeCN})_2]^+ + H_2$$

| Reaction | Energy (hartree) | Energy (kcal/mol) |
|---|---|---|
| heterolytic protonation at $Ni^{III}$ | 0.041587 | 26.09 |

As two catalytic peaks were seen at lower acid concentrations, we considered other mechanistic possibilities. Based on our hypothesis, an inverse KIE for the first catalytic peak suggests the cleavage of a weak chemical bond to form a strong bond is involved in the mechanism. Thus, we can propose a pathway which involves the protonolysis of the $Ni^{III}$-H species to form a $Ni^{III}$-solvento complex and evolve $H_2$. The $Ni^{III}$-solvento would then get reduced and protonated at the Ni-C bond to regenerate the resting state of the catalyst. We evaluated the energetics of this pathway involving protonolysis of the $Ni^{III}$-H as shown below (Fig. S92), with the results suggesting that it is an unlikely process.

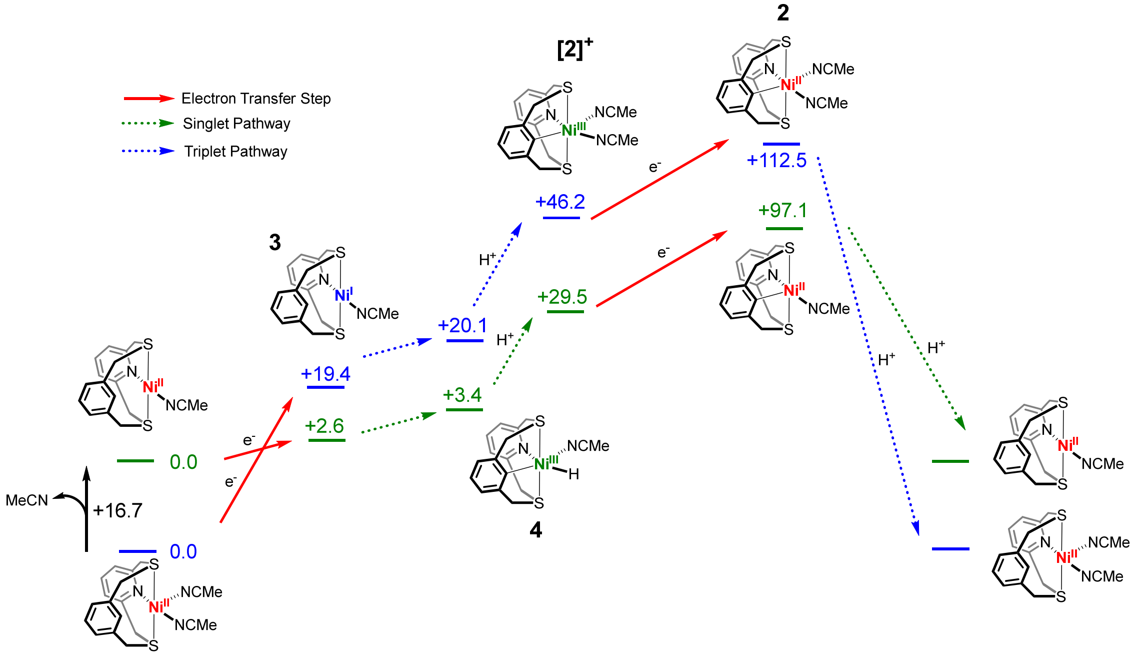


**Figure S92.** Energetics of the pathway involving the protonation of the $Ni^{III}$-H intermediate **4**. The calculated $E_{cat}$ is -2.93 V for the singlet pathway and -2.86 V for the triplet pathway.

### 3. Concerted metalation-deprotonation at $Ni^{I}$

Protonation of a $Ni^{I}$ species is a viable route to forming $Ni^{III}$-H. Since the structural evidence suggests that the $Ni^{III}$ complexes are C-H activated, we looked at the possibility of forming a $Ni^{III}$-H complex by the direct protonation of a C-H activated $Ni^{I}$ computationally.

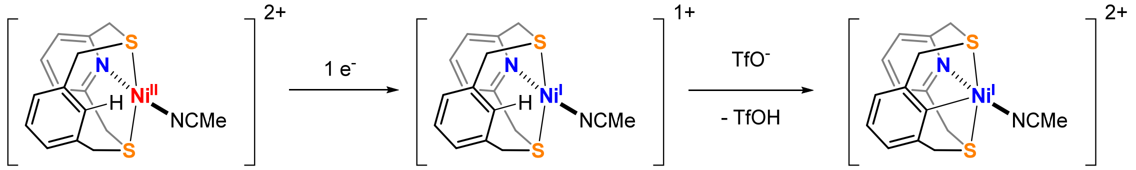

The energy for this overall reaction is +42.5 kcal/mol. Thus, we ruled out the possibility of forming the $Ni^{III}$-H species directly from the protonation of a C-H activated $Ni^{I}$ complex.

**4. Equilibrium between 3 and 4**

The energy difference between **3** and **4** was found to be ~0.8 kcal/mol and a transition state was calculated which showed C-H activation at $Ni^{I}$ to form a $Ni^{III}$-H. The energy profile is shown below:

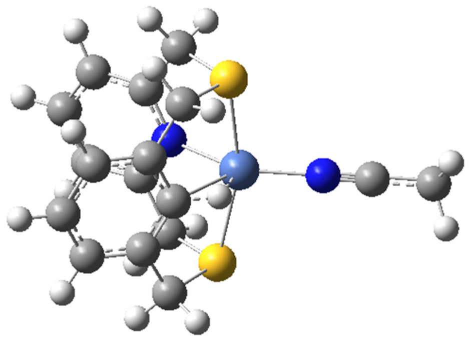

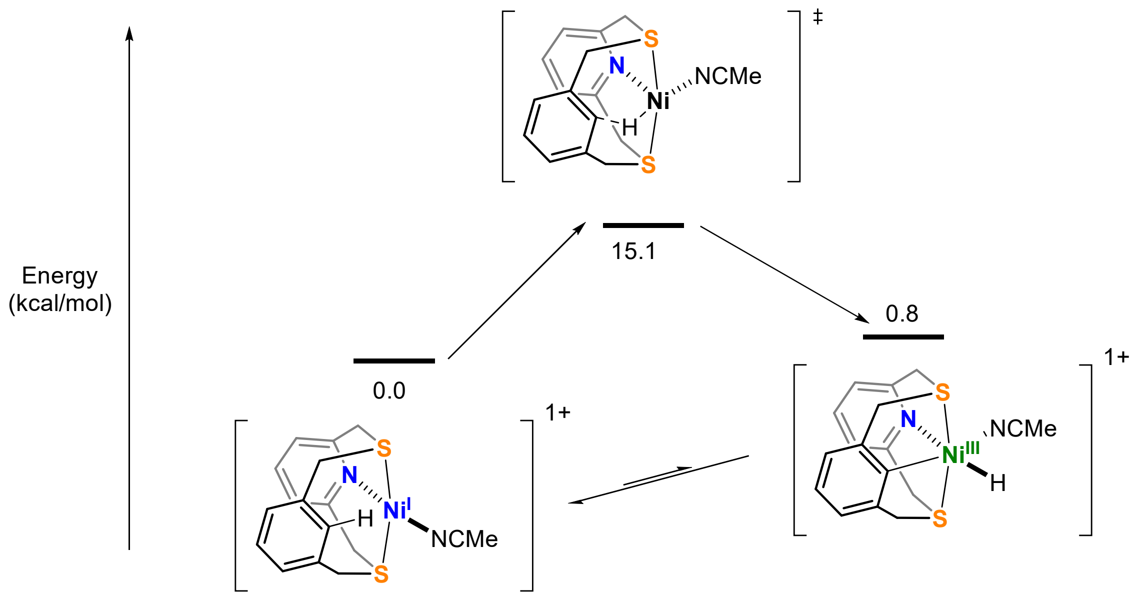


**Figure S93.** Energy diagram for the conversion of **3** to **4**.

## 12. Supplementary References


1. Moriguchi, T., Kitamura, S., Sakata, K. & Tsuge, A. Syntheses and Structures of Dichloropalladium(II)(Dithia[3.3]Metadipyridinophane) and Dichloroplatinum(II)(Dithia[3.3]Metadipyridinophane) Complexes. *Polyhedron* **20**, 2315-2320 (2001).
2. Steeneck, C., Kinzel, O., Gege, C., Kleymann, G. & Hoffmann, T. Pyrrolo sulfonamide compounds for modulation of orphan nuclear receptor Rar-related Orphan Receptor-gamma (RORgamma, NR1F3) activity and for the treatment of chronic inflammatory and autoimmune diseases. WO 2012/139775Al (2012).
3. Gemel, C., Folting, K. & Caulton, K. G. New Approach to Ru(II) Pincer Ligand Chemistry. Bis(tert-butylaminomethyl)pyridine Coordinated to Ruthenium(II). *Inorg. Chem.* **39**, 1593-1597 (2000).
4. Constable, E. C., King, A. C. & Raithby, P. R. Synthesis, coordination chemistry and crystal structures of [2+2] macrocycles incorporating 2,2-bis(thiomethyl)pyridine sub-units. *Polyhedron* **17**, 4275-4289 (1998).
5. Luo, J., Khusnutdinova, J. R., Rath, N. P. & Mirica, L. M. Unsupported $d^8$-$d^8$ Interactions in Cationic $Pd^{II}$ and $Pt^{II}$ Complexes: Evidence for a Significant Metal-Metal Bonding Character. *Chem. Commun.* **48**, 1532-1534 (2012).
6. Martin, D. J., Mercado, B. Q. & Mayer, J. M. Combining scaling relationships overcomes rate versus overpotential trade-offs in O-2 molecular electrocatalysis. *Sci. Adv.* **6**, eaaz3318 (2020).
7. Appel, A. M. & Helm, M. L. Determining the Overpotential for a Molecular Electrocatalyst. *ACS Catalysis* **4**, 630-633 (2014).
8. Savéant, J.-M. & Costentin, C. *Elements of Molecular and Biomolecular Electrochemistry: An Electrochemical Approach to Electron Transfer Chemistry*. 2nd edn, (2019).
9. Lee, K. J., Elgrishi, N., Kandemir, B. & Dempsey, J. L. Electrochemical and spectroscopic methods for evaluating molecular electrocatalysts. *Nature Rev. Chem.* **1** (2017).
10. Sinha, S., Tran, G. N., Na, H. & Mirica, L. M. Electrocatalytic H2 Evolution Promoted by a Bioinspired (N2S2)Ni(II) Complex. *Chem. Comm.* **58**, 1143-1146 (2022).
11. Gu, N. X., Oyala, P. H. & Peters, J. C. H2 Evolution from a Thiolate-Bound Ni(III) Hydride. *J. Am. Chem. Soc.* **142**, 7827-7835 (2020).
12. Lai, K.-T., Ho, W.-C., Chiou, T.-W. & Liaw, W.-F. Formation of [NiIII(κ1-S2CH)(P(o-C6H3-3-SiMe3-2-S)3)]− via CS2 Insertion into Nickel(III) Hydride Containing [NiIII(H)(P(o-C6H3-3-SiMe3-2-S)3)]−. *Inorg. Chem.* **52**, 4151-4153 (2013).
13. Sheldrick, G. M. Bruker-SHELXTL. *Acta Cryst.* **A64**, 112-122 (2008).
14. Frisch, M. J. *et al.* Gaussian 16 Rev. B.01. *Gaussian 16 Rev. B.01*, Gaussian, Inc., Wallingford CT (2016).
15. Becke, A. D. Density-functional thermochemistry. III. The role of exact exchange. *J. Chem. Phys.* **98**, 5648-5652 (1993).
16. Lee, C. T., Yang, W. T. & Parr, R. G. Development of the Colle-Salvetti Correlation-Energy Formula into a Functional of the Electron-Density. *Phys. Rev. B: Cond. Mat. Mat. Phys.* **37**, 785-789 (1988).
17. Schafer, A., Horn, H. & Ahlrichs, R. Fully Optimized Contracted Gaussian-Basis Sets for Atoms Li to Kr. *J. Chem. Phys.* **97**, 2571-2577 (1992).

18. Schafer, A., Huber, C. & Ahlrichs, R. Fully Optimized Contracted Gaussian Basis Sets of Triple Zeta Valence Quality for Atoms Li to Kr. *J. Chem. Phys.* **100**, 5829-5835 (1994).
19. Na, H. & Mirica, L. M. Deciphering the mechanism of the Ni-photocatalyzed C–O cross-coupling reaction using a tridentate pyridinophane ligand. *Nature Commun.* **13**, 1313 (2022).
20. Skripnikov, L. (www.chemissian.com, accessed June 2019).
