# Supplementary material for: Characterization of paramagnetic states in an organometallic nickel hydrogen evolution electrocatalyst
Source: Nat Commun. 2023 Feb 17;14:905. doi: 10.1038/s41467-023-36609-7 (PMC9938211; doi:10.1038/s41467-023-36609-7)
Supplement: Supplementary file 3 — Description of Additional Supplementary Files [file 41467_2023_36609_MOESM3_ESM.pdf]

Legend for Supplementary Data 1
Description: Bond lengths [Å] and angles [°] for NCHS2

Legend for Supplementary Data 2
Description: Bond lengths [Å] and angles [°] for **1**
