# Supplementary material for: Characterization of paramagnetic states in an organometallic nickel hydrogen evolution electrocatalyst
Source: Nat Commun. 2023 Feb 17;14:905. doi: 10.1038/s41467-023-36609-7 (PMC9938211; doi:10.1038/s41467-023-36609-7)
Supplement: Supplementary file 4 — Supplementary Data 1 [file 41467_2023_36609_MOESM4_ESM.pdf]

Supplementary Data 1

Bond lengths [Å] and angles [°] for NCHS2

S(1)-C(2)	1.820(3)
S(1)-C(1)	1.825(3)
S(2)-C(10)	1.815(3)
S(2)-C(9)	1.825(2)
C(1)-C(13)	1.499(3)
C(1)-H(1A)	0.9900
C(1)-H(1B)	0.9900
C(2)-C(3)	1.507(3)
C(2)-H(2A)	0.9900
C(2)-H(2B)	0.9900
C(3)-C(8)	1.383(3)
C(3)-C(4)	1.393(3)
C(4)-C(5)	1.388(4)
C(4)-H(4)	0.9500
C(5)-C(6)	1.397(4)
C(5)-H(5)	0.9500
C(6)-C(7)	1.393(3)
C(6)-H(6)	0.9500
C(7)-C(8)	1.391(3)
C(7)-C(9)	1.510(3)
C(8)-H(8)	0.9500
C(9)-H(9A)	0.9900
C(9)-H(9B)	0.9900
C(10)-C(11)	1.507(4)
C(10)-H(10A)	0.9900
C(10)-H(10B)	0.9900
C(11)-N(12)	1.351(3)
C(11)-C(16)	1.391(3)
N(12)-C(13)	1.344(3)
C(13)-C(14)	1.397(3)
C(14)-C(15)	1.384(4)
C(14)-H(14)	0.9500

C(15)-C(16)	1.382(4)
C(15)-H(15)	0.9500
C(16)-H(16)	0.9500
S(1A)-C(1A)	1.816(2)
S(1A)-C(2A)	1.825(3)
S(2A)-C(9A)	1.815(2)
S(2A)-C(10A)	1.823(3)
C(1A)-C(13A)	1.502(3)
C(1A)-H(1AA)	0.9900
C(1A)-H(1AB)	0.9900
C(2A)-C(3A)	1.498(3)
C(2A)-H(2AA)	0.9900
C(2A)-H(2AB)	0.9900
C(3A)-C(8A)	1.386(3)
C(3A)-C(4A)	1.390(3)
C(4A)-C(5A)	1.382(4)
C(4A)-H(4A)	0.9500
C(5A)-C(6A)	1.398(4)
C(5A)-H(5A)	0.9500
C(6A)-C(7A)	1.387(3)
C(6A)-H(6A)	0.9500
C(7A)-C(8A)	1.388(3)
C(7A)-C(9A)	1.512(3)
C(8A)-H(8A)	0.9500
C(9A)-H(9AA)	0.9900
C(9A)-H(9AB)	0.9900
C(10A)-C(11A)	1.504(4)
C(10A)-H(10C)	0.9900
C(10A)-H(10D)	0.9900
C(11A)-N(12A)	1.346(3)
C(11A)-C(16A)	1.388(3)
N(12A)-C(13A)	1.353(3)
C(13A)-C(14A)	1.394(3)
C(14A)-C(15A)	1.381(4)
C(14A)-H(14A)	0.9500
C(15A)-C(16A)	1.391(4)

C(15A)-H(15A)	0.9500
C(16A)-H(16A)	0.9500
C(2)-S(1)-C(1)	102.02(12)
C(10)-S(2)-C(9)	101.27(12)
C(13)-C(1)-S(1)	115.82(17)
C(13)-C(1)-H(1A)	108.3
S(1)-C(1)-H(1A)	108.3
C(13)-C(1)-H(1B)	108.3
S(1)-C(1)-H(1B)	108.3
H(1A)-C(1)-H(1B)	107.4
C(3)-C(2)-S(1)	113.24(17)
C(3)-C(2)-H(2A)	108.9
S(1)-C(2)-H(2A)	108.9
C(3)-C(2)-H(2B)	108.9
S(1)-C(2)-H(2B)	108.9
H(2A)-C(2)-H(2B)	107.7
C(8)-C(3)-C(4)	119.3(2)
C(8)-C(3)-C(2)	119.8(2)
C(4)-C(3)-C(2)	120.8(2)
C(5)-C(4)-C(3)	120.0(2)
C(5)-C(4)-H(4)	120.0
C(3)-C(4)-H(4)	120.0
C(4)-C(5)-C(6)	120.2(2)
C(4)-C(5)-H(5)	119.9
C(6)-C(5)-H(5)	119.9
C(7)-C(6)-C(5)	120.1(2)
C(7)-C(6)-H(6)	120.0
C(5)-C(6)-H(6)	120.0
C(8)-C(7)-C(6)	118.8(2)
C(8)-C(7)-C(9)	119.5(2)
C(6)-C(7)-C(9)	121.7(2)
C(3)-C(8)-C(7)	121.6(2)
C(3)-C(8)-H(8)	119.2
C(7)-C(8)-H(8)	119.2
C(7)-C(9)-S(2)	115.03(17)

C(7)-C(9)-H(9A)	108.5
S(2)-C(9)-H(9A)	108.5
C(7)-C(9)-H(9B)	108.5
S(2)-C(9)-H(9B)	108.5
H(9A)-C(9)-H(9B)	107.5
C(11)-C(10)-S(2)	113.71(17)
C(11)-C(10)-H(10A)	108.8
S(2)-C(10)-H(10A)	108.8
C(11)-C(10)-H(10B)	108.8
S(2)-C(10)-H(10B)	108.8
H(10A)-C(10)-H(10B)	107.7
N(12)-C(11)-C(16)	121.7(2)
N(12)-C(11)-C(10)	116.5(2)
C(16)-C(11)-C(10)	121.8(2)
C(13)-N(12)-C(11)	118.8(2)
N(12)-C(13)-C(14)	122.2(2)
N(12)-C(13)-C(1)	115.7(2)
C(14)-C(13)-C(1)	122.1(2)
C(15)-C(14)-C(13)	118.6(2)
C(15)-C(14)-H(14)	120.7
C(13)-C(14)-H(14)	120.7
C(16)-C(15)-C(14)	119.2(2)
C(16)-C(15)-H(15)	120.4
C(14)-C(15)-H(15)	120.4
C(15)-C(16)-C(11)	119.3(2)
C(15)-C(16)-H(16)	120.3
C(11)-C(16)-H(16)	120.3
C(1A)-S(1A)-C(2A)	101.56(12)
C(9A)-S(2A)-C(10A)	100.61(11)
C(13A)-C(1A)-S(1A)	116.01(17)
C(13A)-C(1A)-H(1AA)	108.3
S(1A)-C(1A)-H(1AA)	108.3
C(13A)-C(1A)-H(1AB)	108.3
S(1A)-C(1A)-H(1AB)	108.3
H(1AA)-C(1A)-H(1AB)	107.4
C(3A)-C(2A)-S(1A)	114.02(17)

C(3A)-C(2A)-H(2AA)	108.7
S(1A)-C(2A)-H(2AA)	108.7
C(3A)-C(2A)-H(2AB)	108.7
S(1A)-C(2A)-H(2AB)	108.7
H(2AA)-C(2A)-H(2AB)	107.6
C(8A)-C(3A)-C(4A)	118.7(2)
C(8A)-C(3A)-C(2A)	120.2(2)
C(4A)-C(3A)-C(2A)	120.9(2)
C(5A)-C(4A)-C(3A)	120.6(2)
C(5A)-C(4A)-H(4A)	119.7
C(3A)-C(4A)-H(4A)	119.7
C(4A)-C(5A)-C(6A)	120.1(2)
C(4A)-C(5A)-H(5A)	120.0
C(6A)-C(5A)-H(5A)	120.0
C(7A)-C(6A)-C(5A)	119.7(2)
C(7A)-C(6A)-H(6A)	120.1
C(5A)-C(6A)-H(6A)	120.1
C(6A)-C(7A)-C(8A)	119.3(2)
C(6A)-C(7A)-C(9A)	121.6(2)
C(8A)-C(7A)-C(9A)	119.1(2)
C(3A)-C(8A)-C(7A)	121.5(2)
C(3A)-C(8A)-H(8A)	119.3
C(7A)-C(8A)-H(8A)	119.3
C(7A)-C(9A)-S(2A)	116.53(17)
C(7A)-C(9A)-H(9AA)	108.2
S(2A)-C(9A)-H(9AA)	108.2
C(7A)-C(9A)-H(9AB)	108.2
S(2A)-C(9A)-H(9AB)	108.2
H(9AA)-C(9A)-H(9AB)	107.3
C(11A)-C(10A)-S(2A)	112.60(17)
C(11A)-C(10A)-H(10C)	109.1
S(2A)-C(10A)-H(10C)	109.1
C(11A)-C(10A)-H(10D)	109.1
S(2A)-C(10A)-H(10D)	109.1
H(10C)-C(10A)-H(10D)	107.8
N(12A)-C(11A)-C(16A)	122.4(2)

N(12A)-C(11A)-C(10A)	116.4(2)
C(16A)-C(11A)-C(10A)	121.2(2)
C(11A)-N(12A)-C(13A)	118.7(2)
N(12A)-C(13A)-C(14A)	121.7(2)
N(12A)-C(13A)-C(1A)	116.2(2)
C(14A)-C(13A)-C(1A)	122.1(2)
C(15A)-C(14A)-C(13A)	119.4(2)
C(15A)-C(14A)-H(14A)	120.3
C(13A)-C(14A)-H(14A)	120.3
C(14A)-C(15A)-C(16A)	118.9(2)
C(14A)-C(15A)-H(15A)	120.5
C(16A)-C(15A)-H(15A)	120.5
C(11A)-C(16A)-C(15A)	118.9(2)
C(11A)-C(16A)-H(16A)	120.5
C(15A)-C(16A)-H(16A)	120.5
