# Supplementary material for: Characterization of paramagnetic states in an organometallic nickel hydrogen evolution electrocatalyst
Source: Nat Commun. 2023 Feb 17;14:905. doi: 10.1038/s41467-023-36609-7 (PMC9938211; doi:10.1038/s41467-023-36609-7)
Supplement: Supplementary file 5 — Supplementary Data 2 [file 41467_2023_36609_MOESM5_ESM.pdf]

Supplementary Data 2

Bond lengths [Å] and angles [°] for **1**

Ni(1)-N(1)	2.006(6)
Ni(1)-O(4)	2.039(5)
Ni(1)-O(1)	2.043(4)
Ni(1)-S(1)	2.391(2)
Ni(1)-S(2)	2.407(2)
Ni(1)-C(1)	2.478(8)
S(1)-C(8)	1.805(7)
S(1)-C(7)	1.844(7)
S(2)-C(14)	1.797(7)
S(2)-C(15)	1.841(8)
S(3)-O(2)	1.425(5)
S(3)-O(3)	1.430(5)
S(3)-O(1)	1.475(4)
S(3)-C(16)	1.796(9)
S(4)-O(6)	1.422(5)
S(4)-O(5)	1.440(5)
S(4)-O(4)	1.478(5)
S(4)-C(17)	1.828(8)
F(1)-C(16)	1.338(9)
F(2)-C(16)	1.346(9)
F(3)-C(16)	1.337(7)
F(4)-C(17)	1.335(9)
F(5)-C(17)	1.318(8)
F(6)-C(17)	1.331(9)
N(1)-C(13)	1.331(9)
N(1)-C(9)	1.365(8)
C(1)-C(2)	1.388(10)
C(1)-C(6)	1.396(9)
C(1)-H(1)	0.94(5)
C(2)-C(3)	1.382(9)
C(2)-C(15)	1.505(9)
C(3)-C(4)	1.377(9)

C(3)-H(3)	0.9500
C(4)-C(5)	1.384(10)
C(4)-H(4)	0.9500
C(5)-C(6)	1.392(10)
C(5)-H(5)	0.9500
C(6)-C(7)	1.471(10)
C(7)-H(7A)	0.9900
C(7)-H(7B)	0.9900
C(8)-C(9)	1.509(10)
C(8)-H(8A)	0.9900
C(8)-H(8B)	0.9900
C(9)-C(10)	1.370(9)
C(10)-C(11)	1.394(10)
C(10)-H(10)	0.9500
C(11)-C(12)	1.378(9)
C(11)-H(11)	0.9500
C(12)-C(13)	1.389(9)
C(12)-H(12)	0.9500
C(13)-C(14)	1.514(8)
C(14)-H(14A)	0.9900
C(14)-H(14B)	0.9900
C(15)-H(15A)	0.9900
C(15)-H(15B)	0.9900
Ni(2)-N(2)	2.009(6)
Ni(2)-O(10)	2.026(5)
Ni(2)-O(7)	2.041(5)
Ni(2)-S(5)	2.401(2)
Ni(2)-S(6)	2.410(2)
S(5)-C(25)	1.815(8)
S(5)-C(24)	1.831(8)
S(6)-C(31)	1.795(8)
S(6)-C(32)	1.832(7)
S(7)-O(8)	1.427(5)
S(7)-O(9)	1.428(5)
S(7)-O(7)	1.456(5)
S(7)-C(33)	1.818(9)

S(8)-O(11)	1.417(5)
S(8)-O(12)	1.428(5)
S(8)-O(10)	1.464(5)
S(8)-C(34)	1.825(9)
F(7)-C(33)	1.327(9)
F(8)-C(33)	1.320(8)
F(9)-C(33)	1.324(8)
F(10)-C(34)	1.296(9)
F(11)-C(34)	1.321(9)
F(12)-C(34)	1.344(9)
N(2)-C(26)	1.338(9)
N(2)-C(30)	1.346(9)
C(18)-C(23)	1.377(10)
C(18)-C(19)	1.397(10)
C(18)-H(18)	0.94(5)
C(19)-C(20)	1.389(10)
C(19)-C(32)	1.503(9)
C(20)-C(21)	1.399(10)
C(20)-H(20)	0.9500
C(21)-C(22)	1.379(10)
C(21)-H(21)	0.9500
C(22)-C(23)	1.390(10)
C(22)-H(22)	0.9500
C(23)-C(24)	1.504(10)
C(24)-H(24A)	0.9900
C(24)-H(24B)	0.9900
C(25)-C(26)	1.496(10)
C(25)-H(25A)	0.9900
C(25)-H(25B)	0.9900
C(26)-C(27)	1.380(11)
C(27)-C(28)	1.373(11)
C(27)-H(27)	0.9500
C(28)-C(29)	1.378(10)
C(28)-H(28)	0.9500
C(29)-C(30)	1.371(10)
C(29)-H(29)	0.9500

C(30)-C(31)	1.501(10)
C(31)-H(31A)	0.9900
C(31)-H(31B)	0.9900
C(32)-H(32A)	0.9900
C(32)-H(32B)	0.9900
Cl(1)-C(1S)	1.762(8)
Cl(2)-C(1S)	1.754(8)
C(1S)-H(1SA)	0.9900
C(1S)-H(1SB)	0.9900

N(1)-Ni(1)-O(4)	93.1(2)
N(1)-Ni(1)-O(1)	174.8(2)
O(4)-Ni(1)-O(1)	89.94(19)
N(1)-Ni(1)-S(1)	87.05(16)
O(4)-Ni(1)-S(1)	88.76(14)
O(1)-Ni(1)-S(1)	88.83(14)
N(1)-Ni(1)-S(2)	85.48(16)
O(4)-Ni(1)-S(2)	107.36(14)
O(1)-Ni(1)-S(2)	97.62(14)
S(1)-Ni(1)-S(2)	162.55(8)
N(1)-Ni(1)-C(1)	85.9(2)
O(4)-Ni(1)-C(1)	170.4(2)
O(1)-Ni(1)-C(1)	90.5(2)
S(1)-Ni(1)-C(1)	81.62(17)
S(2)-Ni(1)-C(1)	82.13(17)
C(8)-S(1)-C(7)	101.9(3)
C(8)-S(1)-Ni(1)	93.4(2)
C(7)-S(1)-Ni(1)	101.4(2)
C(14)-S(2)-C(15)	101.1(4)
C(14)-S(2)-Ni(1)	95.2(2)
C(15)-S(2)-Ni(1)	100.2(2)
O(2)-S(3)-O(3)	117.2(3)
O(2)-S(3)-O(1)	112.6(3)
O(3)-S(3)-O(1)	113.8(3)
O(2)-S(3)-C(16)	104.3(4)
O(3)-S(3)-C(16)	105.0(3)

O(1)-S(3)-C(16)	101.9(3)
O(6)-S(4)-O(5)	118.2(3)
O(6)-S(4)-O(4)	114.2(3)
O(5)-S(4)-O(4)	111.6(3)
O(6)-S(4)-C(17)	104.9(3)
O(5)-S(4)-C(17)	103.4(3)
O(4)-S(4)-C(17)	102.3(3)
S(3)-O(1)-Ni(1)	137.8(3)
S(4)-O(4)-Ni(1)	132.5(3)
C(13)-N(1)-C(9)	119.4(6)
C(13)-N(1)-Ni(1)	121.7(4)
C(9)-N(1)-Ni(1)	118.9(5)
C(2)-C(1)-C(6)	121.9(7)
C(2)-C(1)-Ni(1)	102.5(5)
C(6)-C(1)-Ni(1)	101.5(5)
C(2)-C(1)-H(1)	126(4)
C(6)-C(1)-H(1)	112(4)
Ni(1)-C(1)-H(1)	68(4)
C(3)-C(2)-C(1)	118.4(6)
C(3)-C(2)-C(15)	122.6(7)
C(1)-C(2)-C(15)	118.3(6)
C(4)-C(3)-C(2)	121.0(7)
C(4)-C(3)-H(3)	119.5
C(2)-C(3)-H(3)	119.5
C(3)-C(4)-C(5)	119.8(7)
C(3)-C(4)-H(4)	120.1
C(5)-C(4)-H(4)	120.1
C(4)-C(5)-C(6)	121.0(7)
C(4)-C(5)-H(5)	119.5
C(6)-C(5)-H(5)	119.5
C(5)-C(6)-C(1)	117.6(7)
C(5)-C(6)-C(7)	122.3(6)
C(1)-C(6)-C(7)	119.8(7)
C(6)-C(7)-S(1)	109.9(5)
C(6)-C(7)-H(7A)	109.7
S(1)-C(7)-H(7A)	109.7

C(6)-C(7)-H(7B)	109.7
S(1)-C(7)-H(7B)	109.7
H(7A)-C(7)-H(7B)	108.2
C(9)-C(8)-S(1)	116.6(5)
C(9)-C(8)-H(8A)	108.1
S(1)-C(8)-H(8A)	108.1
C(9)-C(8)-H(8B)	108.1
S(1)-C(8)-H(8B)	108.1
H(8A)-C(8)-H(8B)	107.3
N(1)-C(9)-C(10)	121.4(7)
N(1)-C(9)-C(8)	118.2(6)
C(10)-C(9)-C(8)	120.4(6)
C(9)-C(10)-C(11)	119.5(6)
C(9)-C(10)-H(10)	120.2
C(11)-C(10)-H(10)	120.2
C(12)-C(11)-C(10)	118.4(7)
C(12)-C(11)-H(11)	120.8
C(10)-C(11)-H(11)	120.8
C(11)-C(12)-C(13)	119.8(7)
C(11)-C(12)-H(12)	120.1
C(13)-C(12)-H(12)	120.1
N(1)-C(13)-C(12)	121.4(6)
N(1)-C(13)-C(14)	118.6(6)
C(12)-C(13)-C(14)	119.9(6)
C(13)-C(14)-S(2)	116.6(5)
C(13)-C(14)-H(14A)	108.1
S(2)-C(14)-H(14A)	108.1
C(13)-C(14)-H(14B)	108.1
S(2)-C(14)-H(14B)	108.1
H(14A)-C(14)-H(14B)	107.3
C(2)-C(15)-S(2)	109.6(5)
C(2)-C(15)-H(15A)	109.8
S(2)-C(15)-H(15A)	109.8
C(2)-C(15)-H(15B)	109.8
S(2)-C(15)-H(15B)	109.8
H(15A)-C(15)-H(15B)	108.2

F(3)-C(16)-F(1)	107.5(6)
F(3)-C(16)-F(2)	107.1(6)
F(1)-C(16)-F(2)	106.5(7)
F(3)-C(16)-S(3)	111.4(6)
F(1)-C(16)-S(3)	111.9(5)
F(2)-C(16)-S(3)	112.0(5)
F(5)-C(17)-F(6)	107.6(6)
F(5)-C(17)-F(4)	108.4(6)
F(6)-C(17)-F(4)	108.1(7)
F(5)-C(17)-S(4)	112.9(6)
F(6)-C(17)-S(4)	109.8(5)
F(4)-C(17)-S(4)	109.9(5)
N(2)-Ni(2)-O(10)	171.9(2)
N(2)-Ni(2)-O(7)	91.0(2)
O(10)-Ni(2)-O(7)	95.9(2)
N(2)-Ni(2)-S(5)	86.52(16)
O(10)-Ni(2)-S(5)	89.22(15)
O(7)-Ni(2)-S(5)	89.06(14)
N(2)-Ni(2)-S(6)	85.92(17)
O(10)-Ni(2)-S(6)	96.26(16)
O(7)-Ni(2)-S(6)	106.68(14)
S(5)-Ni(2)-S(6)	162.63(8)
C(25)-S(5)-C(24)	102.6(4)
C(25)-S(5)-Ni(2)	92.8(2)
C(24)-S(5)-Ni(2)	101.1(2)
C(31)-S(6)-C(32)	102.0(4)
C(31)-S(6)-Ni(2)	95.1(2)
C(32)-S(6)-Ni(2)	99.2(2)
O(8)-S(7)-O(9)	116.9(3)
O(8)-S(7)-O(7)	113.9(3)
O(9)-S(7)-O(7)	113.5(3)
O(8)-S(7)-C(33)	103.5(4)
O(9)-S(7)-C(33)	103.7(3)
O(7)-S(7)-C(33)	102.9(3)
O(11)-S(8)-O(12)	117.0(3)
O(11)-S(8)-O(10)	113.4(3)

O(12)-S(8)-O(10)	113.8(3)
O(11)-S(8)-C(34)	104.7(4)
O(12)-S(8)-C(34)	105.2(4)
O(10)-S(8)-C(34)	100.4(4)
S(7)-O(7)-Ni(2)	131.5(3)
S(8)-O(10)-Ni(2)	134.4(3)
C(26)-N(2)-C(30)	120.2(6)
C(26)-N(2)-Ni(2)	119.5(5)
C(30)-N(2)-Ni(2)	120.3(5)
C(23)-C(18)-C(19)	121.4(7)
C(23)-C(18)-H(18)	122(4)
C(19)-C(18)-H(18)	116(4)
C(20)-C(19)-C(18)	119.0(7)
C(20)-C(19)-C(32)	121.9(7)
C(18)-C(19)-C(32)	118.2(7)
C(19)-C(20)-C(21)	119.3(7)
C(19)-C(20)-H(20)	120.4
C(21)-C(20)-H(20)	120.4
C(22)-C(21)-C(20)	121.0(7)
C(22)-C(21)-H(21)	119.5
C(20)-C(21)-H(21)	119.5
C(21)-C(22)-C(23)	119.6(7)
C(21)-C(22)-H(22)	120.2
C(23)-C(22)-H(22)	120.2
C(18)-C(23)-C(22)	119.4(7)
C(18)-C(23)-C(24)	118.2(7)
C(22)-C(23)-C(24)	122.0(7)
C(23)-C(24)-S(5)	109.2(5)
C(23)-C(24)-H(24A)	109.8
S(5)-C(24)-H(24A)	109.8
C(23)-C(24)-H(24B)	109.8
S(5)-C(24)-H(24B)	109.8
H(24A)-C(24)-H(24B)	108.3
C(26)-C(25)-S(5)	116.5(5)
C(26)-C(25)-H(25A)	108.2
S(5)-C(25)-H(25A)	108.2

C(26)-C(25)-H(25B)	108.2
S(5)-C(25)-H(25B)	108.2
H(25A)-C(25)-H(25B)	107.3
N(2)-C(26)-C(27)	121.5(7)
N(2)-C(26)-C(25)	118.7(7)
C(27)-C(26)-C(25)	119.8(7)
C(28)-C(27)-C(26)	118.4(8)
C(28)-C(27)-H(27)	120.8
C(26)-C(27)-H(27)	120.8
C(27)-C(28)-C(29)	119.9(8)
C(27)-C(28)-H(28)	120.0
C(29)-C(28)-H(28)	120.0
C(30)-C(29)-C(28)	119.4(7)
C(30)-C(29)-H(29)	120.3
C(28)-C(29)-H(29)	120.3
N(2)-C(30)-C(29)	120.6(7)
N(2)-C(30)-C(31)	119.5(6)
C(29)-C(30)-C(31)	119.9(7)
C(30)-C(31)-S(6)	116.7(5)
C(30)-C(31)-H(31A)	108.1
S(6)-C(31)-H(31A)	108.1
C(30)-C(31)-H(31B)	108.1
S(6)-C(31)-H(31B)	108.1
H(31A)-C(31)-H(31B)	107.3
C(19)-C(32)-S(6)	109.1(5)
C(19)-C(32)-H(32A)	109.9
S(6)-C(32)-H(32A)	109.9
C(19)-C(32)-H(32B)	109.9
S(6)-C(32)-H(32B)	109.9
H(32A)-C(32)-H(32B)	108.3
F(8)-C(33)-F(9)	107.7(7)
F(8)-C(33)-F(7)	107.6(6)
F(9)-C(33)-F(7)	108.2(7)
F(8)-C(33)-S(7)	111.1(6)
F(9)-C(33)-S(7)	110.1(5)
F(7)-C(33)-S(7)	111.9(6)

F(10)-C(34)-F(11)	110.3(8)
F(10)-C(34)-F(12)	107.9(7)
F(11)-C(34)-F(12)	106.4(6)
F(10)-C(34)-S(8)	112.2(6)
F(11)-C(34)-S(8)	110.4(6)
F(12)-C(34)-S(8)	109.4(6)
Cl(2)-C(1S)-Cl(1)	110.7(4)
Cl(2)-C(1S)-H(1SA)	109.5
Cl(1)-C(1S)-H(1SA)	109.5
Cl(2)-C(1S)-H(1SB)	109.5
Cl(1)-C(1S)-H(1SB)	109.5
H(1SA)-C(1S)-H(1SB)	108.1
